# Selective imaging of cathepsin L in breast cancer by fluorescent activity-based probes†
†Electronic supplementary information (ESI) available. See DOI: 10.1039/c7sc04303a


**DOI:** 10.1039/c7sc04303a

**Published:** 2018-01-16

**Authors:** Marcin Poreba, Wioletta Rut, Matej Vizovisek, Katarzyna Groborz, Paulina Kasperkiewicz, Darren Finlay, Kristiina Vuori, Dusan Turk, Boris Turk, Guy S. Salvesen, Marcin Drag

**Affiliations:** a Department of Bioorganic Chemistry , Faculty of Chemistry , Wroclaw University of Technology , Wyb. Wyspianskiego 27 , 50-370 Wroclaw , Poland . Email: marcin.poreba@pwr.edu.pl ; Email: marcin.drag@pwr.edu.pl; b Sanford Burnham Prebys Medical Discovery Institute , 10901 North Torrey Pines Road , La Jolla , CA 92037 , USA; c Department of Biochemistry and Molecular and Structural Biology , Jožef Stefan Institute , SI-1000 Ljubljana , Slovenia

## Abstract

Cysteine cathepsins normally function in the lysosomal degradation system where they are critical for the maintenance of cellular homeostasis and the MHC II immune response, and have been found to have major roles in several diseases and in tumor progression. Selective visualization of individual protease activity within a complex proteome is of major importance to establish their roles in both normal and tumor cells, thereby facilitating our understanding of the regulation of proteolytic networks. A generally accepted means to monitor protease activity is the use of small molecule substrates and activity-based probes. However, there are eleven human cysteine cathepsins, with a few of them displaying overlapping substrate specificity, making the development of small molecules that selectively target a single cathepsin very challenging. Here, we utilized HyCoSuL, a positional scanning substrate approach, to develop a highly-selective fluorogenic substrate and activity-based probe for monitoring cathepsin L activity in the breast cancer cell line MDA-MB-231. Use of this probe enabled us to distinguish the activity of cathepsin L from that of other cathepsins, particularly cathepsin B, which is abundant and ubiquitously expressed in normal and transformed cell types. We found that cathepsin L localization in MDA-MB-231 cells greatly overlaps with that of cathepsin B, however, several cathepsin L-rich lysosomes lacked cathepsin B activity. Overall, these studies demonstrate that HyCoSuL-derived small molecule probes are valuable tools to image cathepsin L activity in living cells. This approach thus enables evaluation of cathepsin L function in tumorigenesis and is applicable to other cysteine cathepsins.

## Abstract 2

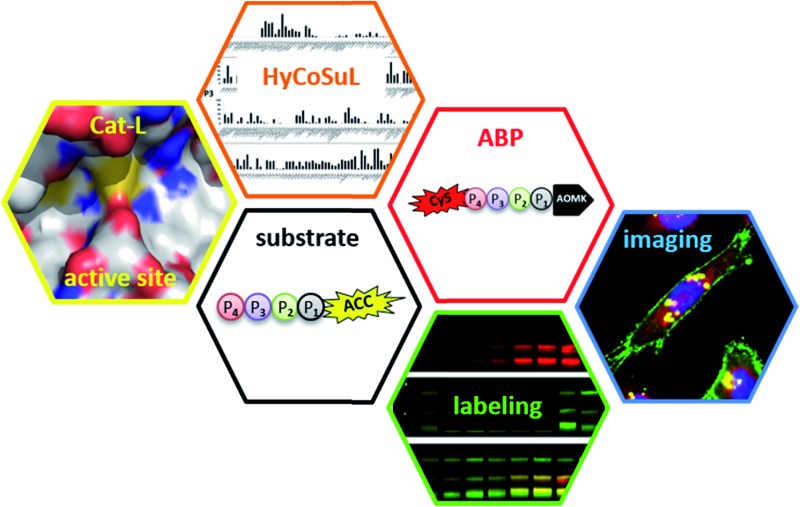
Highly-selective fluorogenic substrate and activity-based probe for monitoring cathepsin L activity in the breast cancer cell line MDA-MB-231.

## 1. Introduction

Cysteine cathepsins are a group of eleven structurally related proteases in humans and are normally found in lysosomes that are responsible for intracellular protein degradation, autophagy and immune response.^1^ Cysteine cathepsins also have major roles in a number of diseases, including cancer, arthritis and osteoporosis, and are therefore considered of major importance in drug development.^2^–^4^ Cathepsin L is ubiquitously expressed and is one of the most abundant cathepsins.^3^ It is synthesized as a proenzyme that is activated in the acidic milieu of the endolysosomal system autocatalytically^5^ or by other proteases.^3^ It is an endopeptidase with a highest catalytic activity at pH 5.0–6.0 6,7 and displays broad substrate specificity.^3^,^8^ In addition to its role in protein degradation, cathepsin L was found to be critical for MHC II-mediated antigen processing and presentation in the thymus.^9^ Furthermore, cathepsin L was found to have a major role in the nucleus, in processing the H3 histone^10^ as well as the Cux-1 transcription factor,^11^ and has an important role in keratinocyte differentiation.^12^ Similarly to several other cathepsins, cathepsin L was found to have an important role in the progression of cancer. However, in contrast to cathepsins B and S that are present in both stromal cells, such as macrophages, and tumor cells, cathepsin L is primarily secreted by tumor cells.^13^–^15^

Cathepsin L was suggested to participate in tumor cell mechanisms underlying chemo-resistance.^16^ Active forms of cathepsin L found in the nucleus reportedly serve as either tumor promoters^17^,^18^ or suppressors^19^ depending on the microenvironment. Finally, secreted cathepsin L participates in the degradation of extracellular proteins like the insulin receptor, matrix fibronectin, elastin and collagen,^13^,^20^ as well as several membrane anchored cell adhesion molecules (CAMs) and receptors,^21^ thereby additionally contributing to tumorigenesis.

While in mice a single cathepsin L gene exists, in humans there are two closely related cathepsin L orthologues, cathepsin L and cathepsin V. Both cathepsins L and V have maximum activity in the same pH range and share 79.5% amino acid identity.^22^,^23^ Moreover, several functions originally attributed to cathepsin L based on mouse studies were found to be taken over by cathepsin V in humans, including the MHC II-mediated immune response in the thymus.^24^ This is likely also true for the nuclear functions of cathepsin L, as human cathepsin V, but not cathepsin L, was found to bind DNA.^25^ However, a thorough validation is still lacking. Moreover, the two cathepsins were found to have very similar substrate specificities, as revealed by the classical Positional Scanning Synthetic Combinatorial Library (PS-SCL).^8^ In addition, not only cathepsin V, but also other cathepsins, have similar and largely overlapping substrate specificities to cathepsin L,^8^ which was also confirmed by several proteomic studies.^26^,^27^ Hence, a cathepsin L selective peptide motif is still to be discovered.

In order to be able to discriminate between cathepsin L and related cathepsins and to correctly address their physiological functions, one has to understand their specificity.^28^ This is also of crucial importance for the development of selective and potent inhibitors and of appropriate tools for monitoring their activity *in situ*. Among the latter are selective substrates and activity-based probes (ABPs).^29^–^31^ All early substrates aimed to target cathepsin L were unspecific and were efficiently hydrolyzed by cathepsin B and other proteases, and were largely useful for detection of cathepsin activity in biological samples rather than probes for cathepsin L.^32^,^33^ Similarly, the cresyl violet peptidic substrates, which allow monitoring of cathepsin activities in cells, are based on nonselective peptidic motifs.^34^ A major step forward was application of the reverse-design principle, which is based on the conversion of a selective optimized inhibitor into a cleavable substrate by replacement of the warhead with a quencher–fluorophore pair. This led to the development of selective cathepsin S substrates,^35^,^36^ whereas selective substrates for other proteases are still lacking.

ABPs are mostly short peptides coupled to a reactive group (warhead) that binds to catalytic machinery, with a detectable tag (biotin, a radioisotope or a fluorophore).^37^,^38^ Use of ABPs in protease and related enzyme studies was first proposed by Powers^39^ and later exploited by others.^40^–^43^ Application of ABPs to analysis of cysteine cathepsins was pioneered by the Bogyo group, who initially designed probes based on simple broad-spectrum radiolabeled inhibitors,^44^ although fluorophore-labeled probes^45^ and quenched activity-based probes have also been applied to living cells and whole organisms.^46^,^47^ Some successes have been achieved in the design of selective cathepsin S probes,^48^,^49^ but selective tools for other cathepsins remain to be developed.

Currently, the most successful approach to rapidly identify sequences with high activity and selectivity for individual proteases is HyCoSuL, which utilizes a combination of natural and unnatural amino acids and positional scanning library technology to explore an extended chemical space around protease active sites.^40^,^50^–^52^ We used two library scanning procedures to obtain highly active substrates for cathepsin L. In the first, we employed a fixed P1 Arg HyCoSuL to dissect cathepsin L specificity in the P4–P2 positions. In the second, we also utilized a P1 individual substrate library to explore cathepsin L preferences in the P1 position. With these two types of libraries we were able to develop potent and highly selective fluorogenic substrates and ABPs for cathepsin L. Finally, we confirmed the utility of the identified probes by defining cathepsin L activity and localization in the breast cancer line MDA-MB-231.

## 2. Experimental

### 2.1. Reagents

All chemicals were purchased from commercial suppliers and used without further purification. For ACC-labeled individual substrate synthesis, we used Rink amide RA resin (loading 0.48 mmol g^–1^, Iris Biotech GmbH), Fmoc-protected amino acids (purity > 98%, Iris Biotech GmbH, Bachem, Creosalus, P3 BioSystems and QM Bio), *N*-hydroxybenzotriazole (HOBt monohydrate purity > 98%, Creosalus), *N*,*N*-diisopropylethylamine (DIPEA, peptide grade, Iris Biotech GmbH), diisopropylcarbodiimide (DICI, peptide grade, Iris Biotech GmbH), HATU and HBTU (peptide grade, ChemPep Inc.), 2,4,6-trimethylpyridine (2,4,6-collidine, peptide grade, Sigma-Aldrich Sp. z o.o.), *N*,*N*′-dimethylformamide (DMF, peptide grade, WITKO Sp. z o.o.), acetonitrile (ACN, HPLC pure, WITKO Sp z o.o.), piperidine (PIP, peptide grade, Iris Biotech GmbH), trifluoroacetic acid (TFA, purity 99%, Iris Biotech GmbH), triisopropylsilane (TIPS, purity 99%, Sigma-Aldrich Sp. z o.o.), methanol (MeOH, pure for analysis, POCh), dichloromethane (DCM, pure for analysis, POCh), diethyl ether (Et_2_O, pure for analysis, POCh), acetic acid (AcOH, purity 98%, POCh) and phosphorus pentoxide (P_2_O_5_, purity 98%, POCh). Fmoc-6-ahx-OH, biotin and 2-chlorotrityl chloride resin (100–200 mesh, 1.59 mmol g^–1^) were purchased from Iris Biotech GmbH. TFE (2,2,2-trifluoroethanol), HBr (30% wt. in AcOH), anhydrous THF (tetrahydrofuran), NMM (4-methylmorpholine), IBCF (isobutylchloroformate) and 2,6-DMBA (2,6-dimethylbenzoic acid) were from Sigma-Aldrich. Fluorescent tags (Cyanine5 NHS/Cy5-NHS, Cyanine3 NHS/Cy3-NHS and BODIPY-FL NHS/BDP_FL_-NHS) were purchased from Lumiprobe. Diazomethane was generated according to the Aldrich Technical Bulletin (AL-180) protocol.

### 2.2. Preparation of recombinant cathepsins B, L, K, V and S

A detailed description of expression and purification can be found elsewhere.^53^,^54^ The concentration of active cathepsins was determined *via* active site titration with E-64 inhibitor (Peptide Institute).

### 2.3. P4–P2 hybrid combinatorial substrate library (HyCoSuL) synthesis

A detailed protocol for the synthesis of the P4–P2 fluorogenic substrate combinatorial library is provided elsewhere.^55^

### 2.4. Enzymatic kinetic studies

All kinetic experiments were performed using fMax (Molecular Devices) and CLARIOStar (BMG LABTECH) spectrofluorimeters operating in kinetic mode using 96-well plates. ACC-labeled substrates were screened using 355 nm and 460 nm wavelengths (excitation and emission, respectively). The cathepsin assay buffer (for the library, substrates and kinetic inhibitors) contained 100 mM sodium acetate, 100 mM sodium chloride, 10 mM DTT and 1 mM EDTA and had a pH of 5.5. The buffer was prepared at room temperature and the enzyme kinetic studies were performed at 37 °C.

### 2.5. Characterization of cathepsin L P4–P2 substrate specificity using HyCoSuL

P4, P3 and P2 sub-libraries were each screened at 100 μM concentration with cathepsin L in 100 μL final volume. The active enzyme concentration was in the 0.5–2.0 nM range depending on the sub-library. The assay time was 30 min, but only the linear portions of the progression curves (5–15 min) were used for the velocity (RFU s^–1^) calculations. Each sub-library screening was repeated at least 3 times, and the average value was used to create the substrate specificity matrix. The best recognized amino acid at each position was set to 100% and other amino acids were normalized accordingly.

### 2.6. Characterization of cathepsin L P1 substrate specificity

To determine cathepsin L preferences in the P1 position we used a fluorogenic substrate library of 139 components with the general formula Ac-Ala-Arg-Leu-P1-ACC. The library contains 19 natural and 120 unnatural amino acids. The synthesis and detailed structure of the library are found elsewhere.^51^ The library was screened with cathepsin L at two concentrations: 4 μM (below the *K*_M_ values) and 200 μM (above the *K*_M_ values). The substrate cleavage assay was carried out for ∼30 min, but only the linear portions of the hydrolysis curves (10–15 min) were analyzed. Both screenings were repeated at least three times and average values are presented. The Ac-Ala-Arg-Leu-Arg-ACC substrate served as a positive control, and the rate of its hydrolysis was set as 100%. Values for the other substrates were normalized to the P1 Arg substrate.

### 2.7. Synthesis of individual optimized substrates

Each ACC-labeled cathepsin L substrate analyzed was synthesized and purified as described elsewhere.^56^

### 2.8. Screening of potential cathepsin L-selective substrates with other cathepsins

All potentially selective cathepsin L substrates were tested for their selectivity against a panel of five different cathepsins, containing cathepsins L, V, S, K and V, at concentrations of 2 μM and 100 μM. To obtain reliable results the concentration of each cathepsin was 5 nM. Substrate hydrolysis was carried out for 10 min (for cathepsin L) and for up to 60 min (for other cathepsins). Hydrolysis rates (RFU s^–1^) are presented as average values from at least three screenings.

### 2.9. Determination of kinetic parameters (*k*_cat_, *K*_M_ and *k*_cat_/*K*_M_) for individual substrates

Classic substrate velocity plots were analyzed according to Michaelis–Menten kinetics and a detailed protocol for the determination of kinetic parameters for ACC-tagged substrates is found elsewhere.^56^ Because the substrates demonstrated a high degree of selectivity for cathepsin L, we had to employ higher concentrations of other cathepsins to observe substrate hydrolysis and thus the following cathepsin concentrations were used: cathepsin L, 0.1–0.5 nM; cathepsin B, 4–15 nM; cathepsin K, 5–20 nM; cathepsin V, 10–20 nM; and cathepsin S, 5–20 nM. Experiments were repeated at least three times and results are presented as an average. The SDs were below 15%.

### 2.10. Synthesis of irreversible activity-based probes

Two biotin-labeled probes (MP-cL1 and MP-cL2) were synthesized as described by Poreba *et al.*^30^ In brief, a biotin-6-ahx-His(Trt)-dThr(*t*Bu)-Phe(F_5_)-OH peptide was synthesized on a solid support using a 2-chlorotrityl chloride resin and used for further synthesis without purification. In parallel, two amino acyl-warhead amino acids were conjugated to electrophilic binding groups. Boc-Lys(Cbz)-AOMK and Boc-Arg(Cbz)_2_-AOMK were synthesized in solution using diazomethane in diethyl ether (Lys/Arg-CH_2_N_2_), HBr in AcOH (Lys/Arg-CH_2_Br) and 2,6-DMBA in DMF (Lys/Arg-AOMK). Next, Boc-Lys(Bzl)-AOMK and Boc-Arg(Cbz)_2_-AOMK were N-terminally de-protected in TFA/DCM (1 : 1 v/v with 3% TIPS, 30 min) and coupled with the above peptide to obtain (1) biotin-6-ahx-His(Trt)-dThr(*t*Bu)-Phe(F_5_)-Lys(Cbz)-AOMK and (2) biotin-6-ahx-His(Trt)-dThr(*t*Bu)-Phe(F_5_)-Arg(Cbz)_2_-AOMK, followed by purification on HPLC. Next, Trt/*t*Bu protecting groups were removed in TFA/DCM and Cbz groups were removed *via* hydrogenolysis (H_2_, Pd/C, DMF). The final compounds were purified again on HPLC. Fluorescence probes were similarly synthesized. Cy5-labeled MP-cL3 probe: Boc-6-ahx-His(Trt)-dThr(*t*Bu)-Phe(F_5_)-OH peptide was synthesized on a solid support and used without further purification. Boc-Cys(Bzl)-AOMK was synthesized and N-terminally de-protected as described above. Next, the peptide and H_2_N-Cys(Bzl)-AOMK were coupled in DMF to obtain Boc-6-ahx-His(Trt)-dThr(*t*Bu)-Phe(F_5_)-Cys(Bzl)-AOMK. All protecting groups were then removed in TFA/DCM (1 : 1 v/v with 3% TIPS, 30 min), and excess TFA was removed in a rotary evaporator under reduced pressure (the mixture was washed several times with DCM). The crude product (1 eq.) was dried over P_2_O_5_ in a desiccator for several hours. In a separate flask, 1 eq. of Cy5-NHS was dissolved in DMF and 5 eq. of DIPEA were then added. The solution was immediately mixed with crude peptide-AOMK (1 eq.) and the reaction was carried out at room temperature for 30 min. The reaction progress was monitored by HPLC (220 nm for peptide-AOMK or 646 nm for Cy5-NHS). The final product was purified using HPLC to obtain Cy5-6-ahx-His-dThr-Phe(F_5_)-Cys(Bzl)-AOMK. Cy5/BDP_FL_/Cy3-labeled MP-pc1/2/3 pan-cathepsin specific probes: first, the Boc-6-ahx-Ala-Arg(Pbf)-Leu-OH peptide was synthesized on a solid support and used without further purification. In parallel, Boc-Arg(Boc)_2_-AOMK was synthesized and N-terminally de-protected as described above. Next, the peptide and warhead were coupled in DMF to obtain Boc-6-ahx-Ala-Arg(Pbf)-Leu-Arg(Boc)_2_-AOMK. The crude product was then purified using HPLC, de-protected with TFA/DCM and dried over P_2_O_5_ to obtain H_2_N-6-ahx-Ala-Arg-Leu-Arg-AOMK. Finally, the fluorescent tag (BDP_FL_/Cy3) was attached to the N-terminus and the product was purified using HPLC to obtain BDP_FL_-6-ahx-Ala-Arg-Leu-Arg-AOMK (MP-pc2) and Cy3-6-ahx-Ala-Arg-Leu-Arg-AOMK (MP-pc3). Cy5-Ala-Arg-Leu-Arg-AOMK (MP-pc1) was synthesized without a linker and thus first the Boc-Ala-Arg(Pbf)-Leu-OH peptide was synthesized and then coupled to the H_2_N-Arg-AOMK warhead. Next, the compound was de-protected and labeled with a Cy5 tag in a manner similar to that for the BDP_FL_ and Cy3 probes.

### 2.11. Determination of kinetic parameters (*k*_obs_/*I*) for activity-based probes

For the kinetic studies, five recombinant human cathepsins (B, L, V, K and S) were expressed and purified and active site titrated using an E64 inhibitor. The *k*_obs_/*I* parameter was measured under pseudo-first order kinetic conditions, as described.^40^ The cathepsin of interest was mixed with various concentrations of probe (at least 5-fold excess over cathepsin) and 100 μM ACC-fluorescence substrate. The experiments were repeated at least three times, and the results are presented as an average. The SDs were below 20%.

### 2.12. Detection of cathepsin L in HEK293T cells with biotinylated ABPs

To evaluate the applicability of the bMP-cL1 and bMP-cL2 probes using affinity-based enrichment assays, HEK293T cells were seeded on two 10 cm plates and cultured for 2 days. The medium was changed and the cells were then transfected using the pcDNA3:ppCatL construct (prepared in-house for expression of human preprocathepsin L in mammalian cells) and Lipofectamine 2000 ™ based on the manufacturer’s guidelines (Thermo Scientific). Forty-eight hours later, the cells were harvested and whole cell lysates were prepared in a lysis buffer maintaining the cathepsin activity (50 mM sodium phosphate, 100 mM NaCl, 1 mM EDTA, 1 mM DTT, 0.5% NP-40 (nonyl phenoxypolyethoxylethanol) and pH 6.0) as described elsewhere.^26^ Non-transfected HEK293T cells served as controls. The transfection efficiency was calculated by measuring the cathepsin L activity increase in HEK293T cells after transfection. For cathepsin L labeling, cell lysates were mixed with various (0–10 μM) concentrations of MP-cL1 and MP-cL2 probes and incubated for 2 hours at 37 °C. To control for non-specific binding, 25 μM E-64d, a pan-cathepsin cell permeable inhibitor was added to lysates 1 hour prior to probe addition. After incubation samples were mixed with 6× SDS-PAGE loading buffer and analyzed by immunoblotting. Nitrocellulose membranes were blocked after transfer with 5 mL 3% BSA (bovine serum albumin) in PBS, pH 7.4, for 1 hour before the streptavidin–HRP conjugate was added to the membranes at a 1 : 2000 dilution, according to the manufacturer’s guidelines (Alpha diagnostics). Membranes were incubated for 45 minutes with gentle rocking and then washed 4 times for 15 min in 10 mL PBS, pH 7.4. Membranes were then developed using standard ECL detection reagents (GE Healthcare), and signals were detected on bioluminescence sensitive film (Kodak).

### 2.13. ABP uptake into cells

To evaluate probe uptake, 250 000 HEK293T cells transfected with pcDNA3:ppCatL were seeded into 6-well plates and incubated with various concentrations of bMP-cL1 and bMP-cL2 (0–10 μM) for up to 24 hours. In control experiments the cells were preincubated with 25 μM E-64d, a pan-cathepsin inhibitor, for 2 hours prior to probe addition. After incubation samples were collected and immunoblotted. Biotinylated proteins were detected as described above.

### 2.14. Evaluation of probe selectivity by immunocapture

To evaluate MP-cL1 and MP-cL2 selectivity, 250 000 HEK293T cells overexpressing cathepsin L were plated and treated with probes and inhibitors as described above. Streptavidin-bound magnetic beads were prepared in separate tubes according to the manufacturer’s guidelines (Pierce™ Streptavidin Magnetic Beads, Thermo Scientific). Briefly, 50 μL beads were washed with sample lysis buffer and then the lysates were added. Tubes were incubated at room temperature on a rocking platform for 2 hours. The beads were then extensively washed with 50 mM Tris-buffered saline containing 0.1% Tween-20 and collected between each washing step. The beads were then re-suspended in 1× SDS loading buffer at a final volume of 100 μL, and the samples were placed at 95 °C and prepared for immunoblotting as described above to detect biotinylated proteins in the lysates, as well as in the unbound and eluted fractions. To confirm cathepsin L/B selectivity, we seeded HEK293T cells as described above. The probes were added at a 5 μM final concentration and incubated for 18 hours before the cells were harvested and lysed to isolate biotinylated proteins using streptavidin magnetic beads, as described above. The lysates plus the unbound and eluting fractions were then evaluated to detect cathepsin B (in-house mouse polyclonal Ab;^51^ dilution 1 : 1000) and cathepsin L (Abcam ab62710).

### 2.15. Cathepsin reactivity with MP-cL3 and MP-pc1 ABPs based on SDS-PAGE analysis

To determine the potency and selectivity of the Cy5-probes, each active site-titrated cathepsin was preincubated separately in the assay buffer for 15 min at 37 °C at a concentration of 10 nM (experiment A) or 100 nM (experiment B), and then mixed with various concentrations of either the MP-cL3 or MP-pc1 probe for 30 min. In experiment A, the probe concentrations were 2 nM, 10 nM, 50 nM and 250 nM, while in experiment B the probe concentrations were 20 nM, 100 nM, 500 nM and 2.5 μM. The reaction volume for all the samples was 200 μL. After 30 min, 100 μL of 3× SDS/DTT was added to the reaction mixtures, the samples were boiled for 5 min, and then 20 μL of the sample was run on 4–12% Bis-Tris Plus 15-well gels for 30 min at 200 V with 2 μL of PageRuler™ Prestained Protein Ladder. The gels were then directly scanned at 700 nm (red channel for Cy5 detection; excitation 685 nm) using an Odyssey fluorescence imaging system (LI-COR) and the images were analyzed using Image Studio software.

### 2.16. Detection of cathepsin L in MDA-MB-231 cells by SDS-PAGE and western blotting

Low passage MDA-MB-231 cells were seeded on 12-well plates (200 000 cells per well per 1 mL of medium) and cultured in Dulbecco’s Modification of Eagle’s Medium (DMEM) supplemented with 10% fetal bovine serum, 2 mM of l-glutamine, 100 units per mL penicillin and 100 μg mL^–1^ streptomycin. The next day, the MP-cL3 and MP-pc1 probes (1 μM) were added to the medium and the cells were incubated for 0–18 hours. To control for non-specific binding, the cells were preincubated with E64d (25 μM) for 2 hours prior to probe addition. At various time points, the medium was aspirated and the cells were gently washed twice with 1 mL of Dulbecco’s Phosphate-Buffered Saline (DPBS) and then trypsinized, harvested and centrifuged for 10 min (500 × *g*). The supernatants were removed by aspiration and the pellets washed with 1 mL of DPBS and centrifuged (5 min, 500 × *g*). The supernatants were again removed and the pellets solubilized in 100 μL 1× SDS/DTT and then boiled for 5 min. Each sample was then cooled to room temperature and sonicated (Branson Sonifier 250, duty cycle: 50, output control: 5, cycles: 5) and then 30 μL was loaded on 4–12% Bis-Tris Plus gels and run for 30 min at 200 V with a SDS-PAGE Standards/Broad Range ladder. Proteins were transferred to a 0.2 μm nitrocellulose membrane for 1 hour at 10 V and then Ponceau staining was used to verify equal loading. Membranes were blocked with 2.5% BSA in Tris-buffered saline/0.1% v/v Tween 20 (TBS-T) for 1 hour at room temperature. Cathepsins L and B were detected by primary goat anti-hCathepsin L (R&D, AF952, 0.2 mg mL^–1^, 1 : 2000) and goat anti-hCathepsin B (R&D, AF953, 0.2 mg mL^–1^, 1 : 1000) antibodies, respectively, followed by incubation with a secondary antibody (IRDye® 800CW, LI-COR, donkey anti-goat, 1 : 10 000). The membranes were incubated with primary antibodies for 2 hours, and with (fluorescent) secondary antibodies for 30 min (both in TBS-T at room temperature). The fluorescence was read at 700 nm (Cyanine5; excitation 685 nm) and 800 nm (secondary antibody; excitation 785 nm) using an Odyssey fluorescence imaging system (LI-COR). The images were analyzed using Image Studio software.

### 2.17. Detection of cathepsin L in MDA-MB-231 cells by immunofluorescence

Glass coverslips 15 mm in diameter were placed in 12-well plates and coated with poly-d-lysine (500 μL, 1 mg mL^–1^ in water, Specialty Media) for 30 minutes. The poly-d-lysine was aspirated and the coverslips were washed twice with sterile water and air-dried. Next, low passage MDA-MB-231 cells in DMEM (100 000 cells per mL) were added to wells (1 mL per well) and allowed to attach for 2 hours. Cells were then treated with Cy5-labeled probes (1 μM) for 8 or 24 hours. The control cells were preincubated with E64d for 2 hours. After various incubation times, the medium was removed and the cells were gently washed twice with DPBS (1 mL each). The cells were then fixed with ice-cold methanol (500 μL per well) for 15 minutes at –20 °C. The methanol was gently removed and the fixed cells were washed twice with DPBS, blocked and permeabilized with 10% BSA in DPBS (with 0.5% Triton-X, v/v) for 1 hour at room temperature, and washed twice with 3% BSA in DPBS (with 0.5% Triton-X, v/v). Cathepsins L and B were detected by primary goat anti-hCathepsin L (R&D, AF952, 0.2 mg mL^–1^, 1 : 200) and goat anti-hCathepsin B (R&D, AF953, 0.2 mg mL^–1^, 1 : 80), respectively, in 3% BSA in DPBS (with 0.5% Triton-X, v/v) for 2 hours at room temperature. The antibodies were removed and the cells were washed twice with 3% BSA in DPBS (with 0.5% Triton-X, v/v) and labeled with a secondary antibody (Alexa Fluor™ 488 donkey anti-goat IgG (H + L), A11055, Invitrogen, 1 : 500) in DPBS (with 0.5% Triton-X, v/v) for 1 hour at room temperature. Next, the cells were washed twice with DPBS, and coverslips were mounted using Vectashield mounting medium fluorescence containing DAPI (Vector Lab. H-1000). In brief, a drop of mounting medium was placed on the Superfrost Plus microscope slide (Fisher Scientific, no. 12-550-15) and the coverslip was placed on the drop upside-down. Excess mounting medium was removed with a kimwipe and nail polish was used to seal the coverslips before subjecting the slides to confocal microscope analysis (Axio Observer.A1 Inverted Microscope, Zeiss, with BD Carv II confocal unit, objective 40× and Zeiss LSM 710 NLO Confocal Microscope, objective PlanApochromat 63×/1.4 DIC oil). DAPI was detected using the UV channel, the Cy5 probe was detected with a Cy5 filter (excitation at 647 nm (Axio Observer) or 633 nm (Zeiss LSM), emission at 667 nm; red channel) and cathepsins/secondary antibodies were read using FITC filters (excitation at 495 nm (Axio Observer) or 488 nm (Zeiss LSM), emission at 517 nm; red channel; green channel). All images were acquired in .tiff format in MetaMorph software or in .lsm format in ZEN software and analyzed using ImageJ and ZEN software. The images are representative views of the cells from two coverslips.

### 2.18. Weighted colocalization coefficients

To quantify ABP/cathepsin colocalization we calculated weighted colocalization coefficients between the MP-cL3 ABP (red) and cathepsin L (green) and between the MP-cL3 ABP (red) and cathepsin B (green) using ZEN 2011 software. These calculations were performed by summing the pixels in the colocalized regions (red and green) and dividing by the sum of the pixels in the red channel (ABP). The value of each pixel was equal to its intensity value (from 0 to 1), thus a weighted colocalization coefficient is more accurate here than the standard colocalization coefficient where all the pixels, regardless of their intensity, have a value of 1. To eliminate the red and green staining background in these calculations, we set crosshairs according to single label controls. A graphical example of the calculated weighted colocalization coefficient is presented in Fig. S12.†

## 3. Results & discussion

### 3.1. P4–P1 combined approach with unnatural amino acids

**Fig. 1:**
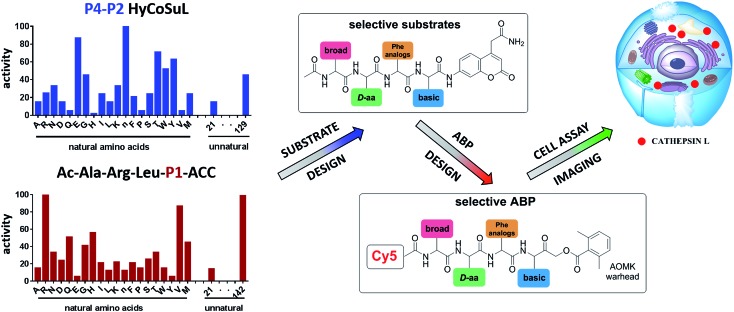
A general procedure to optimize cathepsin L fluorogenic substrates and ABPs. P4–P2 enzyme preferences are determined using previously reported HyCoSuL technology, and the P1 position is screened using a fixed P4–P3–P2 sequence but varying P1 (Ac-Ala-Arg-Leu-P1-ACC), allowing determination of the full substrate specificity profile (P4–P1). Next, the most selective substrate is converted into a fluorescent-labeled ABP which allows for the selective imaging of cathepsin L in cells.

To develop novel active and selective substrates and ABPs for cathepsin L, we undertook a combined approach employing HyCoSuL and a P1-individual substrate library containing unnatural amino acids. Fig. 1 shows an outline of this method.

### 3.2. P4–P2 hybrid combinatorial substrate library

**Fig. 2:**
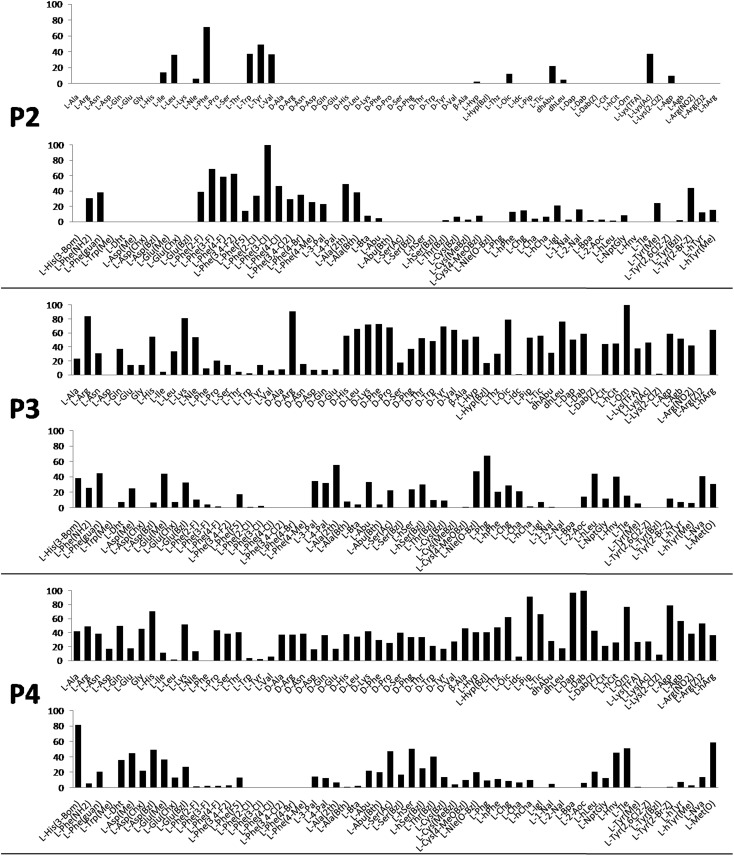
Human cathepsin L preferences at the P4, P3 and P2 positions. Human cathepsin L preferences were determined using three HyCoSuL sub-libraries, Ac-P4-Mix-Mix-Arg-ACC, Ac-Mix-P3-Mix-Arg-ACC and Ac-Mix-Mix-P2-Arg-ACC, all containing natural and unnatural amino acids. The *x* axis shows the abbreviated names of the amino acids and the *y* axis displays the relative activity presented as a percentage of the best-recognized amino acid. Standard deviations calculated from three screenings were 15% of values shown in the figure.

Cathepsin L substrate specificity was determined using a substrate combinatorial library containing Arg at P1.^55^ Arg is reportedly recognized by cysteine cathepsins in the S1 pocket.^8^ The overall library architecture was Ac-P4-P3-P2-Arg-ACC, and the library was composed of three sub-libraries: P4, Ac-X-Mix-Mix-Arg-ACC; P3, Ac-Mix-X-Mix-Arg-ACC; and P2, Ac-Mix-Mix-X-Arg-ACC (Mix: an equimolar mixture of natural amino acids, omitting cysteine and substituting norleucine for methionine). In addition to natural amino acids, we also used 101 unnatural derivatives that can explore extended chemical space around the cathepsin L active site pockets. As a fluorescent tag in this library (and all individual substrates for cathepsin L) we selected an ACC (7-amino-4-carbamoylcoumarin) reporter. This group is widely used for the generation of fluorescent substrates for proteases due to its high quantum yield and great signal-to-noise ratio. Moreover, ACC when quenched by a peptide bond displays almost non-detectable fluorescence.^57^,^58^ The library was screened with cathepsin L and the data were used to create a broad substrate specificity matrix (Fig. 2). Screening the enzyme with the natural amino acid library confirmed the specificity profile obtained by Choe *et al.*;^8^ however, use of the HyCoSuL approach allowed us to obtain a highly detailed picture of cathepsin L active site preferences. We found that cathepsin L has a relatively narrow specificity in the S2 pocket, in agreement with proteomic studies.^26^,^27^,^59^ Moreover, unnatural Phe derivatives were well-recognized (the best being Phe(3-Cl)). Other amino acids were only poorly recognized or ignored by cathepsin L. Based on the P2 specificity matrix, we conclude that the cathepsin L S2 pocket is hydrophobic but not large, as some Phe analogs (for example, hPhe or 1-Nal) were very weakly hydrolyzed. The P3 position appeared more tolerant than the P2 position. Cathepsin L mostly preferred basic (Arg, Lys, Orn and Dab) and some hydrophobic (Phg and Nle(OBzl)) amino acids. Interestingly, some d-amino acids were better recognized by cathepsin L than their l-enantiomers, indicating that the S3 pocket is not stereochemically specific. Moreover, several amino acids (mostly Phe derivatives) were completely ignored by cathepsin L. Similarly to P3, P4 also displayed a broad specificity with preference for basic amino acids (Dab, Dap, Orn and Agp) and the ability to tolerate d-amino acids. Among natural amino acids, only branched Leu, Ile and Val and hydrophobic Phe and Tyr were very poorly recognized by cathepsin L. Among unnatural amino acids, only Phe derivatives were not recognized.

Finally, HyCoSuL screening represents a valuable tool in the design of potent or selective enzyme substrates; however, we note that HyCoSuL (like PS-SCL^8^,^60^) is a combinatorial technique, so the potential cooperativity of protease subsites must be considered. To rule out this possibility in terms of cathepsin L, we synthesized three sets of substrates with the formulas Ac-P4-Lys-Phe-Arg-ACC, Ac-His-P3-Phe-Arg-ACC and Ac-His-Lys-P2-Arg-ACC and performed kinetic studies. When we compared their *k*_cat_/*K*_M_ profiles to the HyCoSuL screening data, we found that cathepsin L displays almost no S4–S2 subsite cooperativity (Fig. S1†).

### 3.3. P1 individual substrate library

**Fig. 3:**
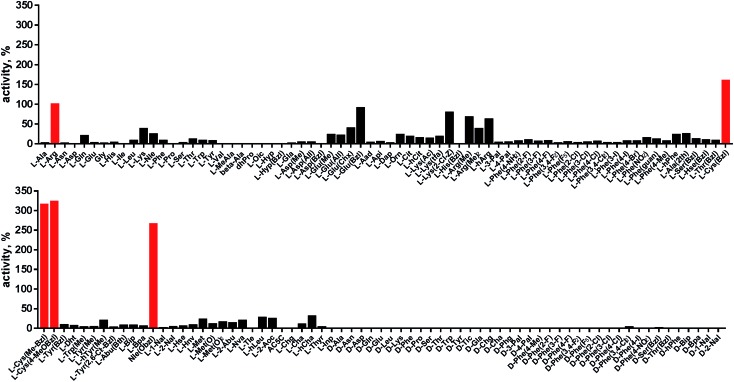
Human cathepsin L preferences at the P1 position. P1 human cathepsin L preferences were determined using an individual fluorogenic substrate library with the general formula Ac-Ala-Arg-Leu-P1-ACC. In this library, the P1 position is occupied by either a natural or an unnatural amino acid. The *x* axis shows the abbreviated names of the amino acids and the *y* axis displays the relative activity presented as a percentage of the best-recognized amino acid. The standard deviations calculated from three screenings were 15% of the values shown in the figure. The red bars indicate the best natural (Arg) or unnatural amino acids.

Cathepsin L reportedly cleaves substrates at the carboxylic site of Arg (P1 position); however, other amino acids are tolerated by this protease in the S1 pocket. Choe *et al.* demonstrated that at P1 cathepsin L also recognizes Lys, Gln, Thr, Nle and Met,^8^ in agreement with proteomic data.^26^,^59^ To gain further insight into the P1 preferences of cathepsin L we utilized a 139-membered individual substrate library containing over 100 unnatural amino acids. As a P4–P2 scaffold we selected an Ala-Arg-Leu sequence that is recognized by almost all cysteine cathepsins.^8^ The Ac-Ala-Arg-Leu-P1-ACC library contains diverse chemical groups at P1: basic, acidic, hydrophilic, hydrophobic and linear, among others. The specificity matrix is presented in Fig. 3 (the reference Arg and the best amino acids are shown in red). Our analysis revealed that some unnatural amino acids, namely Cys(Bzl), Cys(MeBzl), Cys(MeOBzl) and Nle(OBzl), are significantly better recognized at P1 than the natural residue Arg. This observation demonstrates that the cathepsin L S1 pocket has dual properties: it is negatively charged as it accepts Arg and hArg, but it can also accommodate large, hydrophobic amino acid side chains.

### 3.4. Development of novel, potent cathepsin L substrates

**Fig. 4:**
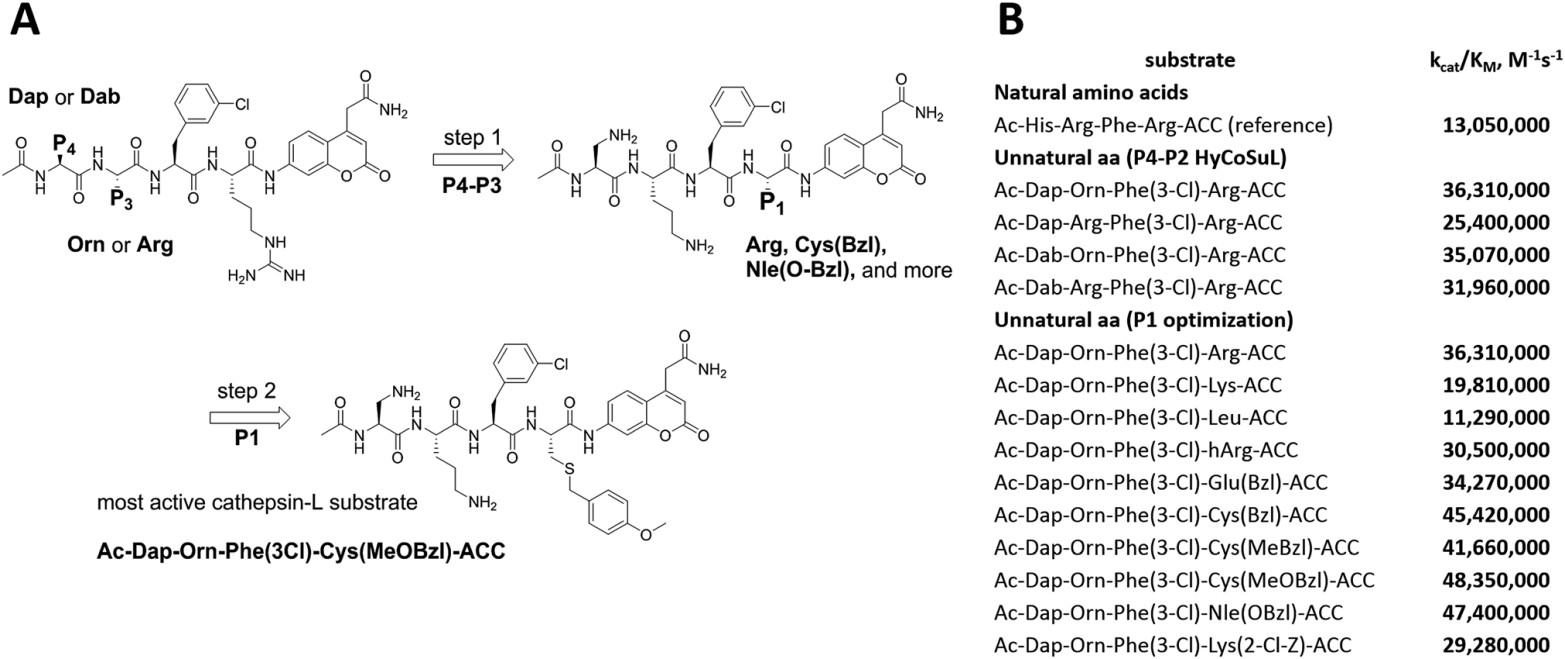
New, potent cathepsin L substrates. Panel A: schematic representation of the two-step procedure used to develop novel, active cathepsin L substrates. The protocol combines the HyCoSuL approach with use of the P1 individual substrate library. Using the former, we extracted the Dap-Orn-Phe(3-Cl) motif as the optimal motif in the P4–P2 positions. Then we added the best amino acids from the P1 library screening to obtain the Ac-Dap-Orn-Phe(3-Cl)-Cys(OMeBzl)-ACC substrate. Panel B: the kinetic parameters (*k*_cat_/*K*_M_) for the most active cathepsin L substrates developed through combining HyCoSuL profiling with P1-optimization; aa – amino acids.

To develop active cathepsin L substrates we combined the HyCoSuL approach with the results from the P1 library screening (Fig. 4). Initially, we synthesized the four potentially best substrates with various P4–P2 regions (Table S1†) and compared the hydrolysis efficiency with the reference Ac-His-Arg-Phe-Arg-ACC substrate (Fig. 4). Kinetic analysis revealed the best cathepsin L substrate (with Arg fixed in P1) to be Ac-Dap-Orn-Phe(3-Cl)-Arg-ACC, which was recognized almost three times better (*k*_cat_/*K*_M_) than the reference substrate. Next, we synthesized a 10-membered library of substrates in which the P4–P2 region was fixed with the best recognition sequence, while the P1 position was varied (Fig. 4 and Tables S1 and S2†). *k*_cat_/*K*_M_ analysis allowed us to synthesize two highly active substrates, Ac-Dap-Orn-Phe(3-Cl)-Cys(MeOBzl)-ACC and Ac-Dap-Orn-Phe(3-Cl)-Nle(OBzl)-ACC, which are the most potent cathepsin L tetrapeptide substrates reported to date: both are hydrolyzed by cathepsin L almost 4-times faster than the Ac-His-Arg-Phe-Arg-ACC substrate is.

### 3.5. Analysis of cathepsin L-selective substrates

**Fig. 5:**
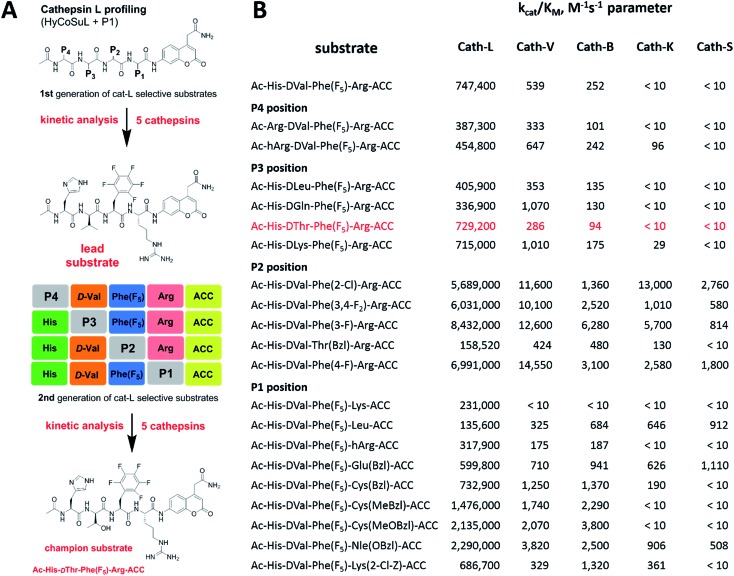
Analysis of cathepsin L-selective substrates. Panel A: outline of the two-step procedure used to develop cathepsin L-selective substrates. Initially, HyCoSuL and P1-library screening data is used to select P4–P1 cathepsin L-characteristic amino acids, which are then selected to synthesize the first generation set of substrates. After kinetic analysis of five cathepsins, the lead substrate is selected and used for second generation substrate synthesis. The second round of kinetic analysis reveals highly specific cathepsin L substrates. Panel B: kinetic analysis (*k*_cat_/*K*_M_) of defined second-generation cathepsin L substrates toward five cysteine cathepsins. Experiments were carried out in triplicate and the standard deviations were below 15%.

Although the family of Ac-Dap-Orn-Phe(3-Cl)-**P1**-ACC substrates are very potent cathepsin L substrates, they display only moderate or even poor selectivity towards other cysteine cathepsins (data not shown). Next, to develop a highly selective cathepsin L substrate, we synthesized eleven tetrapeptide substrates with various P4–P1 sequences (Fig. 5, Table S3†). For each position we selected the most characteristic amino acids from the cathepsin L P4–P2 profile (Fig. 2). For the P4 position we selected a variety of amino acid side chains: Tic, His, Ala and Met(O). In P3 we chose the best amino acids (Orn and Dab) and several d-amino acids, which are also well tolerated. P2 was occupied by Phe derivatives, the group preferred by the enzyme. For P1, we selected mostly basic amino acids (Arg, Orn and Lys). We then hydrolyzed these first-generation substrates (100 μM each) with 5 nM of cysteine cathepsins B, K, L, S and V (Table S4†) and utilized the most selective substrate (Ac-His-dVal-Phe(F_5_)-Arg-ACC) as a candidate for further structure optimization. We next synthesized a second-generation library of substrates, replacing one position at a time (from P4 to P1) (Fig. 5 and Tables S5 and S6†). For P4 we selected His, Arg and hArg; for P3, several d-amino acids; for P2, Phe analogues; and for P1, 10 amino acids best recognized by cathepsin L (Ac-Ala-Arg-Leu-P1-ACC library profiling, Fig. 3). *k*_cat_/*K*_M_ profiling of these substrates with L, V, B, K and S cathepsins revealed several “cathepsin L selectivity requirements” (Fig. 5). For P4, the most selective amino acid appeared to be His, as this substrate displayed a higher *k*_cat_/*K*_M_ value towards cathepsin L than the P4 Arg derivatives, while maintaining similar catalytic parameters for the other four cathepsins. P3 was occupied by four different d-amino acids, among which the d-Thr displayed the highest affinity for cathepsin L and only residual activity in the presence of other enzymes (*k*_cat_/*K*_M_ values of 286 M^–1^ s^–1^ for cathepsin V and 94 M^–1^ s^–1^ for cathepsin B). Moreover, P2 was critical in terms of discriminating cathepsin L from other cysteine cathepsins. Mono- and di-substituted Phe are more active than 2,3,4,5,6-pentafluorophenylalanine (Phe(F_5_)) in the P2 position on cathepsin L, however, Phe(F_5_) is poorly recognized by other cathepsins, rendering Phe(F_5_) in the P2 position the most selective amino acid for cathepsin L.

Analysis of P1 specificity showed that various amino acids can be used to construct cathepsin L-selective substrates. Overall, the kinetic analysis performed on the second-generation cathepsin L substrates revealed that the most selective substrate from this library was Ac-His-dThr-Phe(F_5_)-Arg-ACC, which displayed almost non-detectable hydrolysis when tested with other cysteine cathepsins (Fig. 5). Moreover, a detailed kinetic analysis of two cathepsin L selective substrates revealed that discrimination between cathepsin L and other cathepsins is mainly driven by *k*_cat_, whereas the *K*_M_ parameter has almost no impact on selectivity (Table S7†).

### 3.6. Design and kinetic analysis of cathepsin ABPs

**Fig. 6:**
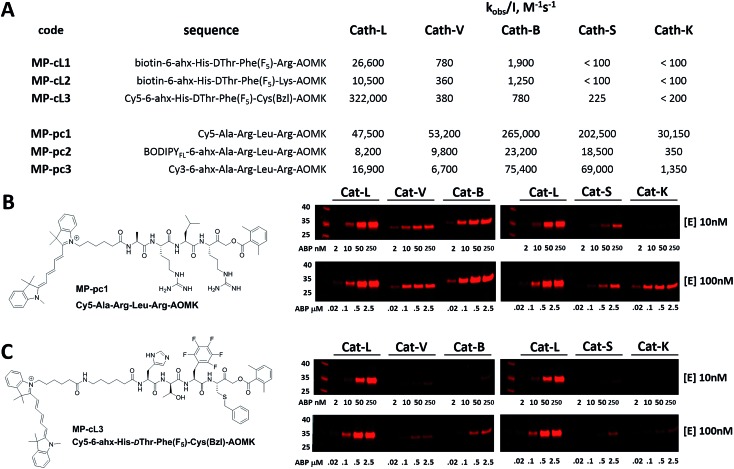
Broad spectrum and cathepsin L selective ABPs. Panel A: kinetic parameters (*k*_obs_/*I*, M^–1^ s^–1^) of six ABPs assessed in the presence of five recombinant human cathepsins. Experiments were carried out in triplicate and the standard deviations were below 20%. Panels B and C show labeling of recombinant cathepsins using two Cy5-labeled ABPs: a pan-cathepsin specific MP-pc1 probe (Panel B) and the cathepsin L-specific MP-cL3 probe (Panel C). The enzymes (10 nM or 100 nM) were incubated separately with various probe concentrations for 30 min and then subject to SDS-PAGE analysis. The fluorescence was scanned using the 700 nm channel (LI-COR). In both experiments (enzymes at 10 nM or 100 nM) the probe/enzyme ratio was kept constant at 0.2, 1, 5 and 10.

We next designed probes to track cathepsin L activity in cells by synthesizing two biotin-labeled ABPs containing the most selective cathepsin L sequence (MP-cL1 and MP-cL2, Table S8†). Both were equipped with an electrophilic AOMK warhead. Kinetic analysis revealed that although the probes are selective for cathepsin L over V, S and K, they slightly cross-react with cathepsin B (Fig. 6). Changing the biotin tag to cyanine-5 and the P1 Arg to Cys(Bzl) significantly improved probe activity and selectivity (MP-cL3, Fig. 6 and Table S8†). We then compared compound specificity with generic (pan-specific) cathepsin probes containing the Ala-Arg-Leu-Arg sequence by synthesizing three fluorescently-labeled probes and measuring their *k*_obs_/*I* values in the presence of recombinant cathepsins (MP-pc1, MP-pc2 and MP-pc3, Table S8†). All were broad spectrum probes (Fig. 6), however, the MP-pc1 probe displayed the highest activity for all five enzymes and was thus further analyzed as a representative pan-specific cathepsin probe. Interestingly, we observed a significant difference in potency between Cy3- and Cy5-labeled probes (Fig. 6). Since these tags have almost identical structures, we conclude that the differences are caused by the 6-ahx spacer, which may be reducing the probe’s potency.

### 3.7. Detection of cathepsin L in cells using biotinylated probes

Biotin-tagged ABPs currently represent the gold standard in terms of biological application and have been successfully used to isolate labelled proteins by affinity enrichment/purification on appropriate beads.^29^,^61^,^62^ Previous *in vitro* kinetic assays with recombinant cathepsins show that MP-cL1 and MP-cL2 probes are potent for the detection of cathepsin L and display only minor off-target activity against other cathepsins (mainly cathepsin B) (Fig. 6). Thus we wanted to validate probe utility in living cells. Given that the biotin tag can significantly reduce the permeability of an ABP, we first investigated probe efficiency in human embryonic kidney (HEK293T) cathepsin L-transfected cells. After incubation of these probes in the 0–10 μM range with cells, we observed a moderately strong signal in the cathepsin MW range at probe concentrations as low as 100 nM (Fig. S2,† Panel A). The signal increased when the probes were used at 1 μM and 10 μM, respectively. Moreover, there was no cathepsin labelling when cells were preincubated with 25 μM of E-64d, a cell-permeable, broad spectrum cathepsin inhibitor. Next, to assess applications of these two probes in biological studies, we tested their ability to interact with cathepsin L as a potential means to identify active proteases. To do so, we performed a pulldown assay using streptavidin-coated magnetic beads and observed strong signals corresponding to cathepsin L in the eluting fractions, and these signals were completely abolished following use of the E-64d inhibitor (Fig. S2B†). Furthermore, to estimate cathepsin L/B cross-reactivity in HEK293T cells overexpressing cathepsin L, we performed a pulldown assay on streptavidin magnetic beads and then performed immunoblotting of cathepsin L and B in the eluting fractions. To do so, we incubated HEK293T cells overexpressing cathepsin L with both probes, prepared cell lysates, and then evaluated the enrichment of biotinylated proteins using streptavidin magnetic beads. We found that both MP-cL1 and MP-cL2 probes could be used to enrich cathepsin L, based on the appearance of distinct bands in the cathepsin MW range (Fig. S2C†). Strong bands corresponding to cathepsin L were seen in the eluting fractions (Fig. S2C†), but only a weak band corresponding to cathepsin B was detected in those fractions (Fig. S2D†). Based on the signal intensity, we observed only minor cross-reactivity of these probes with cathepsin B even after prolonged incubation, in agreement with the kinetic data (Fig. 6).

### 3.8. Cathepsin labeling by two Cy5 ABPs

Since biotin-labeled probes are not suitable for direct visualization of protease activity, we utilized the cathepsin L selective sequence (His-dThr-Phe(F_5_)) to construct a fluorescently-tagged probe (MP-cL3). To confirm probe specificity (Fig. 6) and assess the activity of the MP-pc1 probe, we performed *in vitro* labeling using five human cathepsins (L, V, B, S and K). Both probes were incubated separately at various concentrations with active site-titrated cathepsins (10 nM or 100 nM) for 30 min, and SDS-PAGE analysis was then carried out (Fig. 6). In both experiments, regardless of the cathepsin concentration, the probe/enzyme ratio was kept constant at 0.2, 1, 5 and 25. This analysis revealed that the broad spectrum MP-pc1 probe can label cathepsins L, V, B and S (10 nM) even at a 1 : 1 ratio. When cathepsins were used at the higher concentration of 100 nM this probe also labels cathepsin K. Importantly, the MP-cL3 probe displayed a very high degree of selectivity, regardless of the cathepsin concentration used (Fig. 6). Cathepsin V, the closest cathepsin L homolog, and cathepsin B are slightly labeled and only at high probe/enzyme concentrations, demonstrating that the HyCoSuL screening data can be directly applied to ABP design.

### 3.9. Detection and selective labeling of cathepsin L in cells

Our ultimate goal is to develop a chemical tool capable of selectively labeling active cathepsin L in cells. This task is challenging since cathepsin B is highly expressed in numerous cells (including cancer cells) and cathepsins B and L share some substrate preferences. Our initial analyses indicate that cathepsin L can be distinguished from cathepsin B (Fig. 6). To validate these results in a more biological setting we tested Cy5-labeled MP-pc1 and MP-cL3 using HEK293T cells, which reportedly express higher levels of cathepsin L than B.^56^ To do so we incubated HEK293T cells with each probe for 0–18 hours, and in parallel we performed immunoblotting analysis. Both probes could label active cathepsins in living cells, as they were taken up into cellular lysosome/endosome compartments within an hour of incubation (Fig. S3†). Moreover, the pan-specific MP-pc1 probe labeled both cathepsins L (25 kDa and 30 kDa) and B (30 kDa), while the HyCoSuL-derived selective MP-cL3 probe bound only cathepsin L, even after prolonged (18 hour) incubation. Interestingly, analysis using a cathepsin L antibody shows that the major form of cathepsin L in HEK293T cells is the heavy chain (25 kDa) form, which was the primary form labeled by the MP-cL3 probe (Fig. S3†). The single chain (30 kDa) form, which also displays catalytic activity, was also labeled by the probe (Fig. S3†).

**Fig. 7:**
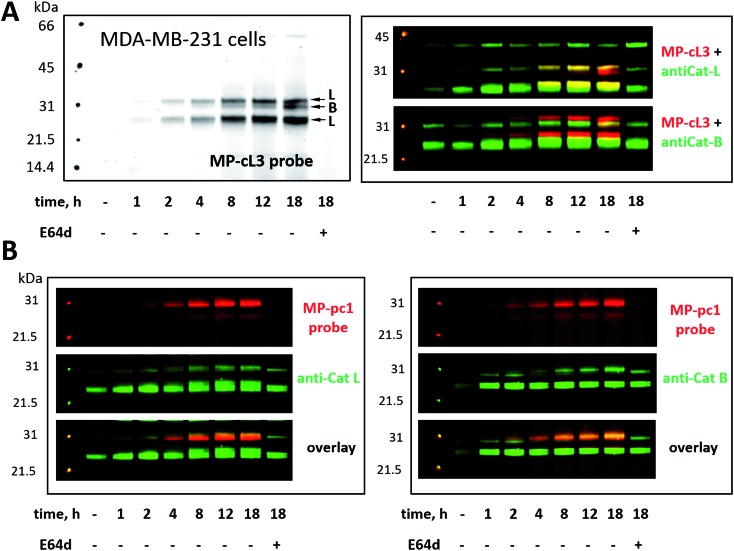
Cathepsin labeling in MDA-MB-231 cells using Cy5-labeled ABPs. Panel A: MP-cL3 begins to label active cathepsin L in living cells after 1 hour and remains selective for cathepsin L up to 8 hours (when the fluorescence signal becomes almost saturated). After 8 hours the MP-cL3 probe also labels cathepsin B, however, even after 18 hours this labeling is poor. As indicated by antibody analysis, the MP-cL3 probe labels both the single chain (30 kDa) and heavy chain (25 kDa) forms of cathepsin L. Pre-incubation of cells with 25 μM of E64d blocked all cysteine cathepsin activity (Panel A, last lane). Panel B: the generic cathepsin probe, MP-pc1, labels mostly cathepsin B, as indicated by antibody analysis.

We next challenged the selectivity of the MP-cL3 probe in MDA-MB-231 cells, similarly to in HEK293T cells, since these cells contain more cathepsin B. We found that MP-cL3 is rapidly taken up by cells, is detectable after 1 hour and demonstrated no labeling of cathepsin B up to 8 hours (Fig. 7 and S4†). Thereafter, the fluorescence signal appeared saturated, suggesting that 8 hours is sufficient to inhibit (and detect) cathepsin L activity. Prolonged (18 hour) incubation of the probe with cells resulted in non-specific but faint labeling of cathepsin B (Fig. 7A). As all three bands were assigned to cathepsin L, or cathepsin B, this analysis also demonstrated that the MP-cL3 probe does not label cathepsin V (L2), a close cathepsin L homolog. Interestingly, MDA-MB-231 cells may process cathepsin L differently than HEK293T cells, as the former do not display clear differences in the levels of cathepsin L single and heavy chains. Moreover, as expected, the broad spectrum MP-pc1 probe bound mostly to cathepsin B, since cathepsin L labeling was almost negligible (Fig. 7B).

### 3.10. Detection of active cathepsin L by fluorescence microscopy

**Fig. 8:**
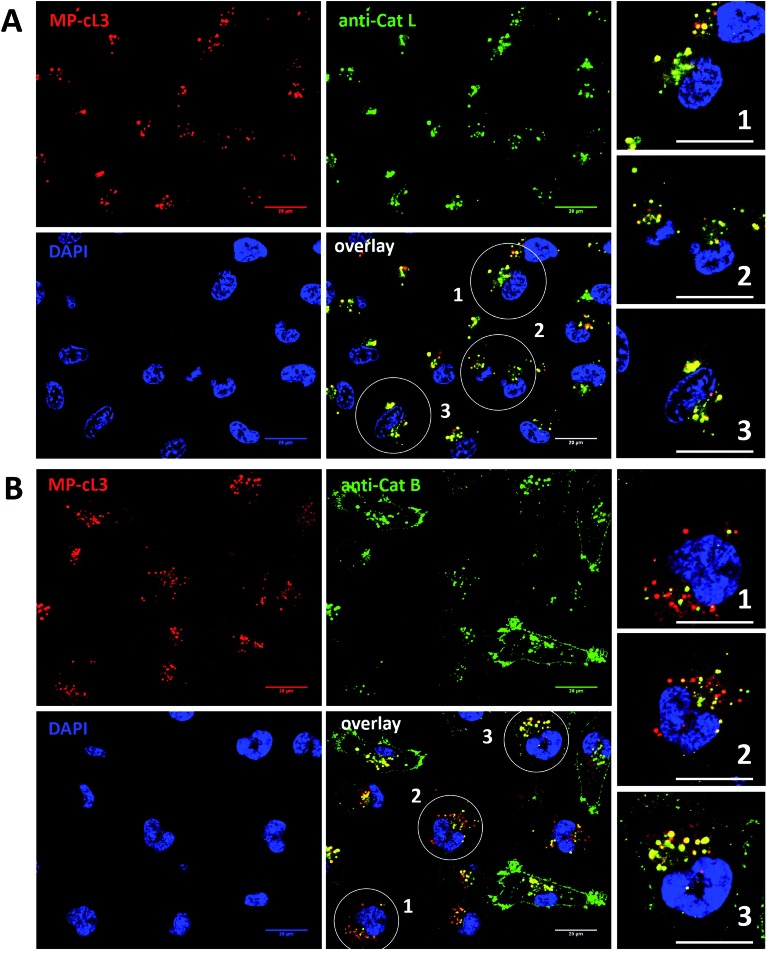
Cathepsin L labeling of MDA-MB-231 cells. Panel A: the cathepsin L selective probe, MP-cL3 (1 μM), was incubated with MDA-MB-231 cells for 8 hours and the cells were then subjected to confocal fluorescence microscopy. The cathepsin L antibody detects both the proenzyme and the active enzyme, however, Cy5 detects only active cathepsin L. In the overlay, green spots indicate the inactive enzyme, as yellow show only active cathepsin L. The lack of red spots in the overlay image strongly indicates the high selectivity of the MP-cL3 probe. Panel B: the same experiment as in Panel A, but here localization of the MP-cL3 probe was paired with staining with a cathepsin B antibody. The results demonstrate that cathepsin B is not distributed equally with cathepsin L, as there are lysosomes/endosomes in which both enzymes are active, and others in which only active cathepsin B is present. Both experiments (panels A and B) were performed three times and representative pictures are presented. Scale bar: 20 μm.

Our data indicate that we could achieve high selectivity for cathepsin L by incubating MP-cL3 in cells for up to 8 hours (Fig. 7A). We utilized this information to visualize cathepsin L activity in MDA-MB-231 cells using confocal fluorescence microscopy. We incubated 1 μM of the MP-cL3 probe with MDA-MB-231 cells for 8 hours, and then methanol-fixed cells were labeled with either cathepsin L or cathepsin B antibodies for imaging (Fig. 8 and S5–S8†). Since the MP-cL3 probe is not a quenched probe (qABP), it has to be stressed here that all non-bound probe must be washed from fixed cells prior to imaging in order to avoid false-positive signals. We found that the probe signals (red) greatly overlapped with those of cathepsin L antibodies (green), and importantly, the MP-cL3 probe labeled only active cathepsin L (Fig. 8A, S5 and S6†). Our western blot analysis demonstrates that there is also a portion of inactive (pro)cathepsin L, however, we could not detect this using fluorescence microscopy as it seems that active and inactive cathepsin L share the same localization (Fig. S10†). More crucially, we detected very few red spots alone in the overlay image, strongly suggesting that there is negligible off-target binding by the MP-cL3 probe. In parallel experiments, we stained cells with a cathepsin B antibody and merged the image with the MP-cL3 probe localization (Fig. 8B, S7 and S8†). The presence of overlapping signals strongly suggests that cathepsins L and B reside in the same lysosomes/endosomes, however, we observed some organelles in which only cathepsin B, but not cathepsin L, is active (Fig. 8B, S7 and S8†). Additional analysis confirmed that prolonged incubation of the MP-cL3 probe with MDA-MB-231 cells (24 hours) results in additional labeling, likely due to other cathepsins, confirming that the MP-cL3 probe becomes non-selective after 8 hours (Fig. S9 and S10†). Thus, the probe concentration and the time of incubation with cells are both critical to achieve selectivity in cells. Finally, we also incubated MDA-MB-231 cells with the broad spectrum MP-pc1 probe (1 μM, 8 hours) and found that it primarily labeled cathepsin B, since the degree of probe/enzyme overlap was higher for cathepsin B than for the cathepsin L enzyme (Fig. S11†).

### 3.11. Colocalization between cathepsin L, cathepsin B and the MP-cL3 ABP

**Fig. 9:**
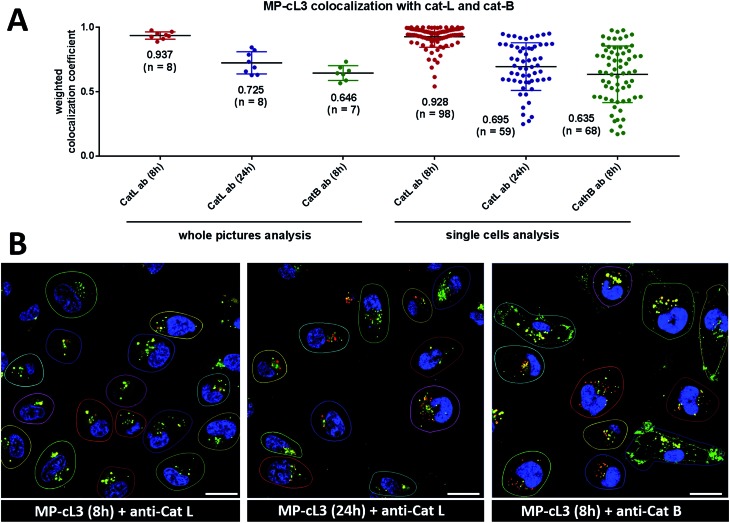
Colocalization of the MP-cL3 probe with cathepsin L and cathepsin B in MDA-MB-231 cancer cells. Panel A: the weighted colocalization coefficients calculated for ABP/cat-L and ABP/cat-B based on (1) whole picture analysis or (2) single cell analysis. The points on the graph indicate single pictures (left) or single cells (right) with “*n*” defining the number of pictures/single cells taken for analysis. The numbers in brackets on the *x*-axis indicate the incubation time of MP-cL3 with the MDA-MB-231 cells. Panel B: the representative images used for the calculation of the weighted colocalization coefficients between the MP-cL3 probe and the cathepsin L (or B) antibody. Red is the ABP, green is the cathepsin antibody and blue is DAPI. The scale bar is 20 μm.

To investigate the colocalization of MP-cL3 with the cathepsin L protein in MDA-MB-231 cells we performed a quantitative pixel-by-pixel analysis of multiple fluorescence microscopy images. A reliable parameter for such analysis is a weighted colocalization coefficient (wcc), which provides information not only about single pixels overlapping, but also correlates their intensities, making the analysis more accurate.^63^,^64^ For this analysis we used: (1) the MP-cL3 probe (selective labeling – 8 hours) + anti-cathepsin L (8 images, 98 single cells analyzed), (2) the MP-cL3 probe (non-selective labeling – 24 hours) + anti-cathepsin L (8 images, 59 single cells analyzed) and (3) the MP-cL3 probe (selective labeling – 8 hours) + anti-cathepsin B (7 images, 68 single cells analyzed) (Fig. 9 and S12†). We demonstrated that the highest weighted colocalization coefficient was obtained when the MP-cL3 probe was incubated with MDA-MB-231 cells for 8 hours (wwc = 0.937 and 0.928) (Fig. S5†). Prolonged incubation of this probe with cells (24 hours) resulted in a decline of this parameter (wwc = 0.725 and 0.695) (Fig. S9†). Colocalization of the MP-cL3 probe with the cathepsin B antibody resulted in the lowest wwc value (0.646 and 0.635) (Fig. S7†). To further demonstrate the usefulness of the wwc parameter we constructed histograms from randomly selected lines from the microscope images and overlaid the probe signal (red) with either the cathepsin L or cathepsin B antibody signal (green) (Fig. S6 and S8†). The histograms for the cathepsin L antibody (green) and the MP-cL3 probe (red) overlapped very well (Fig. S6B†), whereas the histograms for the cathepsin B antibody and MP-cL3 matched poorly (Fig. S8B†).

## 4. Conclusions

The development of protease-specific substrates offering direct readout (fluorogenic or chromogenic) is of great significance since such tools could be extremely useful in biochemical and biological studies of proteases. Starting from broad spectrum chromogenic substrates developed in the early 1940s and 1950s, the field has progressed through individual internally quenched fluorescence substrate libraries and combinatorial mixture libraries with natural amino acids.^8^,^65^–^68^ However, most of these tools are insufficient to develop selective substrates to distinguish closely-related proteases that display overlapping substrate specificities (such as caspases, matrix metalloproteases or cysteine cathepsins). To address this challenge, we applied HyCoSuL technology,^40^,^50^,^55^ because the use of such diverse chemical structures in a library allowed the discovery of highly selective cathepsin L substrates and probes. Combined analysis of enzyme preferences in the P4–P1 region allowed us to design an extremely selective tetrapeptide substrate (Ac-His-dThr-Phe(F_5_)-Arg-ACC). Notably, given that we found that the selectivity towards cathepsin L lies in the P4–P2 sequence, the Arg at position P1 could be exchanged for non-charged amino acids to (1) make the substrate and inhibitor more active in the presence of cathepsin L and (2) to avoid potential cross-reactivity with the numerous proteases that prefer Arg at P1. We then synthesized three small molecule probes, with MP-cL3 (Cy5-6-ahx-His-dThr-Phe(F_5_)-Cys(Bzl)-AOMK) being the most potent and selective toward cathepsin L. This probe bound exclusively to cathepsin L in an *in vitro* experiment testing five recombinant cysteine cathepsins. To demonstrate that the enzymes were active, we also used a broad spectrum cathepsin probe, MP-pc1 (Cy5-Ala-Arg-Leu-Arg-AOMK). More importantly, we showed that the MP-cL3 probe selectively labels cathepsin L in HEK293T and MDA-MB-231 cell lines, both of which express cathepsins L and B at different levels. The MP-cL3 probe selectivity for cathepsin L over cathepsin B is of special importance, as cathepsin B is overexpressed in many types of cancers and masks cathepsin L proteolytic activity, making its analysis more complex. Finally, using the MP-cL3 probe, we not only showed specific labeling of cathepsin L in cells but performed quantitative pixel-by-pixel localization studies using fluorescence microscopy. That analysis indicated that cathepsin L co-localizes with cathepsin B in MDA-MB-231 cancer cells and that some cathepsin B-rich lysosomes lack cathepsin L. We also prepared two biotinylated cathepsin L probes that have only minor cross-reactivity with cathepsin B and could serve as tools in diverse biological experiments. In summary, we have developed the first selective cathepsin L ABP, which allows accurate detection of cathepsin L activity in cancer cells. Given that this probe does not react with other cathepsins, it has the potential to facilitate the understanding of cathepsin L function and provide new information about the biological framework of cathepsin L-dependent or -related cancer progression.

## Author contributions

M. P., G. S. S. and M. D. designed the research; M. P., W. R., M. V., K. G. and P. K. performed the research and collected data; D. F., K. V., D. T. and B. T. contributed new reagents and enzymes; M. P., G. S. S. and M. D. analysed and interpreted the data and wrote the manuscript; and W. R., M. V., K. G., P. K., D. F., K. V., D. T. and B. T. critically revised the manuscript.

## Conflicts of interest

There are no conflicts to declare.

## Supplementary Material

Supplementary information
