# Supplementary material for: Selective imaging of cathepsin L in breast cancer by fluorescent activity-based probes
Source: Chem Sci. 2018 Jan 16;9(8):2113–29. doi: 10.1039/c7sc04303a (PMC5896380; doi:10.1039/c7sc04303a)
Supplement: Supplementary file 1 [file SC-009-C7SC04303A-s001.pdf]

Electronic Supplementary Material (ESI) for Chemical Science.
This journal is © The Royal Society of Chemistry 2018

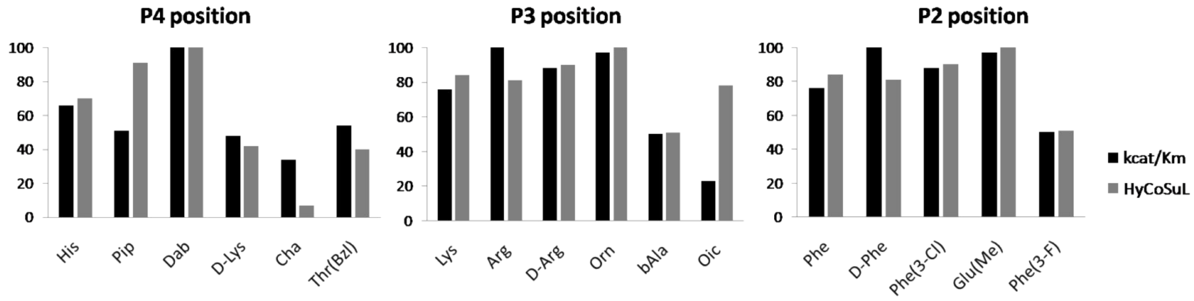

Figure S1 The cathepsin L does not display the subsites cooperativity in S4-S2 pockets. The results from HyCoSuL profiling (substrate combinatorial libraries, grey bars) highly correlate with the results for individual fluorogenic substrates (black bars). The substrate sequences used is this assay were as follows: P4 position Ac-X-Lys-Phe-Arg-ACC, P3 position Ac-His-X-Phe-Arg-ACC, P2 position Ac-His-Lys-X-Arg-ACC. The y axis represents the relative rate of substrates hydrolysis (100% is the substrate with highest activity).

Ac-His-Arg-Phe-Arg-ACC

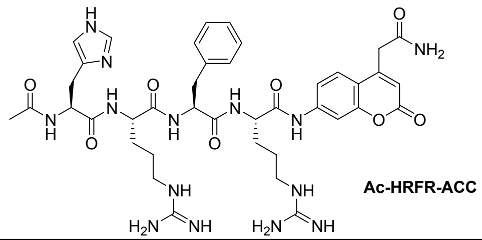

Ac-Dap-Orn-Phe(3-Cl)-Arg-ACC

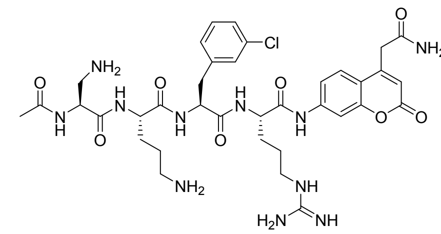

Ac-Dap-Arg-Phe(3-Cl)-Arg-ACC

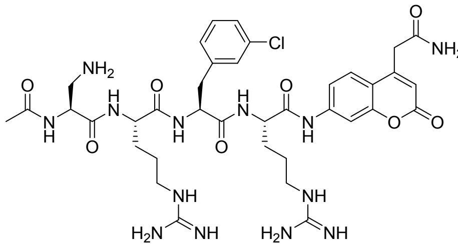

Ac-Dab-Orn-Phe(3-Cl)-Arg-ACC

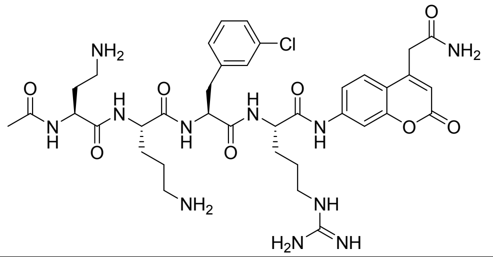

Ac-Dab-Arg-Phe(3-Cl)-Arg-ACC

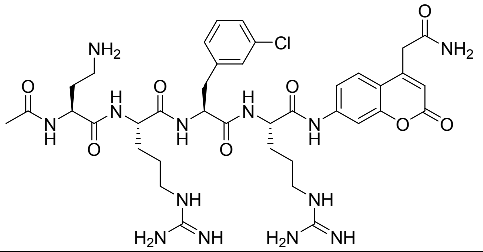

Ac-Dap-Orn-Phe(3-Cl)-Lys-ACC

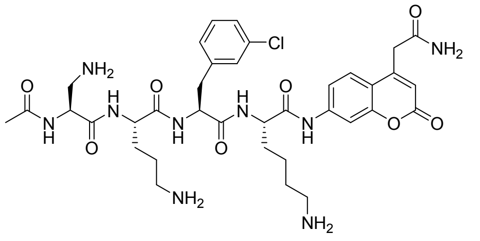

Ac-Dap-Orn-Phe(3-Cl)-hArg-ACC

Ac-Dap-Orn-Phe(3-Cl)-Leu-ACC

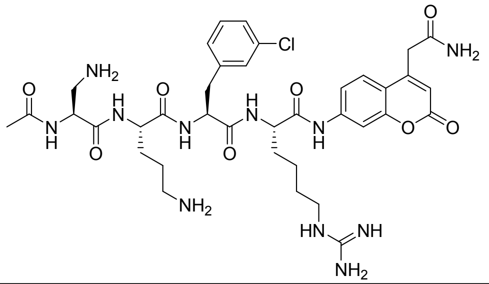

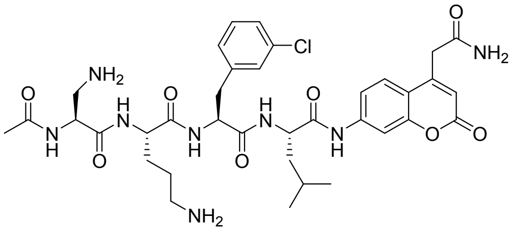

Ac-Dap-Orn-Phe(3-Cl)-Glu(Bzl)-ACC

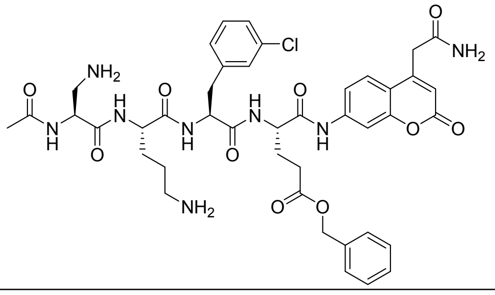

Ac-Dap-Orn-Phe(3-Cl)-Cys(Bzl)-ACC

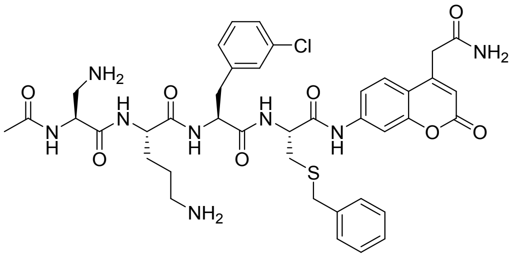

Ac-Dap-Orn-Phe(3-Cl)-Cys(MeBzl)-ACC

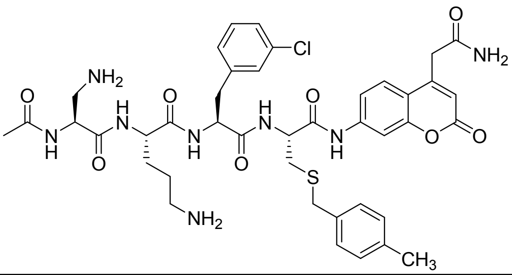

Ac-Dap-Orn-Phe(3-Cl)-Cys(MeOBzl)-ACC

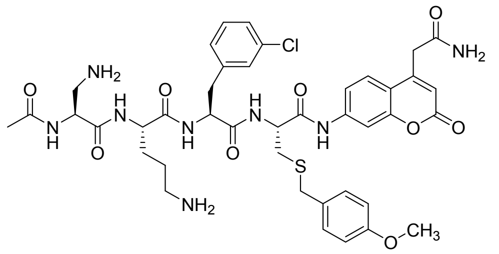

Ac-Dap-Orn-Phe(3-Cl)-Nle(OBzl)-ACC

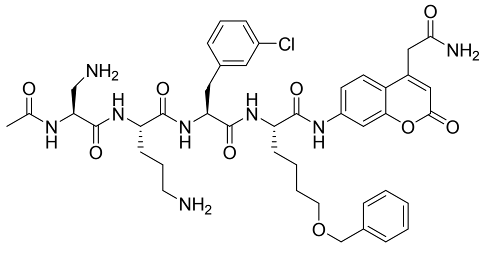

Ac-Dap-Orn-Phe(3-Cl)-Lys(2-Cl-Z)-ACC

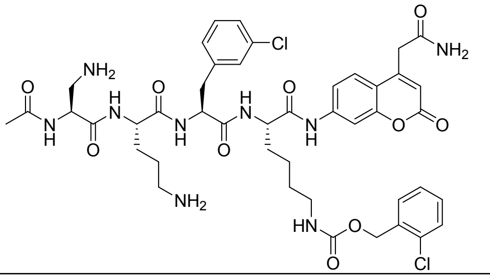

Table S1 The structures of new, very active cathepsin L substrates containing unnatural amino acids. The Ac-His-Arg-Phe-Phe-ACC substrate was used as a reference one.

ACC substrate	K_M, µM	k_{cat}, s^{-1}	k_{cat}/K_M, $M^{-1}s^{-1}$
Ac-Dap-Orn-Phe(3Cl)-Arg-ACC	5.38	195	36,310,000
Ac-Dap-Orn-Phe(3Cl)-hArg-ACC	4.21	123	30,500,000
Ac-Dap-Orn-Phe(3Cl)-Lys-ACC	7.11	141	19,810,000
Ac-Dap-Orn-Phe(3Cl)-Leu-ACC	3.70	41.8	11,290,000
Ac-Dap-Orn-Phe(3Cl)-Glu(Bzl)-ACC	5.67	188	34,270,000
Ac-Dap-Orn-Phe(3Cl)-Cys(Bzl)-ACC	4.28	191	45,420,000
Ac-Dap-Orn-Phe(3Cl)-Cys(MeBzl)-ACC	4.99	207	41,660,000
Ac-Dap-Orn-Phe(3Cl)-Cys(MeOBzl)-ACC	4.15	193	41,660,000
Ac-Dap-Orn-Phe(3Cl)-Nle(O-Bzl)-ACC	2.63	124	47,400,000
Ac-Dap-Orn-Phe(3Cl)-Lys(2-Cl-Z)-ACC	2.98	86.7	29,280,000

Table S2 Detailed kinetic parameters (K_M, k_{cat}, k_{cat}/K_M) for the most active cathepsin L ACC-labeled tetrapeptide substrates. All parameters were calculated from four independent experiments, and S.D. for all values are below 15%.

Ac-Tic-Orn-Phe(4-F)-Leu-ACC

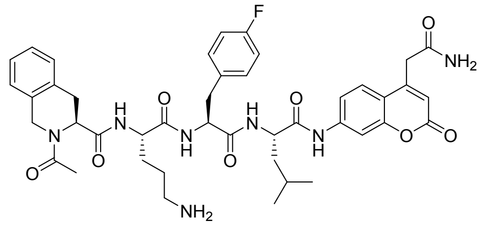

Ac-His-*D*Arg-Phe(3,4-F_2)-Leu-ACC

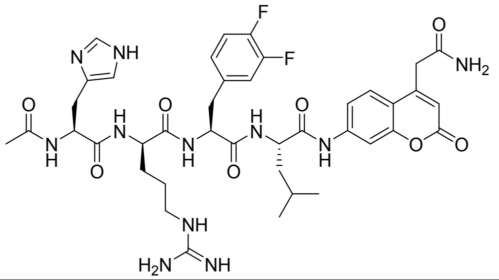

Ac-His-*D*Leu-Phe(F_5)-Orn-ACC

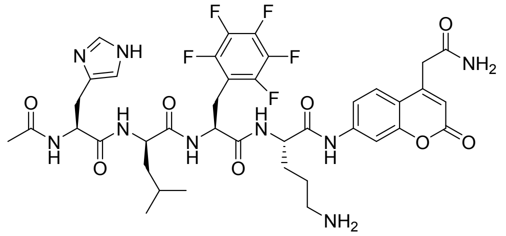

Ac-Tic-*D*Orn-Phe(2-Cl)-Lys-ACC

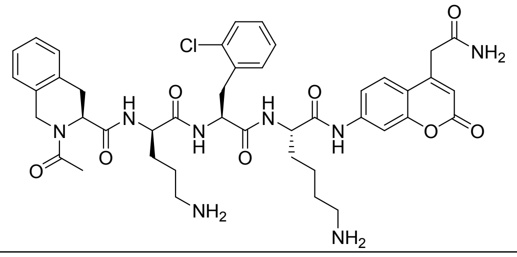

Ac-Met(O)-*D*Arg-Phe(2-F)-Arg-ACC

Ac-Ala-*D*Lys-Lys(Ac)-Arg-ACC

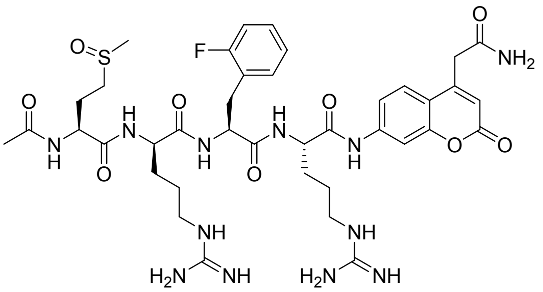

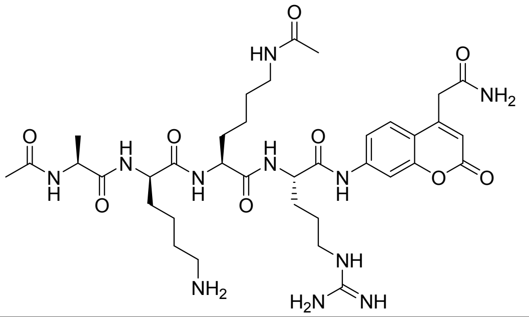

Ac-His-*D*Val-Phe(F5)-Arg-ACC

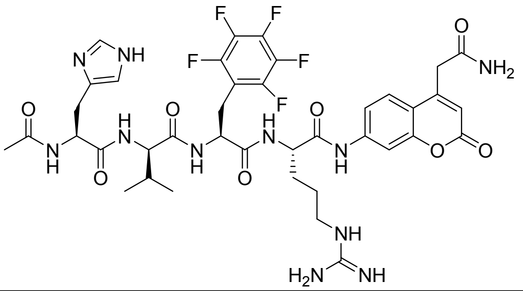

Ac-Tic-Dab-Phe(3,4-F_2)-Arg-ACC

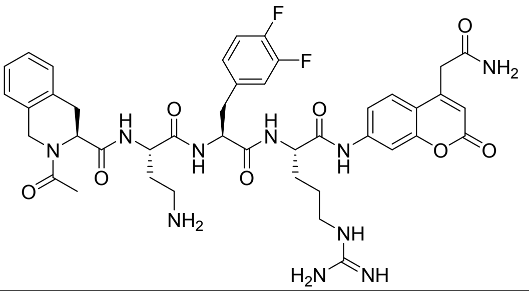

Ac-His-*D*Leu-Phe(3,4-Cl2)-Arg-ACC

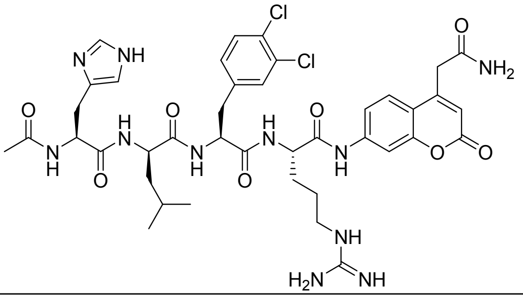

Ac-Ala-Orn-Phe(3,4-Cl_2)-Arg-ACC

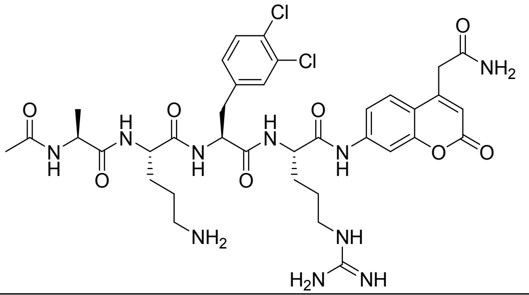

Ac-Pip-*D*Leu-Phe(3,4-F_2)-Arg-ACC

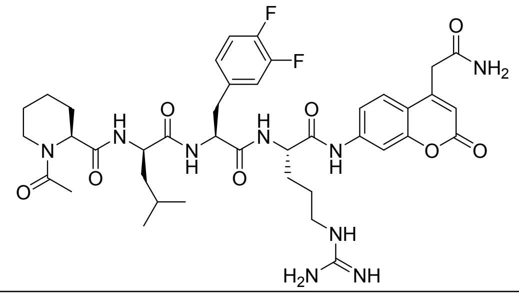

Table S3 The structures of a first generation of cathepsin L selective substrates containing unnatural amino acids.

Substrate Ac-**P4-P3-P2-P1**-ACC	Cathepsin **B**	**K**	**L**	**S**	**V**
	Relative substrate activity RFU/s				
**His-Arg-Phe-Arg**	**272**	**24.5**	**875**	**40.3**	**224**
Tic-Orn-Phe(4-F)-Leu	15.0	0.0	684	4.7	31.7
His-*D*Arg-Phe(3,4-F_2)-Leu	22.3	0.0	474	12.7	30.5
**His-*D*Leu-Phe(F_5)-Orn**	**0.0**	**0.3**	**153**	**0.0**	**0.0**
Tic-*D*Arg-Phe(2-Cl)-Lys	3.1	0.0	134	10.3	36.1
Met(O)-*D*Arg-Phe(2-F)-Arg	76.2	0.2	658	21.1	129
Ala-*D*Lys-Lys(Ac)-Arg	16.9	1.2	25.3	0.0	0.0
**His-*D*Val-Phe(F_5)-Arg**	**0.7**	**1.1**	**706**	**0.0**	**4.4**
Tic-Dab-Phe(3,4-F_2)-Arg	41.2	0.2	825	2.8	28.4
His-*D*Leu-Phe(3,4-Cl_2)-Arg	65.1	1.5	901	4.4	306
Ala-Orn-Phe(3,4-Cl_2)-Arg	189	4.6	844	3.4	346
Pip-*D*Leu-Phe(3,4-F_2)-Arg	9.4	1.2	699	7.8	103

Table S4 The kinetic analysis of a first generation of cathepsin L selective substrate designed based on HyCoSuL profiling. Results in table present the rate of hydrolysis (RFU/s) of each substrate (100μM) by five cysteine cathepsins (B, K, L, S, and V; 5nM). The broad spectrum cathepsins Ac-HRFR-ACC substrate was used as a control. The most specific substrates (selected to construct the second generation library) are bold.

Ac-His-*D*Val-Phe(F_5)-Arg-ACC

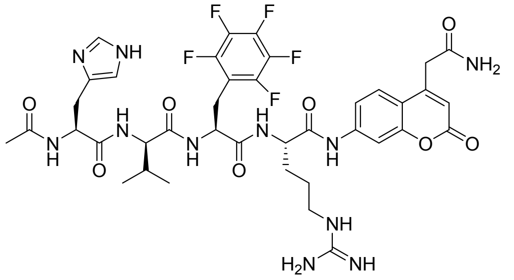

Ac-Arg-*D*Val-Phe(F_5)-Arg-ACC

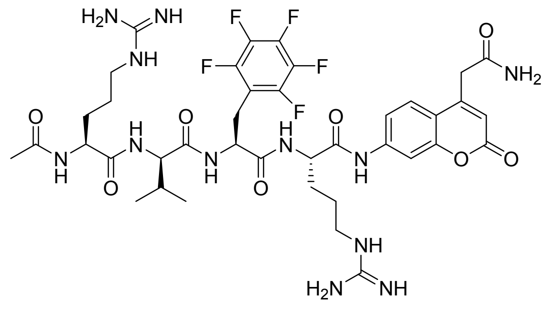

Ac-hArg-*D*Val-Phe(F_5)-Arg-ACC

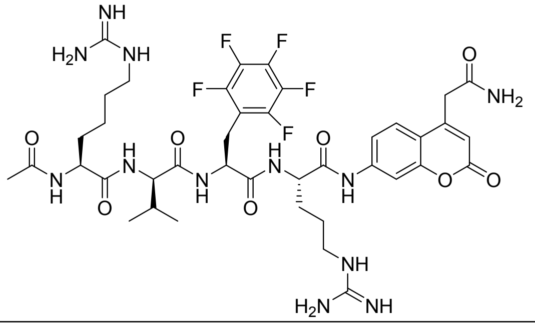

Ac-His-*D*Leu-Phe(F_5)-Arg-ACC

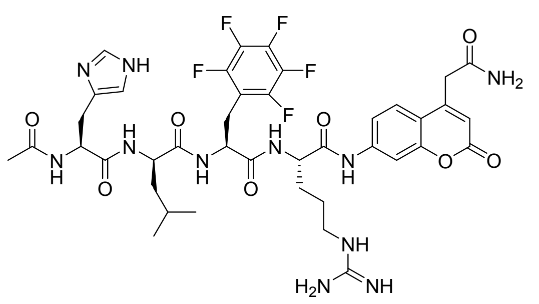

Ac-His-*D*Gln-Phe(F_5)-Arg-ACC

Ac-His-*D*Thr-Phe(F_5)-Arg-ACC

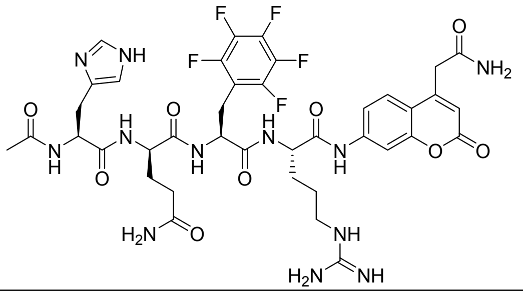

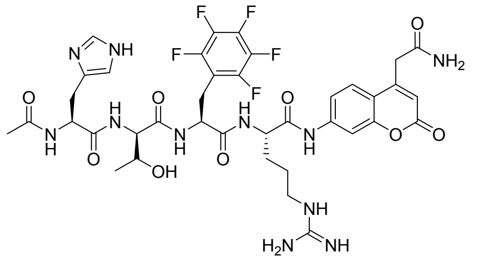

Ac-His-*D*Lys-Phe(F_5)-Arg-ACC

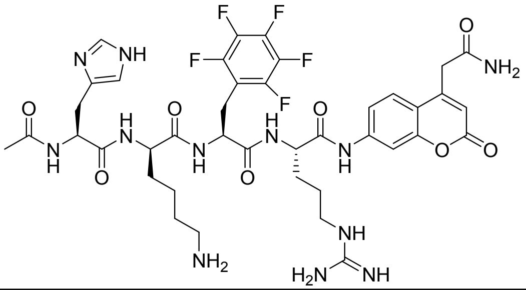

Ac-His-*D*Val-Phe(2-Cl)-Arg-ACC

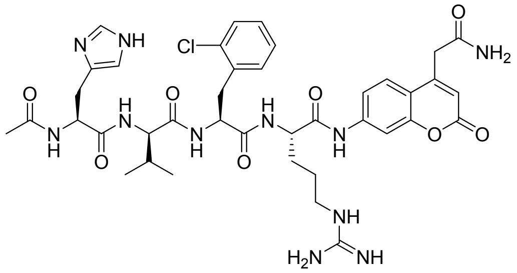

Ac-His-*D*Val-Phe(3,4-F_2)-Arg-ACC

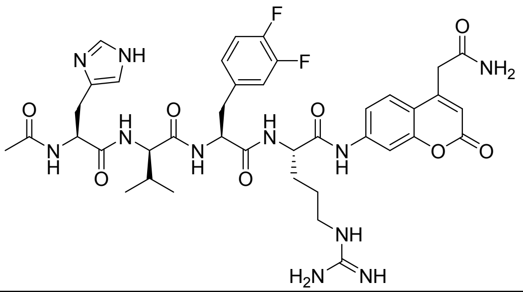

Ac-His-*D*Val-Phe(3-F)-Arg-ACC

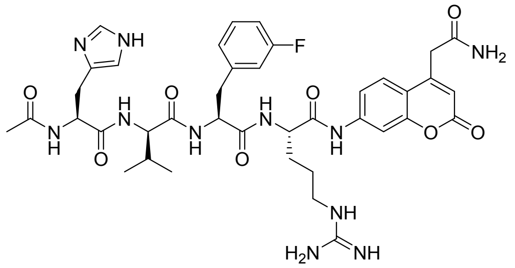

Ac-His-*D*Val-Thr(Bzl)-Arg-ACC

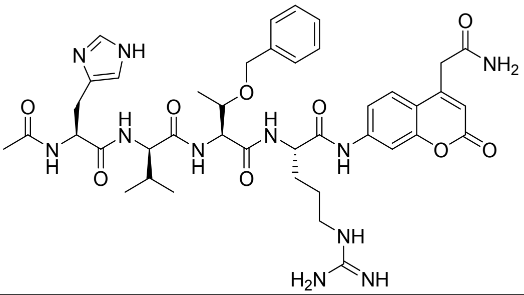

Ac-His-*D*Val-Phe(4-F)-Arg-ACC

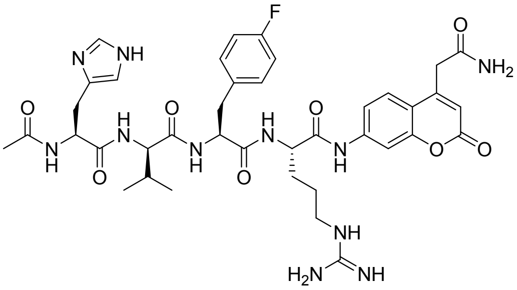

Table S5 The structures of a second generation of cathepsin L specific substrates containing unnatural amino acids.

Ac-His-*D*Thr-Phe(F_5)-**Lys**-ACC

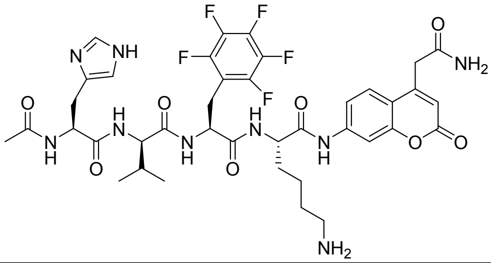

Ac-His-*D*Thr-Phe(F_5)-**Leu**-ACC

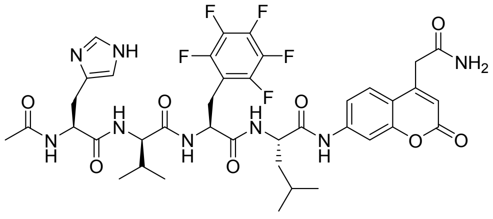

Ac-His-*D*Thr-Phe(F_5)-**hArg**-ACC

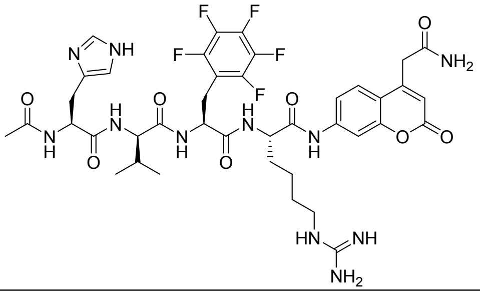

Ac-His-*D*Thr-Phe(F_5)-**Glu(Bzl)**-ACC

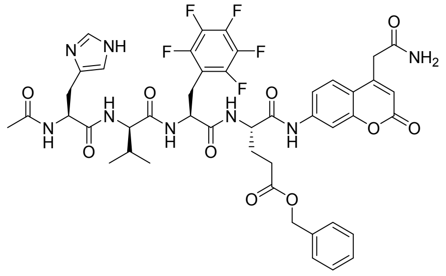

Ac-His-*D*Thr-Phe(F_5)-**Cys(Bzl)**-ACC

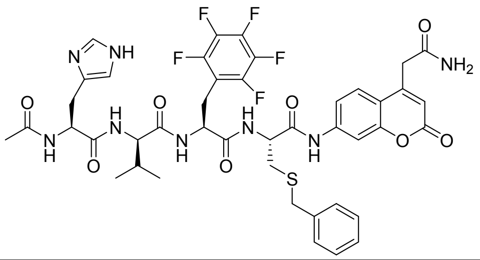

Ac-His-*D*Thr-Phe(F_5)-**Cye(MeBzl)**-ACC

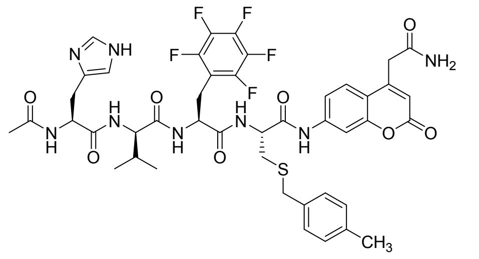

Ac-His-*D*Thr-Phe(F_5)-**Cys(MeOBzl)**-ACC

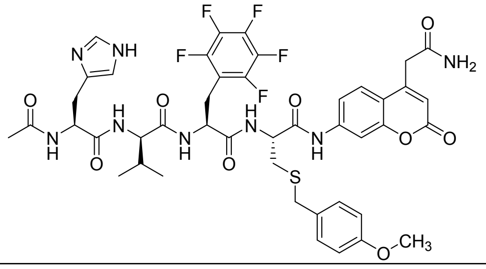

Ac-His-*D*Thr-Phe(F_5)-**Nle(OBzl)**-ACC

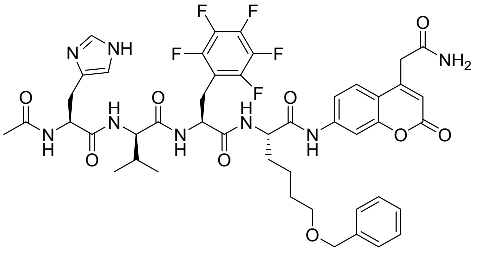

Ac-His-*D*Thr-Phe(F_5)-**Lys(2-Cl-Z)**-ACC

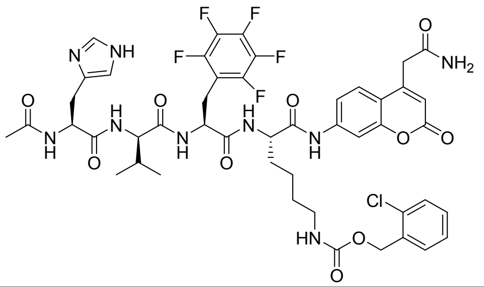

Table S6 Structures of substrates with P4-P2 cathepsin L specific sequence (Ac-**His**-***D*****Thr**-**Phe(F$_5$)**-P1-ACC)

enzyme	k_{cat}/K_M, $M^{-1}s^{-1}$	K_M, μM	k_{cat}, s^{-1}
Substrate: **Ac-His-*D*Thr-Phe(F$_5$)-Arg-ACC**			
Cat L	729,900	53.1	38.6
Cat V	286	61.4	0.0178
Cat B	94	71.5	0.00673
Cat K	< 10	n.d.	n.d.
Cat S	< 10	n.d.	n.d.
Substrate: **Ac-His-*D*Val-Phe(F$_5$)-Cys(Bzl)-ACC**			
Cat L	732,900	5.67	4.21
Cat V	1,250	7.76	0.00971
Cat B	1,370	8.19	0.0115
Cat K	190	11.5	0.00221
Cat S	< 10	n.d.	n.d.

Table S7 Detailed kinetic parameters of two cathepsin L selective ACC substrates. The data demonstrates that the selectivity is mainly driven by k_{cat} factor, whereas K_M parameter has almost no impact on the selectivity. All parameters were calculated from four independent experiments, and S.D. for all values are below 15%. n.d. – not determined.

Cathepsin L selective probes	Pan-specific cathepsin probes
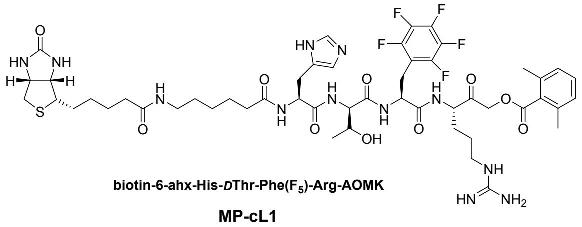 biotin-6-ahx-His-*D*Thr-Phe(F_5)-Arg-AOMK **MP-cL1**	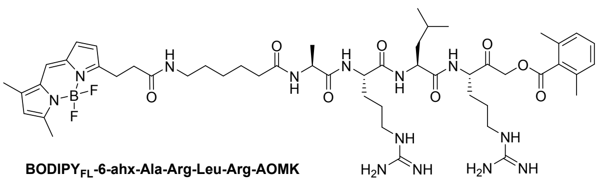 BODIPY$_{FL}$-6-ahx-Ala-Arg-Leu-Arg-AOMK **MP-pc2**
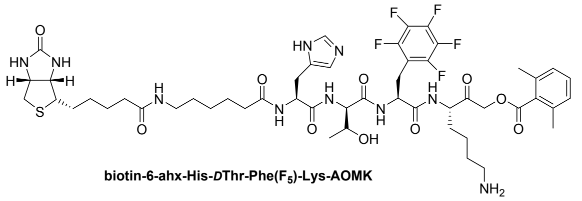 biotin-6-ahx-His-*D*Thr-Phe(F_5)-Lys-AOMK **MP-cL2**	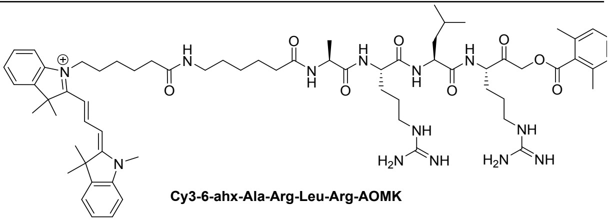 Cy3-6-ahx-Ala-Arg-Leu-Arg-AOMK **MP-pc3**
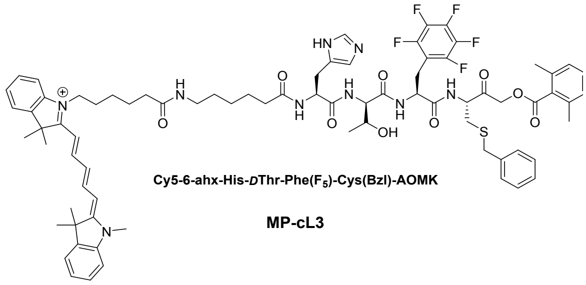 Cy5-6-ahx-His-*D*Thr-Phe(F_5)-Cys(Bzl)-AOMK **MP-cL3**	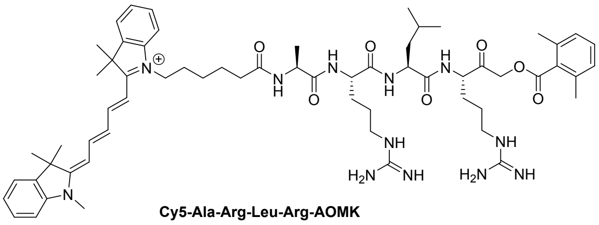 Cy5-Ala-Arg-Leu-Arg-AOMK **MP-pc1**

Table S8 Structures of broad spectrum and selective cathepsin activity based probes labeled with various tags.

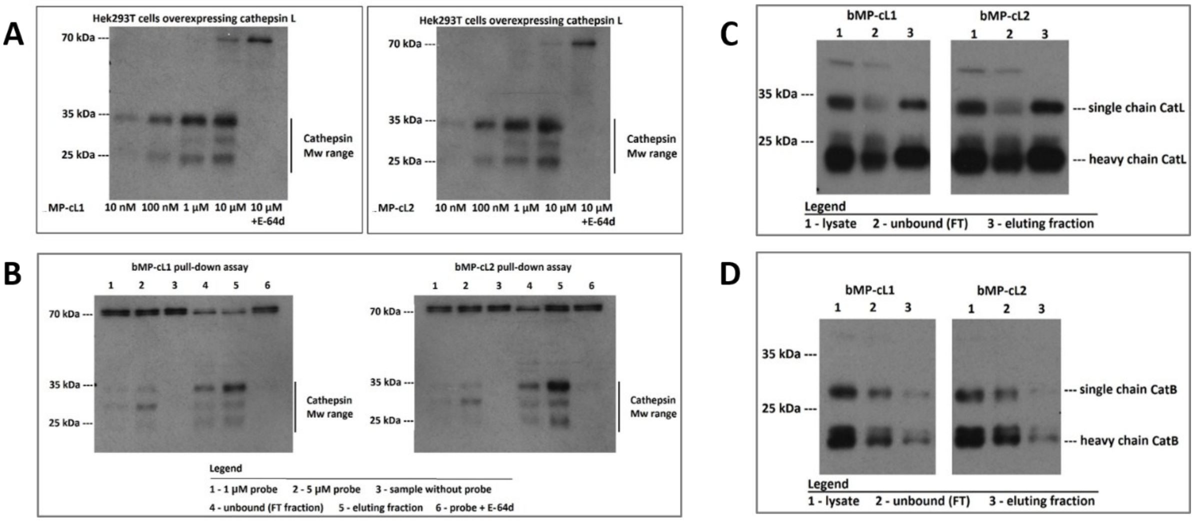

Figure S2 Cathepsin L detection and pull-down experiments in cathepsin L-overexpressing HEK293T cells. **Panel A.** To assess probe uptake, HEK293T cells overexpressing cathepsin L were incubated with various concentrations of ABPs (10 nM - 10 μM). Analysis shows that probes can enter cells and label cathepsin L efficiently at concentrations as low as 100 nM. **Panel B.** To test probe applicability, we incubated HEK293T cells overexpressing cathepsin L with probes and assessed affinity enrichment of biotinylated proteins. While MP-cL1 and MP-cL2 probes showed significant enrichment (compare labeled lysate with the eluting fraction after pulldown), E-64d treatment blocked the signal in the cathepsin MW range. **Panel C and D.** To validate cathepsin L/B selectivity we performed immunologic detection of cathepsin L and B after a pulldown assay in HEK293T cells overexpressing cathepsin L. Strong signals for cathepsin L single and heavy chain (Panel C) and B single and heavy chain (panel D) were detected in lysates. In the unbound fraction, strong signals in the eluting fraction were observed only for cathepsin L.

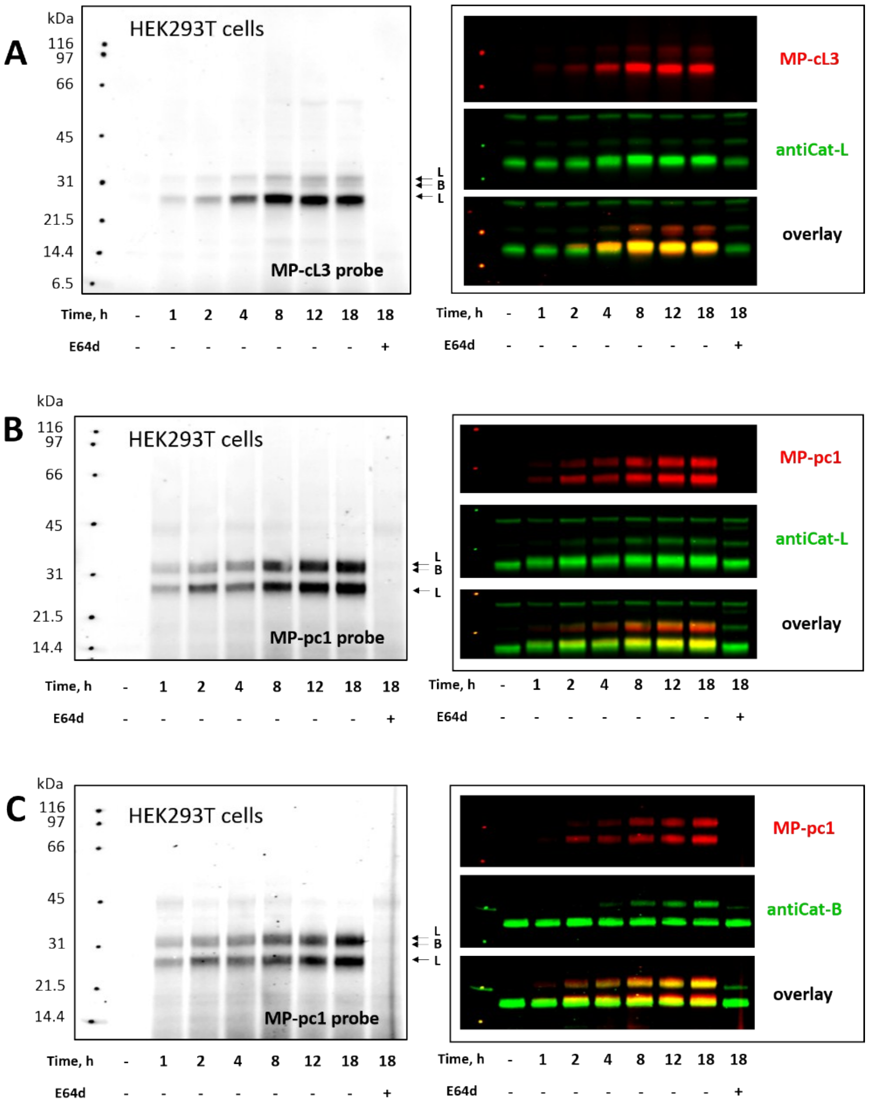

Figure S3 Cathepsins labeling by two Cy5-tagged probes in HEK293T cells. **Panel A.** Cells (100,000/well/mL) were incubated with MP-cL3 (1µM) probe for 0-18 hours. Next, cells were harvested and subjected for Western blotting. MP-cL3 probe is very selective toward cathepsin L even after 18 hours of incubation (only residual cathepsin B activity is detected). However, these cells demonstrate the different cathepsin L processing than MDA-MB-231, thus mostly heavy chain of cathepsin L is labeled. **Panel B and Panel C.** Cells (100,000/well/mL) were incubated with pan-specific MP-pc1 (1µM) probe for 0-18 hours. Next, cells were harvested and subjected for Western blotting. The probe-antibody overlay indicates that MP-pc1 probe binds to cathepsin L (**Panel B**) and cathepsin B (**Panel C**).

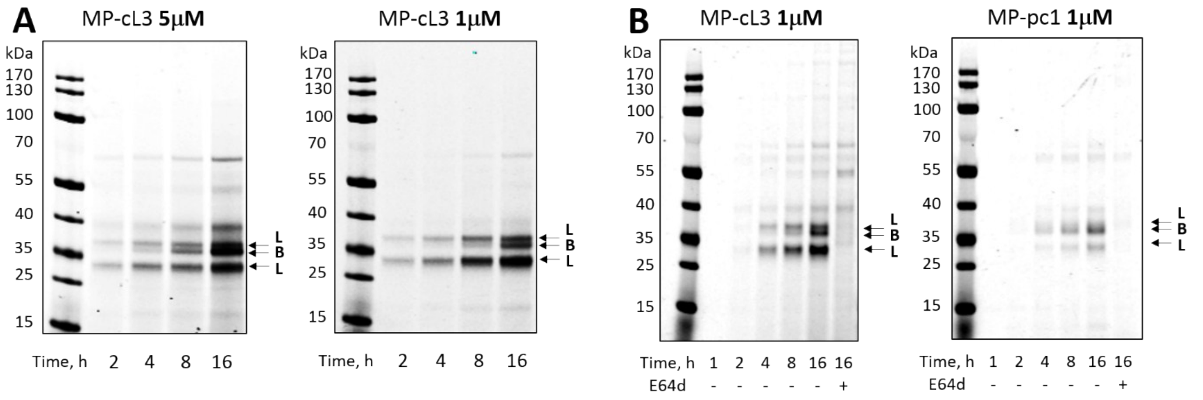

Figure S4 Labeling of cathepsin L and B in living MDA-MB-231 cells using Cy5-labeled activity based probes. **Panel A.** Cells were incubated with cathepsin L selective probe (1μM and 5μM) for 2-16 hours. 5μM of MP-cL3 probe specifically labels cathepsin L only up to two hours, between 4 and 8 hours probe labels also cathepsin B, and after 16 hours this probe labels mostly cathepsin B. However, at the 1μM this probe is much more selective and labels exclusively cathepsin L up to 8 hours. After 16 hours probe labels also cathepsin B. **Panel B.** MP-cL3 probe labels only cathepsin L (up to 8 hours), while MP-c1 probe labels mostly cathepsin B (between 4-16 hours). Cells preincubated with E64d (25μM, 2 hours) show no cathepsins activity.

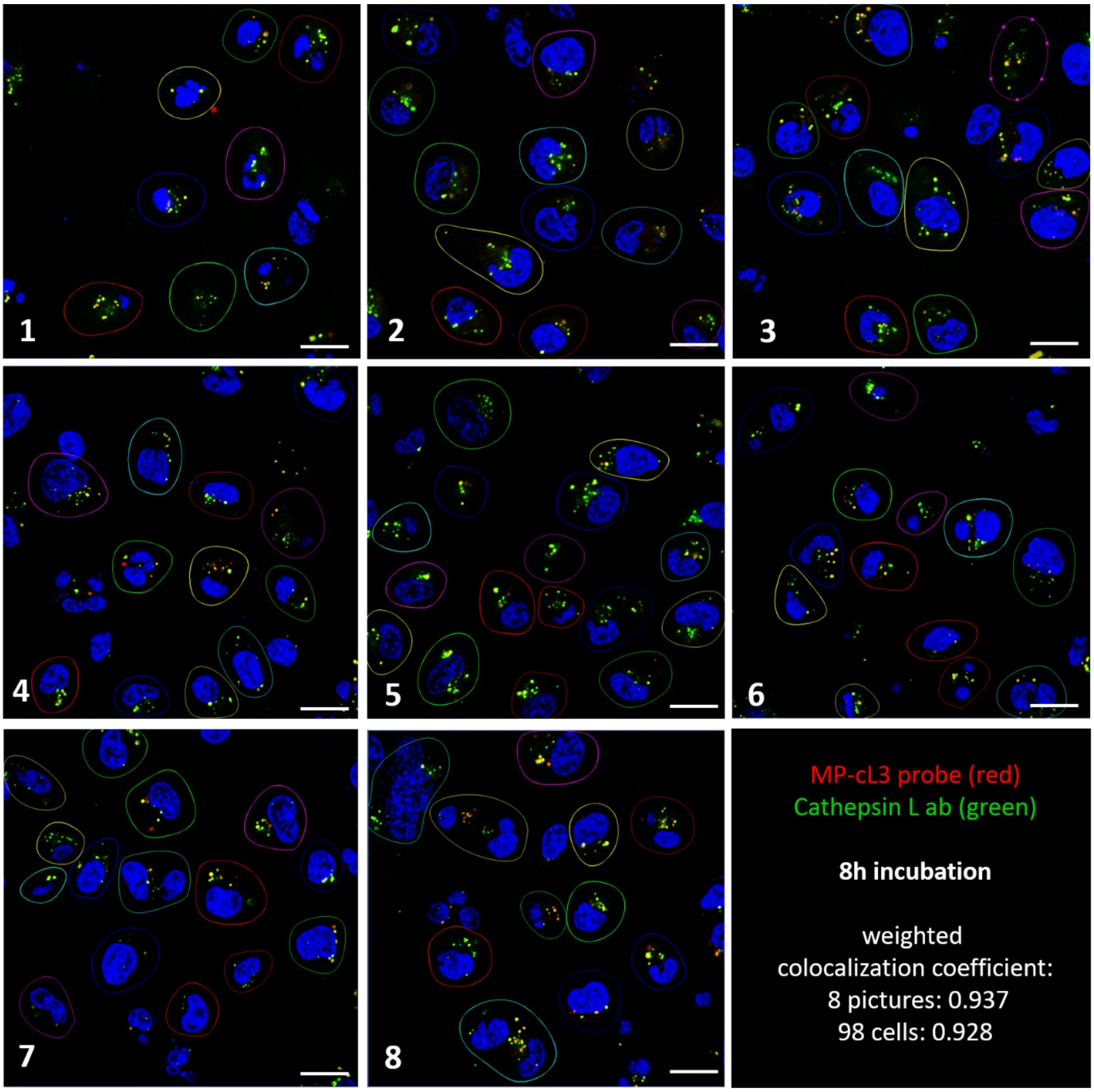

Figure S5 Cathepsin L labeling by MP-cL3 probe in MDA-MB-231 cells. The figure represents eight slides from two independent experiments. MDA-MB-231 cells were incubated with MP-cL3 (red) probe (1µM) for 8 hours, and then were fixed with methanol, stained with cathepsin L antibody (green) and subjected for confocal fluorescence microscopy. Results demonstrate that MP-cL3 can selectively label active cathepsin L when incubated with cells up to 8 hours. The aggregated weighted colocalization coefficient from eight slides was calculated. Circles around cells demonstrate the area taken to calculate weighted colocalization coefficient within single cells. Scale bar 20 µm.

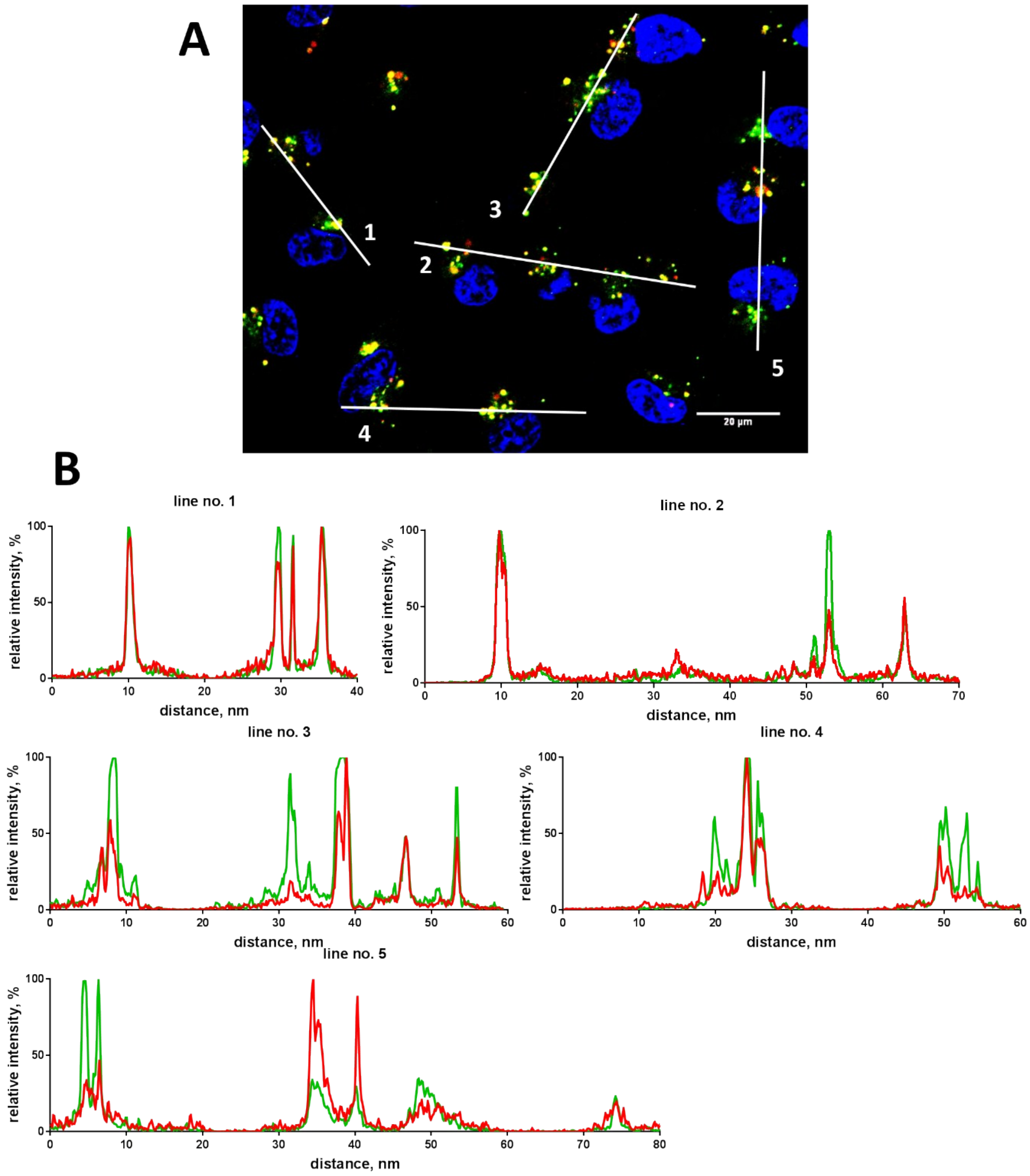

Figure S6 Colocalization between cathepsin L ABP and cathepsin L antibody. **Panel A** MDA-MB-231 cells were incubated with MP-cL3 (red) probe (1μM) for 8 hours, and then were fixed with methanol, stained with cathepsin L antibody (green) and subjected for confocal fluorescence microscopy (DAPI is colored blue). **Panel B** Graphs present pixel intensity from the five lines drawn on the image. The intensity in both channels (red for ABP, and green for cathepsin antibody) was adjusted to 100% (y axis). On the x axis the length of line (in nanometers) is presented. The high weighted colocalization between red and green channels is indicated by overlapping traces (high intensity red signals correlate with high intensity green signals, and the same is true for low intensity signals).

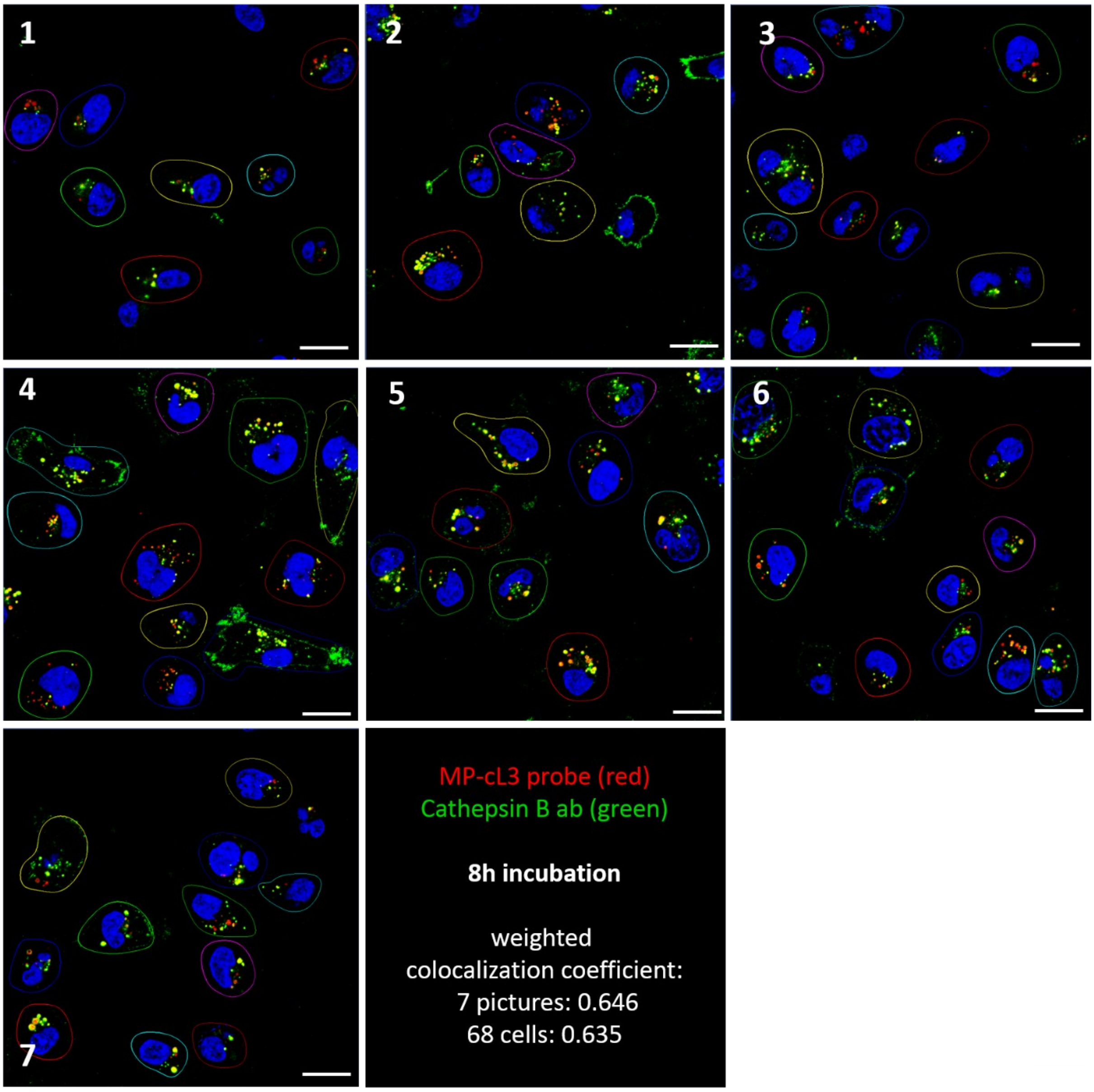

Figure S7 Cathepsin L labeling by MP-cL3 probe in MDA-MB-231 cells. The figure represents seven slides from two independent experiments. To ensure cathepsin L specific labeling, cells were incubated with MP-cL3 (red) probe (1 μM) for 8 hours, and then were fixed with methanol, stained with cathepsin B antibody (green) and subjected for confocal fluorescence microscopy. Results demonstrate that the red spots (active cathepsin L) partially overlays with cathepsin B, indicating that these two enzymes are located in the same lysosomes. However, there are also some vesicles where only cathepsin B is present. The aggregated weighted colocalization coefficient from eight slides was calculated. Circles around cells represent the area taken to calculate weighted colocalization coefficient within single cells. Scale bar 20 μm.

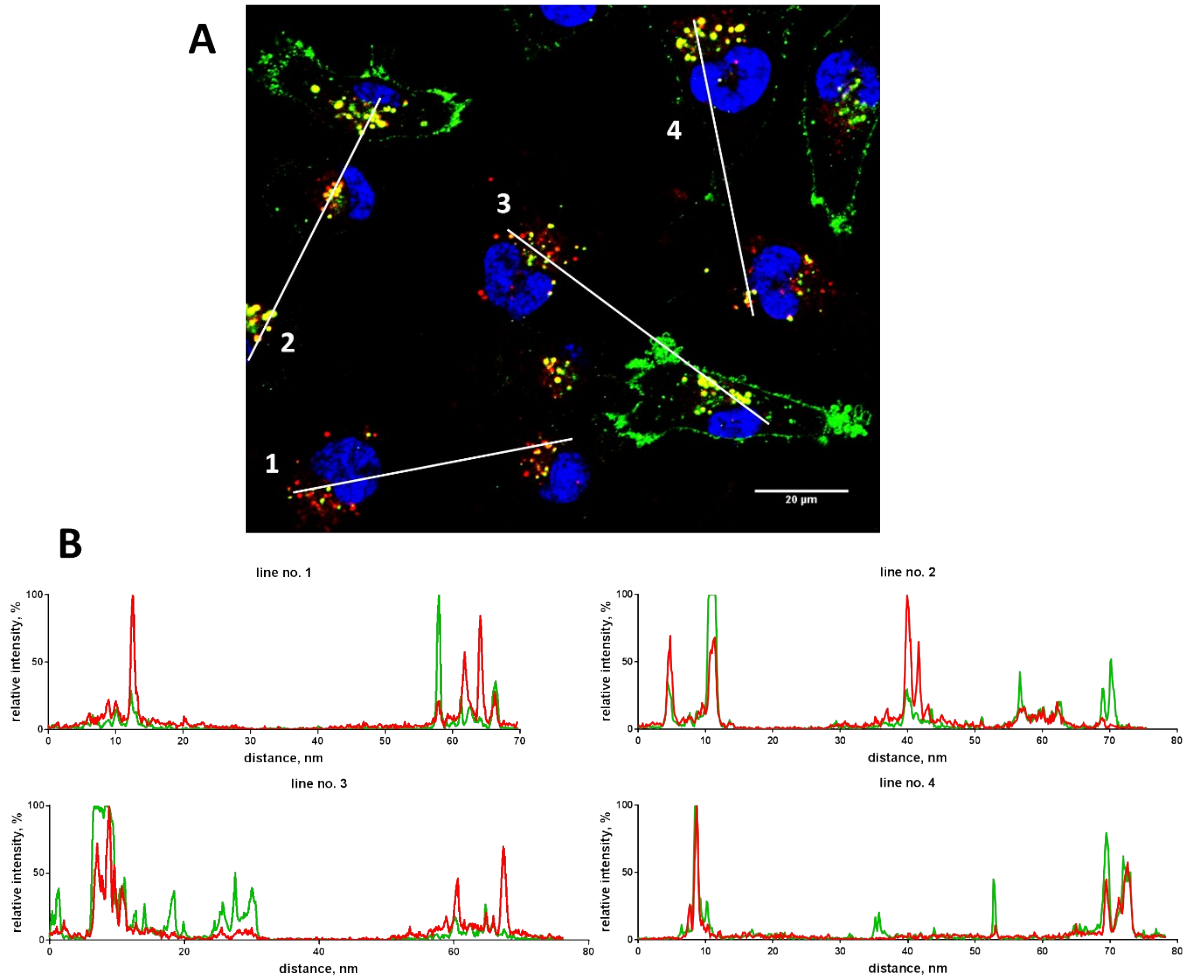

Figure S8 Colocalization between cathepsin L ABP and cathepsin B antibody. **Panel A** MDA-MB-231 cells were incubated with MP-cL3 (red) probe (1µM) for 8 hours, and then were fixed with methanol, stained with cathepsin B antibody (green) and subjected for confocal fluorescence microscopy (DAPI is colored blue). **Panel B** Graphs present pixel intensity from the five lines drawn on the image. The intensity in both channels (red for ABP, and green for cathepsin antibody) was adjusted to 100% (y axis). On the x axis the length of line (in nanometers) is presented. The poor weighted colocalization between red and green channels is indicated by nonoverlapping traces (high intensity red signals do not correlate with high intensity green signals, and the same is true for low intensity signals).

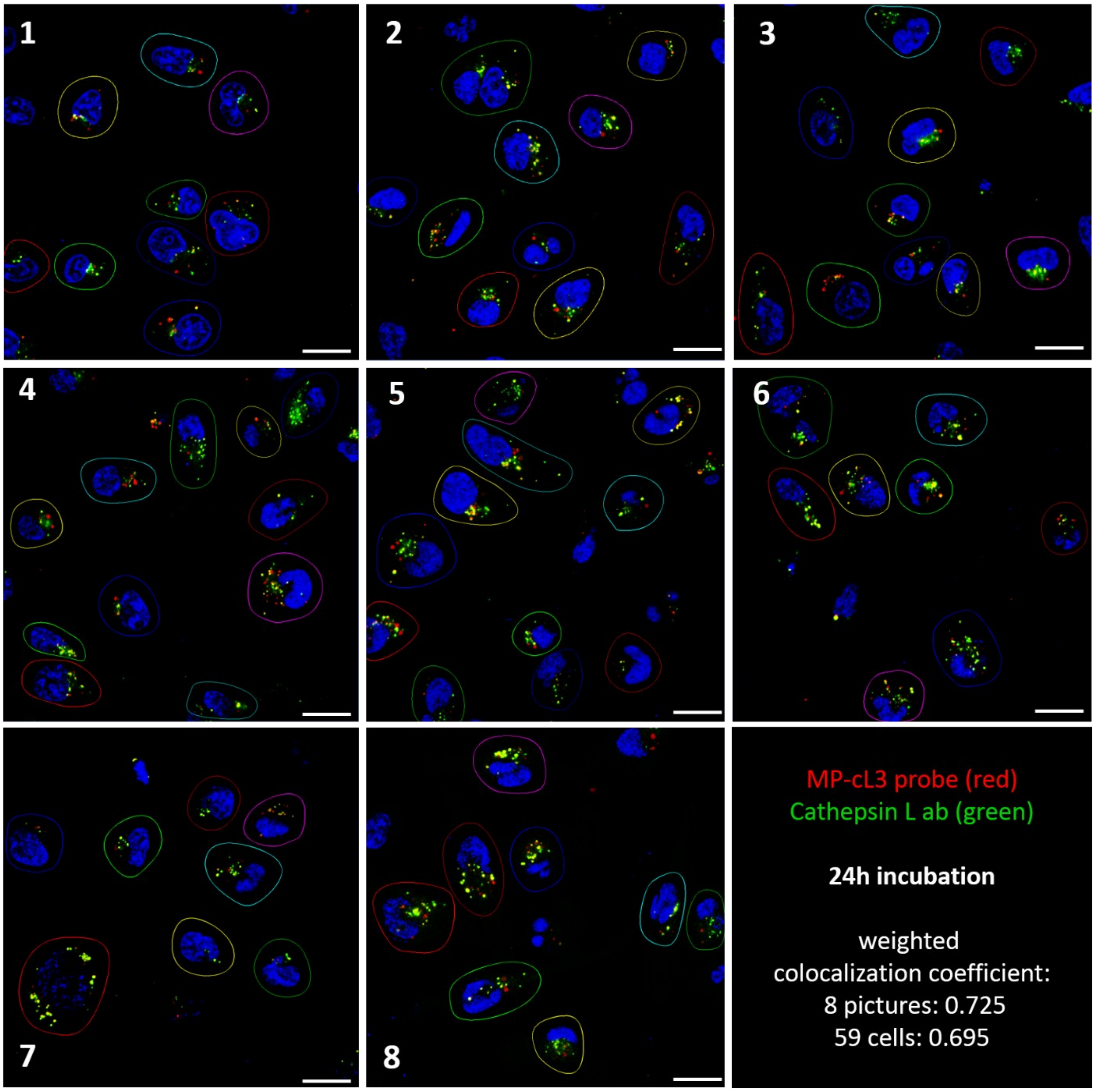

Figure S9 Cathepsin L labeling by MP-cL3 probe in MDA-MB-231 cells. The figure represents eight slides from two independent experiments. Cells were incubated with MP-cL3 (red) probe (1 μM) for 24 hours, and then were fixed with methanol, stained with cathepsin L antibody (green) and subjected for confocal fluorescence microscopy. Results demonstrate that after prolonged incubation MP-cL3 probes loses the selectivity and labels also cathepsin B (as indicated by immunoblotting). The aggregated weighted colocalization coefficient from eight slides was calculated. Circles around cells demonstrate the area taken to calculate weighted colocalization coefficient within single cells. Scale bar 20 μm.

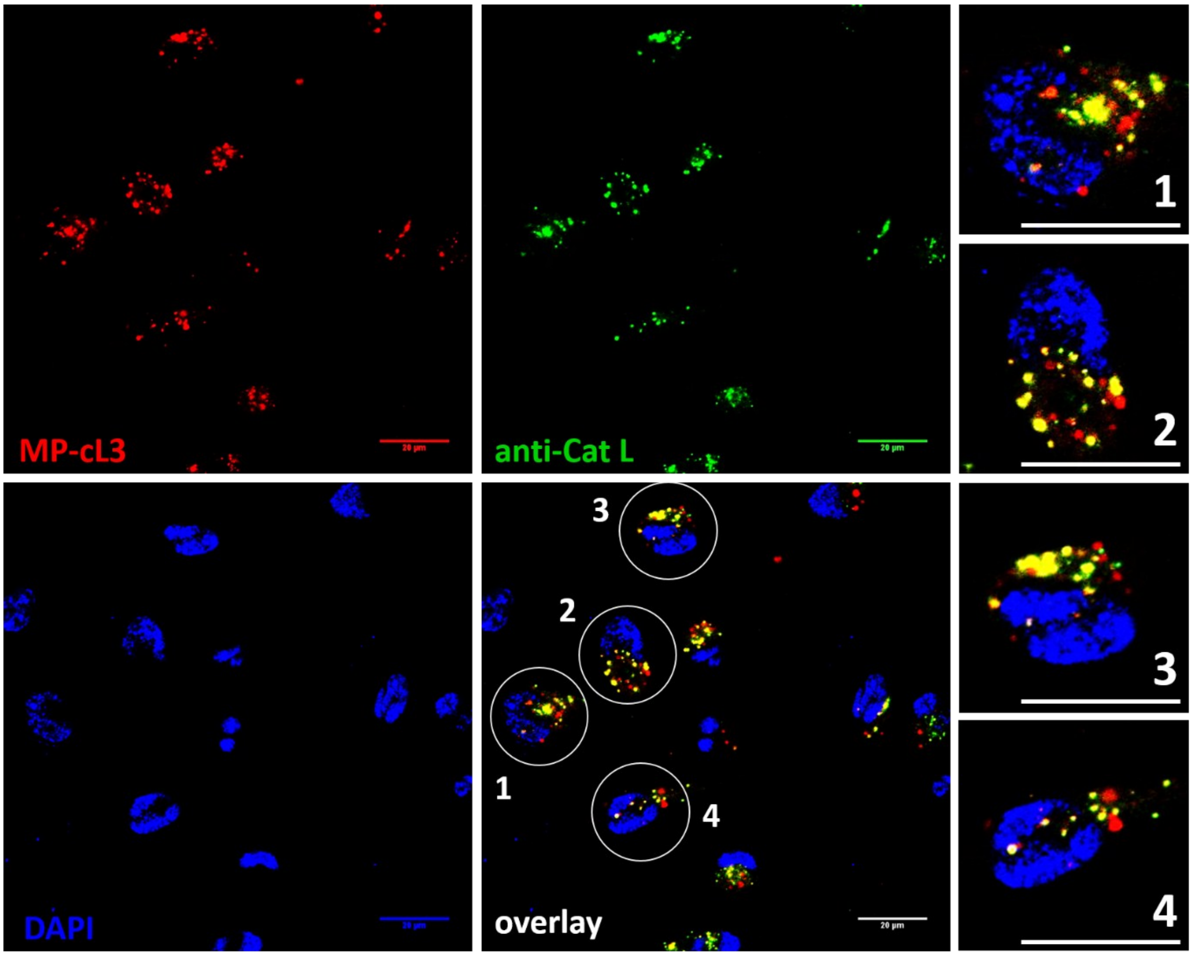

Figure S10 Cathepsin L labeling by MP-cL3 probe in MDA-MB-231 cells. In the experiment, cells were incubated with MP-cL3 (red) probe (1 µM) for 24 hours, and then were fixed with methanol, stained with cathepsin L antibody (green) and subjected for confocal fluorescence microscopy. Results demonstrate that after prolonged incubation MP-cL3 probes loses the selectivity and labels also cathepsin B (as indicated by immunoblotting). Scale bar 20 µm.

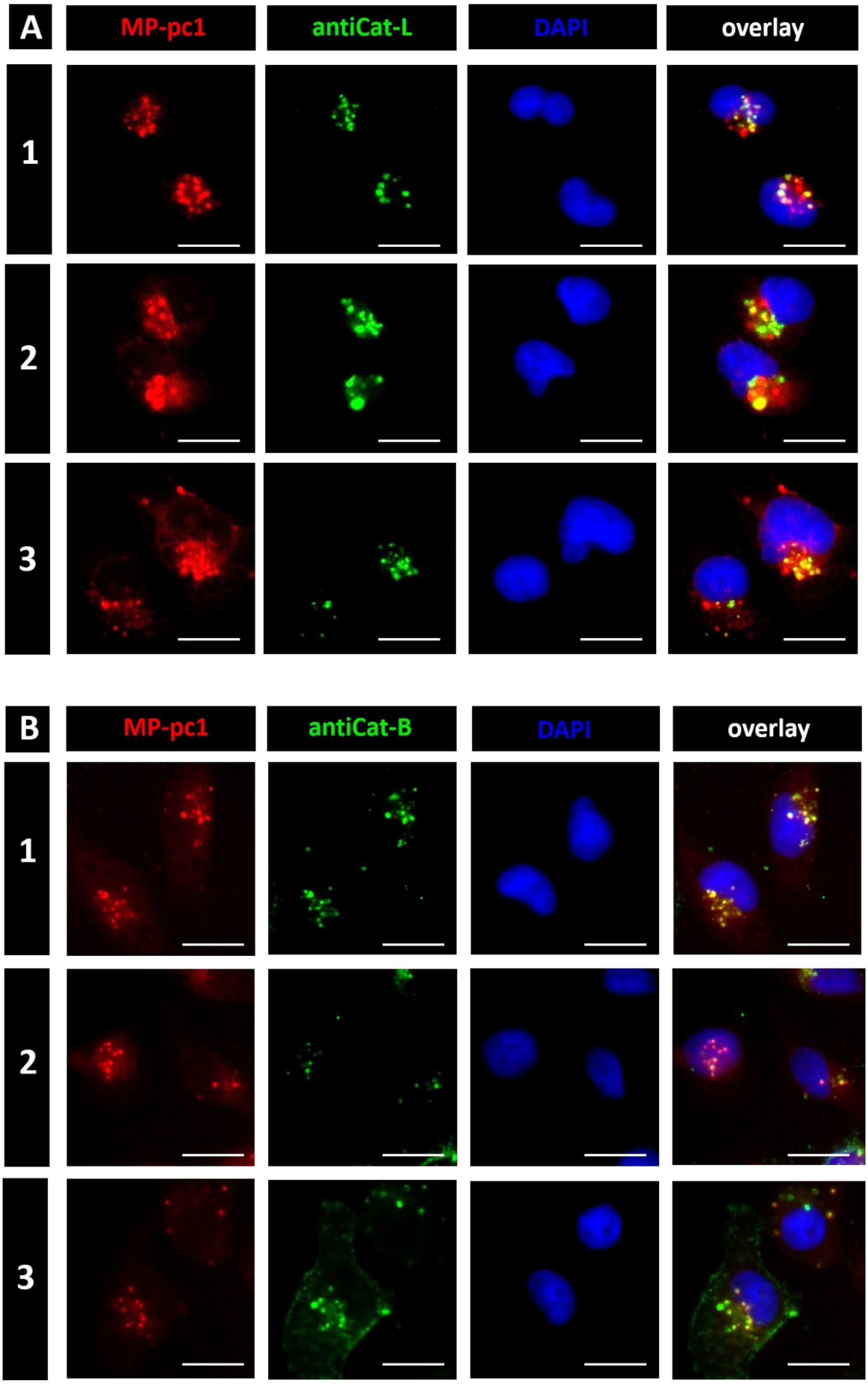

Figure S11 Cathepsins labeling by MP-pc1 probe in MDA-MB-231 cells. General cathepsins probe, MP-pc1 (1 μM) was incubated with cells for 8 hours. Then cells were fixed with methanol and incubated with cathepsin L either cathepsin B antibodies. The fluorescence microscopy analysis reveals that this probe labels both enzymes, however, cathepsin B shows better co-localization with the probe. Scale bar 15 μm.

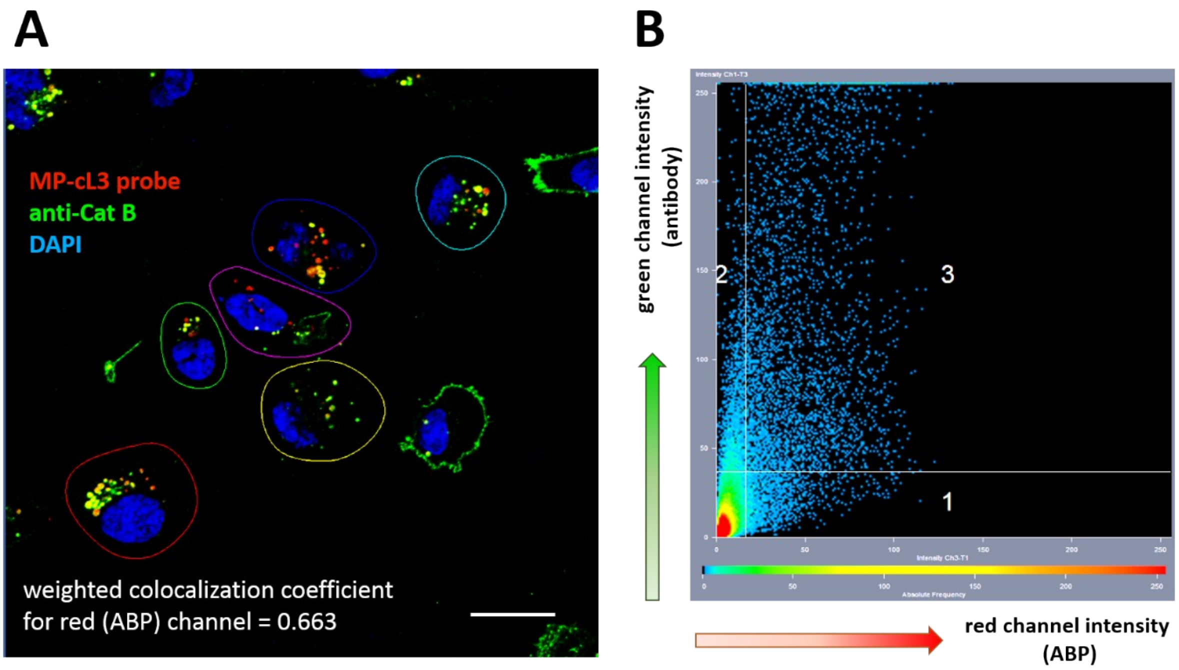

Figure S12 Calculation of weighted colocalization coefficient between cathepsin L probe and cathepsin B antibody. **Panel A** Fluorescence microscopy image of MDA-MB-231 cells labeled with MP-cL3 probe and cathepsin B antibody. **Panel B** All the pixels from the picture were used to calculate weighted colocalization coefficient between red and green channels. These calculations were performed by summing the pixels in the colocalized regions (red and green; Quadrant 3) and dividing by the sum of pixels in red channel (ABP; Quadrant 1 + Quadrant 3). The value of each pixel was equal to its intensity value (from 0 to 1). To eliminate the red and green channels staining background, we set crosshairs according to single label controls.

No.	Structure	$[M+H]^+$ calculated	$[M+H]^+$ measured
**ACC SUBSTRATES**			
1	Ac-His-Arg-Phe-Arg-ACC	857.4171	857.4255
2	Ac-Dap-Orn-Phe(3-Cl)-Arg-ACC	798.3454	798.3456
3	Ac-Dap-Arg-Phe(3-Cl)-Arg-ACC	840.3672	840.3655
4	Ac-Dab-Orn-Phe(3-Cl)-Arg-ACC	812.3611	812.3622
5	Ac-Dab-Arg-Phe(3-Cl)-Arg-ACC	854.3829	854.3955
6	Ac-Dap-Orn-Phe(3-Cl)-Lys-ACC	770.3393	770.3366
7	Ac-Dap-Orn-Phe(3-Cl)-hArg-ACC	812.3611	812.3612
8	Ac-Dap-Orn-Phe(3-Cl)-Leu-ACC	755.3284	755.3258
9	Ac-Dap-Orn-Phe(3-Cl)-Glu(Bzl)-ACC	861.3338	861.3545
10	Ac-Dap-Orn-Phe(3-Cl)-Cys(Bzl)-ACC	835.3004	835.3055
11	Ac-Dap-Orn-Phe(3-Cl)-Cys(MeBzl)-ACC	849.3161	849.3126
12	Ac-Dap-Orn-Phe(3-Cl)-Cys(MeOBzl)-ACC	865.3110	865.3122
13	Ac-Dap-Orn-Phe(3-Cl)-Nle(OBzl)-ACC	861.3702	861.3750
14	Ac-Dap-Orn-Phe(3-Cl)-Lys(2-Cl-Z)-ACC	938.3371	938.3355
15	Ac-Tic-Orn-Phe(4-F)-Leu-ACC	812.3783	812.3788
16	Ac-His-*D*Arg-Phe(3,4-F_2)-Leu-ACC	850.3812	850.3814
17	Ac-His-*D*Leu-Phe(F_5)-Orn-ACC	862.3311	862.3254

18	Ac-Tic-*D*Orn-Phe(2-Cl)-Lys-ACC	843.3597	843.3655
19	Ac-Met(O)-*D*Arg-Phe(2-F)-Arg-ACC	885.3841	885.3789
20	Ac-Ala-*D*Lys-Lys(Ac)-Arg-ACC	786.4262	786.4356
21	Ac-His-*D*Val-Phe(F5)-Arg-ACC	890.3373	890.3389
22	Ac-Tic-Dab-Phe(3,4-F$_2$)-Arg-ACC	859.3703	589.3709
23	Ac-His-*D*Leu-Phe(3,4-Cl2)-Arg-ACC	882.3221	882.3199
24	Ac-Ala-Orn-Phe(3,4-Cl$_2$)-Arg-ACC	817.2955	817.2912
25	Ac-Pip-*D*Leu-Phe(3,4-F$_2$)-Arg-ACC	824.3907	824.3998
26	Ac-His-*D*Val-Phe(F$_5$)-Arg-ACC	890.3373	890.3401
27	Ac-Arg-*D*Val-Phe(F$_5$)-Arg-ACC	909.3795	909.3806
28	Ac-hArg-*D*Val-Phe(F$_5$)-Arg-ACC	923.3951	923.3952
29	Ac-His-*D*Leu-Phe(F$_5$)-Arg-ACC	904.3529	904.3498
30	Ac-His-*D*Gln-Phe(F$_5$)-Arg-ACC	919.3274	919.3244
31	Ac-His-*D*Thr-Phe(F$_5$)-Arg-ACC	892.3165	892.3199
32	Ac-His-*D*Lys-Phe(F$_5$)-Arg-ACC	919.3638	919.3654
33	Ac-His-*D*Val-Phe(2-Cl)-Arg-ACC	834.3454	934.3507
34	Ac-His-*D*Val-Phe(3,4-F$_2$)-Arg-ACC	836.3655	836.3784
35	Ac-His-*D*Val-Phe(3-F)-Arg-ACC	818.3750	818.3789
36	Ac-His-*D*Val-Thr(Bzl)-Arg-ACC	844.4106	844.4256
37	Ac-His-*D*Val-Phe(4-F)-Arg-ACC	818.3750	818.3755
38	Ac-His-*D*Thr-Phe(F$_5$)-Lys-ACC	952.3311	952.3348
39	Ac-His-*D*Thr-Phe(F$_5$)-Leu-ACC	847.3202	847.3256
40	Ac-His-*D*Thr-Phe(F$_5$)-hArg-ACC	904.3529	904.3597
41	Ac-His-*D*Thr-Phe(F$_5$)-Glu(Bzl)-ACC	953.3257	953.3356
42	Ac-His-*D*Thr-Phe(F$_5$)-Cys(Bzl)-ACC	927.2923	927.3089
43	Ac-His-*D*Thr-Phe(F$_5$)-CysMeBzl)-ACC	941.3079	941.3077
44	Ac-His-*D*Thr-Phe(F$_5$)-Cys(MeOBzl)-ACC	957.3029	957.3066
45	Ac-His-*D*Thr-Phe(F$_5$)-Nle(OBzl)-ACC	953.3621	953.3698
46	Ac-His-*D*Thr-Phe(F$_5$)-Lys(2-Cl-Z)-ACC	1030.3289	1030.3211
**ACTIVITY BASED PROBES**			
		$[M+H]^+$ calculated	$[M+H]^+$ measured
47	biotin-6-ahx-His-*D*Thr-Phe(F$_5$)-Arg-AOMK **(MP-cL1)**	1135.4817	1135.4812
48	biotin-6-ahx-His-*D*Thr-Phe(F$_5$)-Lys-AOMK **(MP-cL2)**	1107.4755	1107.4751
49	Cy5-6-ahx-His-*D*Thr-Phe(F$_5$)-Cys(Bzl)-AOMK **(MP-cL3)**	1410.6419	1410.6027
50	BODIPY$_{FL}$-6-ahx-Ala-Arg-Leu-Arg-AOMK **(MP-pc2)**	1048.6099	1048.6074
51	Cy3-6-ahx-Ala-Arg-Leu-Arg-AOMK **(MP-pc3)**	606.8864	606.8872
52	Cy5-Ala-Arg-Leu-Arg-AOMK **(MP-pc1)**	563.3522	563.3511

Table S9 Molecular masses of peptide fluorogenic substrates and activity based probes used in this study. ACC-labeled substrates displayed at least 95% purity, since the activity based probes displayed over 90% of purity.

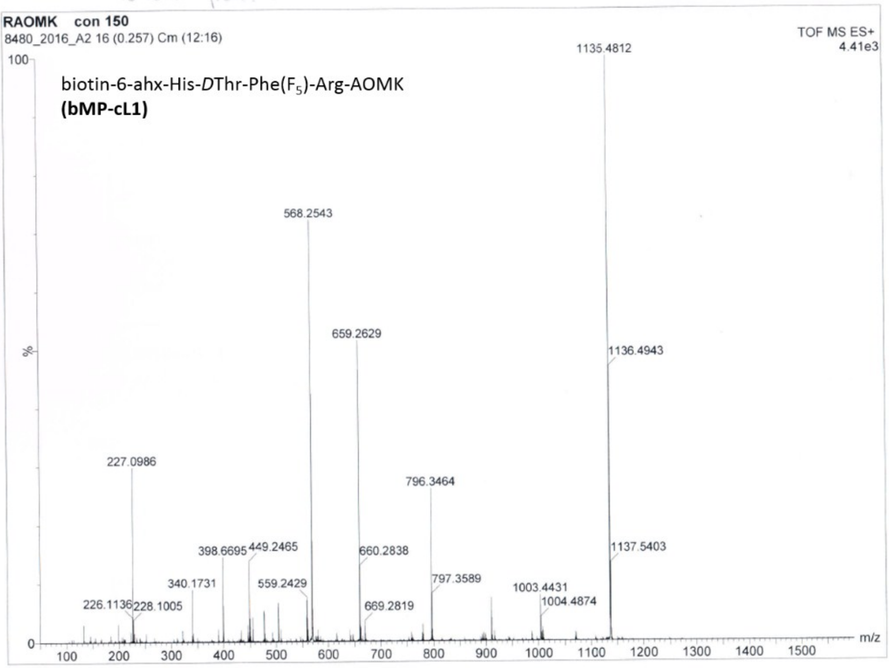

Figure S13 m/z analysis of cathepsin L activity based probe (biotin-6-ahx-His-*D*Thr-Phe(F_5)-Arg-AOMK; bMP-cL1).

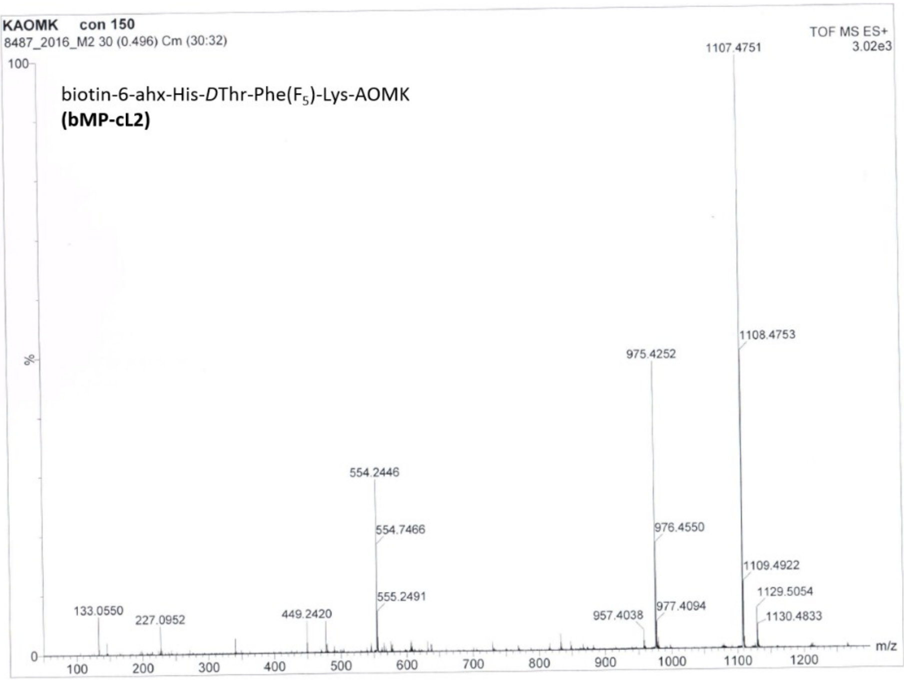

Figure S14 m/z analysis of cathepsin L activity based probe (biotin-6-ahx-His-*D*Thr-Phe(F_5)-Lys-AOMK; bMP-cL2).

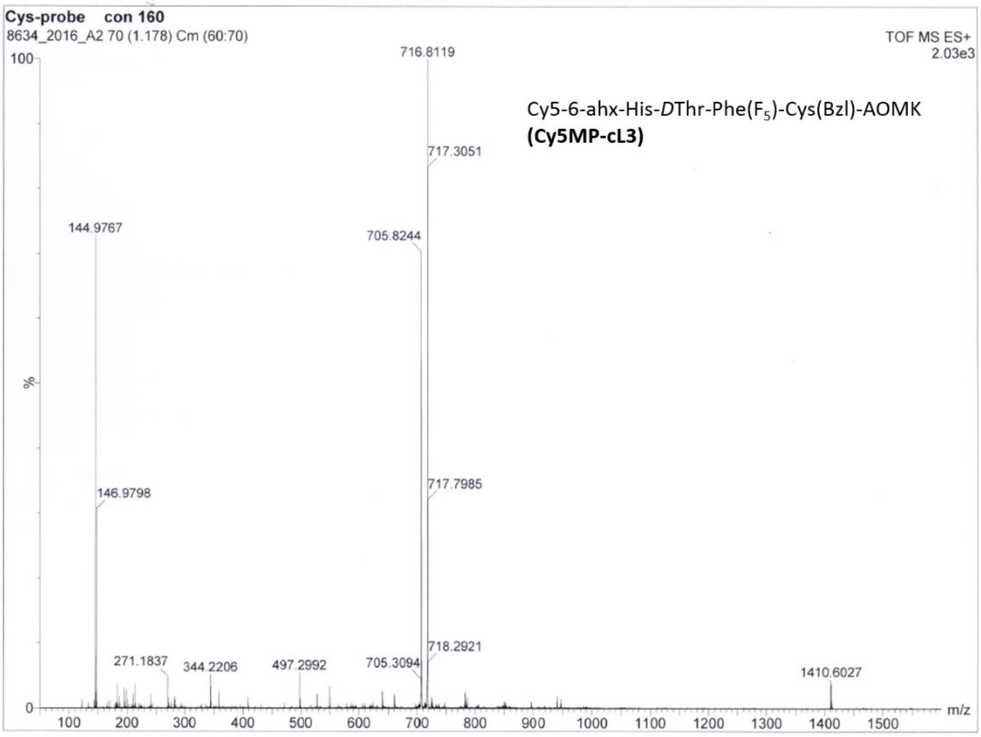

Figure S15 m/z analysis of cathepsin L activity based probe (Cy5-6-ahx-His-*D*Thr-Phe(F_5)-Cys(Bzl)-AOMK; Cy5MP-cL3).

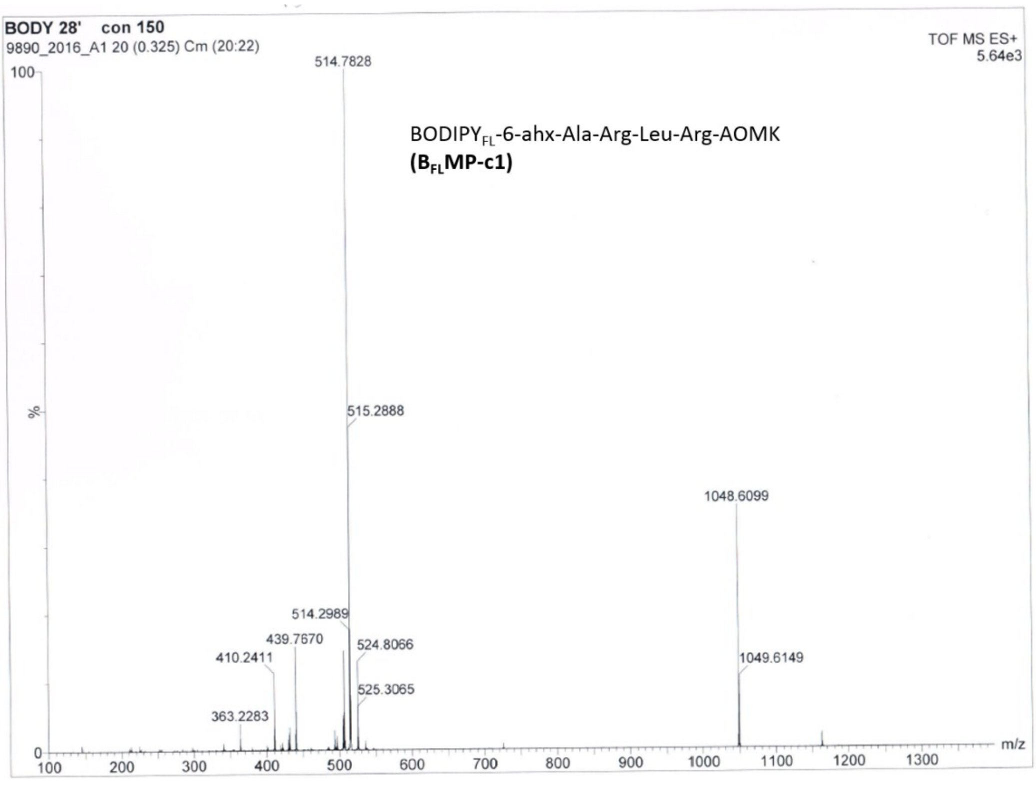

Figure S16 m/z analysis of broad spectrum cathepsins activity based probe (BDP-6-ahx-ARLR-AOMK; B_{FL}MP-c1).

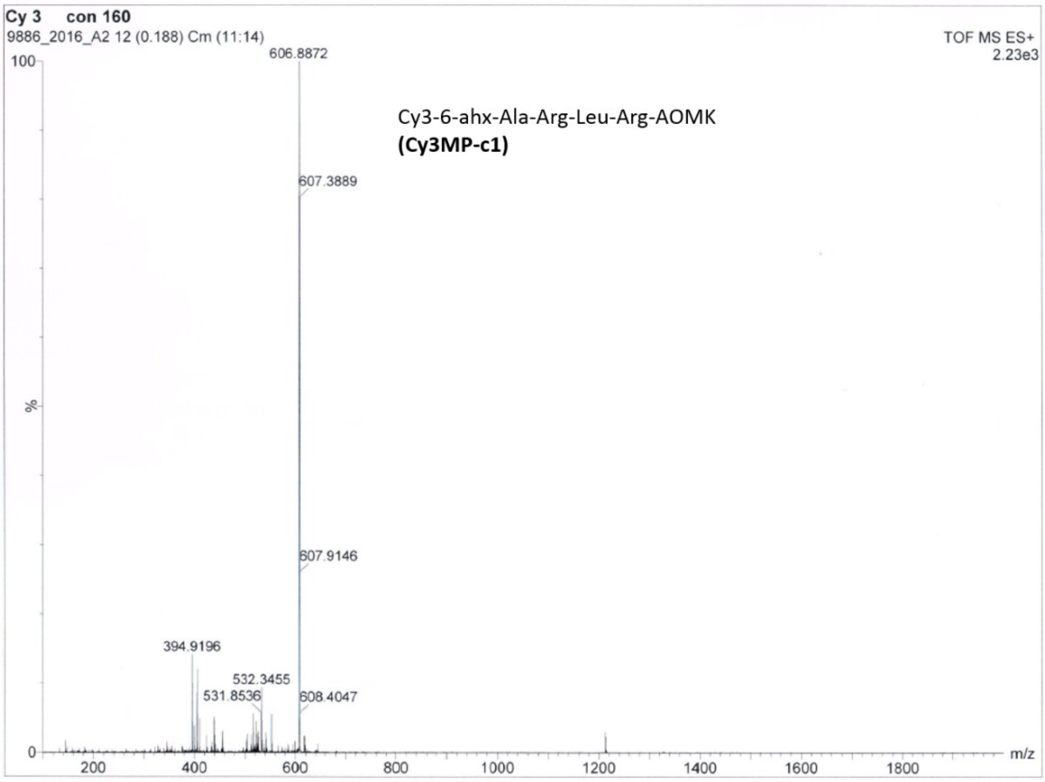

Figure S17 m/z analysis of broad spectrum cathepsins activity based probe (Cy3-6-ahx-ARLR-AOMK; Cy3MP-c1).

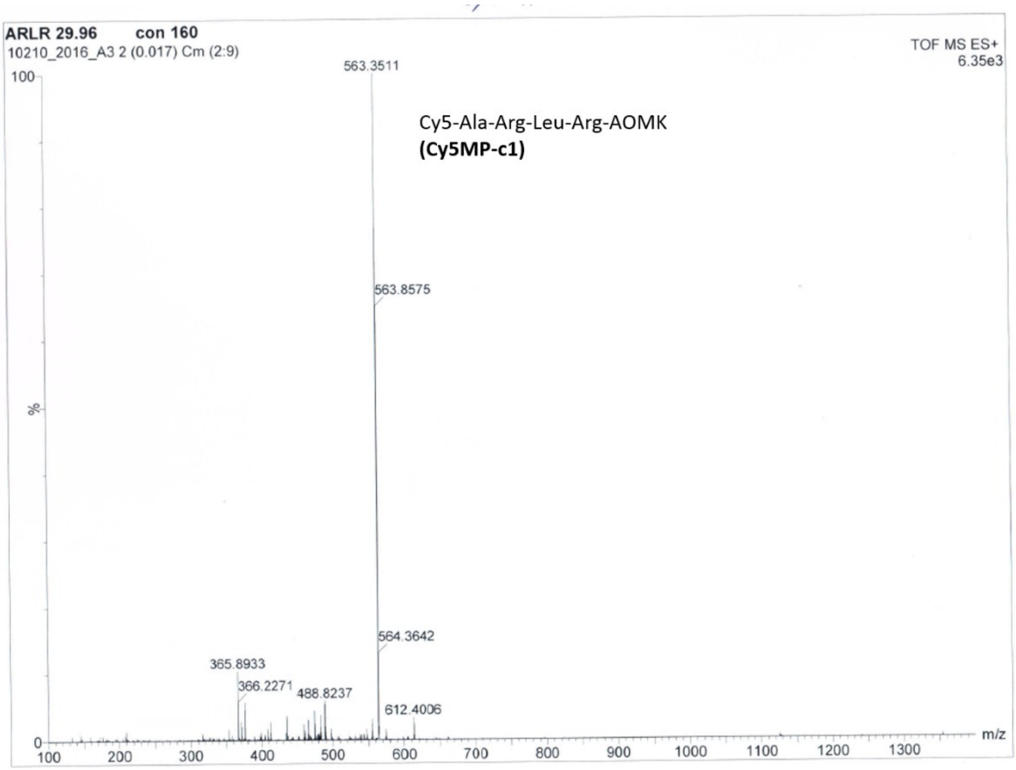

Figure S18 m/z analysis of broad spectrum cathepsins activity based probe (Cy5-ARLR-AOMK; Cy5MP-c1).

No	Structure + code	No	Structure + code	No	Structure + code
1	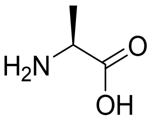 *L*-Ala	2	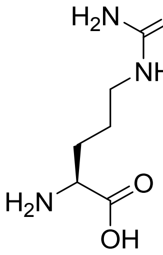 *L*-Arg	3	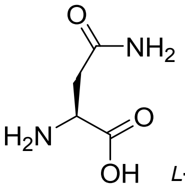 *L*-Asn
4	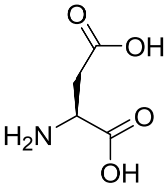 *L*-Asp	5	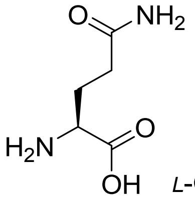 *L*-Gln	6	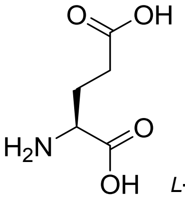 *L*-Glu
7	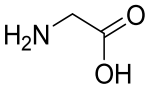 Gly	8	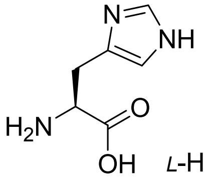 *L*-His	9	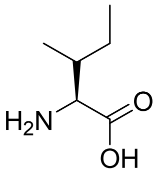 *L*-Ile
10	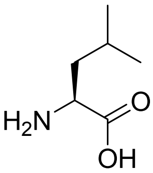 *L*-Leu	11	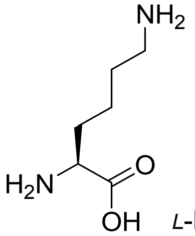 *L*-Lys	12	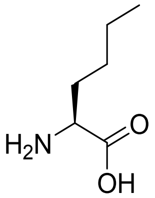 *L*-Nle
13	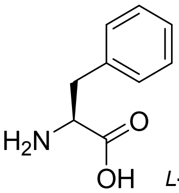 *L*-Phe	14	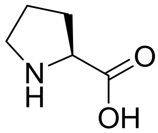 *L*-Pro	15	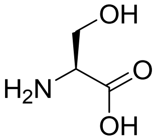 *L*-Ser
16	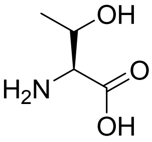 *L*-Thr	17	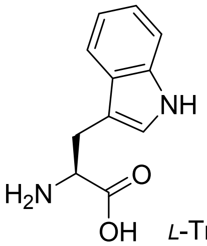 *L*-Trp	18	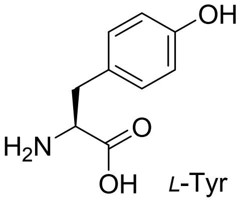 *L*-Tyr
19	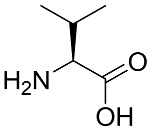 *L*-Val	20	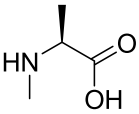 *L*-MeAla	21	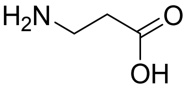 *L*-βAla
22	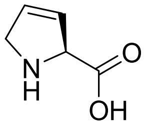 *L*-dhPro	23	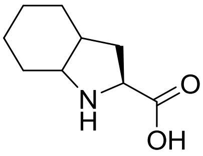 *L*-Oic	24	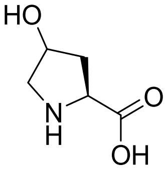 *L*-Hyp

25 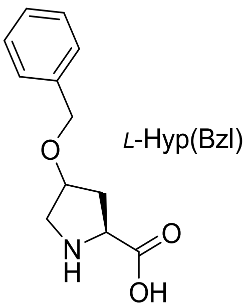 *L*-Hyp(Bzl)

26 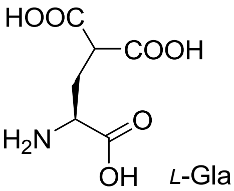 *L*-Gla

27 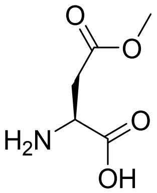 *L*-Asp(Me)

28 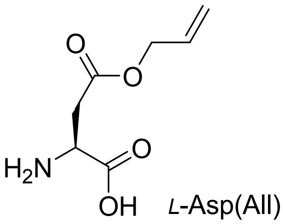 *L*-Asp(All)

29 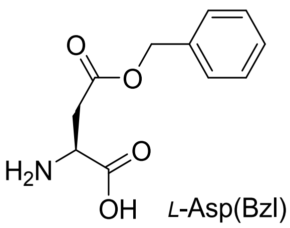 *L*-Asp(Bzl)

30 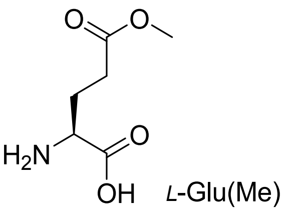 *L*-Glu(Me)

31 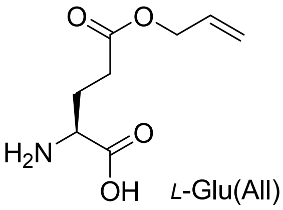 *L*-Glu(All)

32 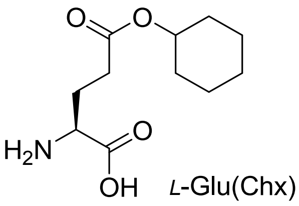 *L*-Glu(Chx)

33 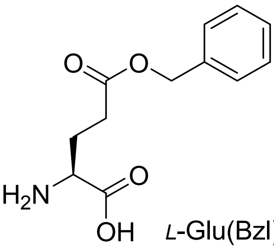 *L*-Glu(Bzl)

34 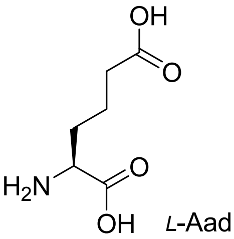 *L*-Aad

35 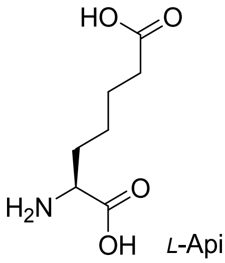 *L*-Api

36 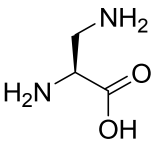 *L*-Dap

37 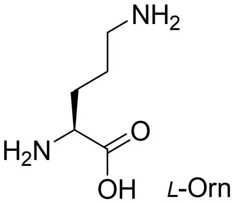 *L*-Orn

38 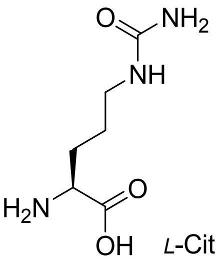 *L*-Cit

39 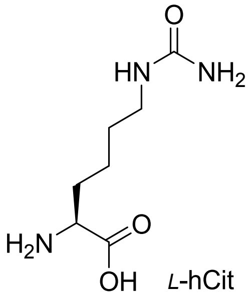 *L*-hCit

40 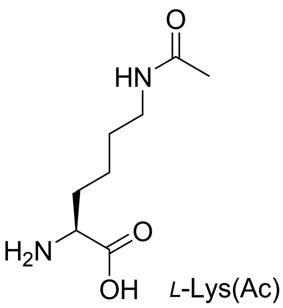 *L*-Lys(Ac)

41 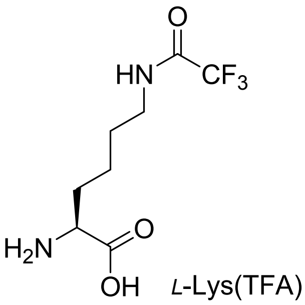 *L*-Lys(TFA)

42 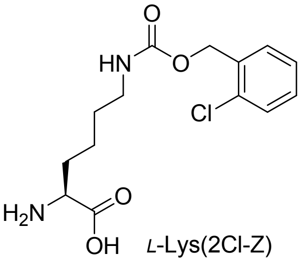 *L*-Lys(2Cl-Z)

43 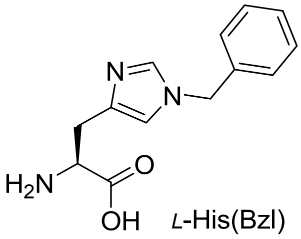 L-His(Bzl)

44 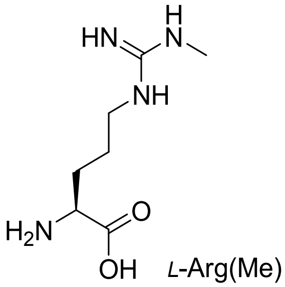 L-Arg(Me)

45 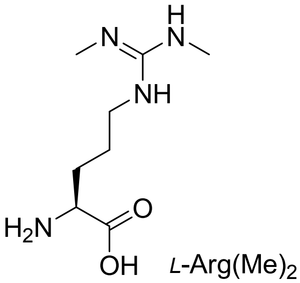 L-Arg(Me)$_2$

46 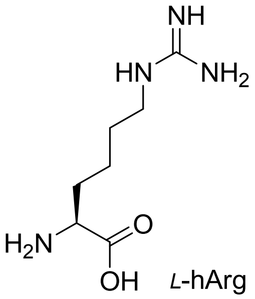 L-hArg

47 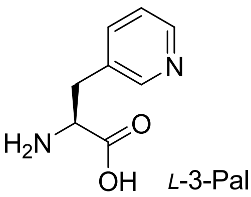 L-3-Pal

48 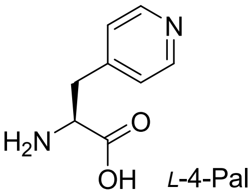 L-4-Pal

49 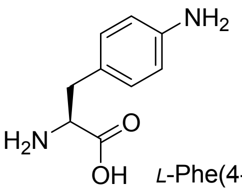 L-Phe(4-NH_2)

50 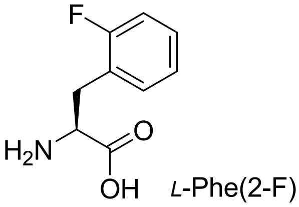 L-Phe(2-F)

51 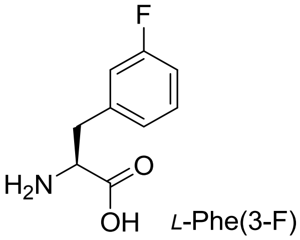 L-Phe(3-F)

52 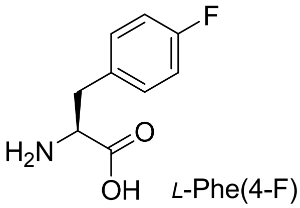 L-Phe(4-F)

53 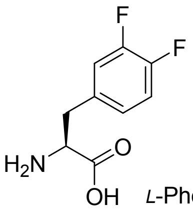 L-Phe(4,3-F_2)

54 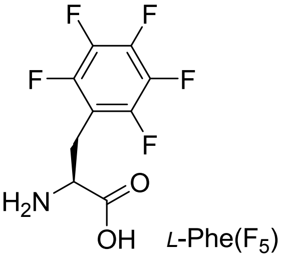 L-Phe(F_5)

55 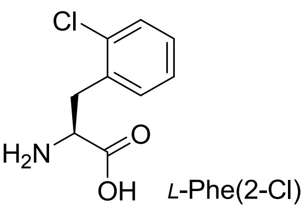 L-Phe(2-Cl)

56 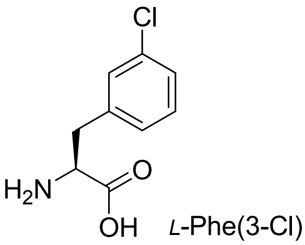 L-Phe(3-Cl)

57 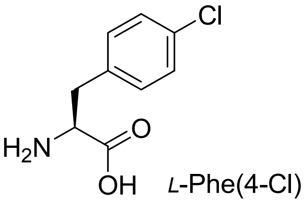 L-Phe(4-Cl)

58 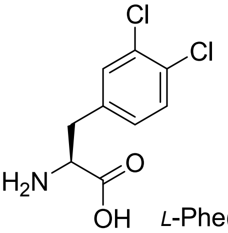 L-Phe(3,4-Cl_2)

59 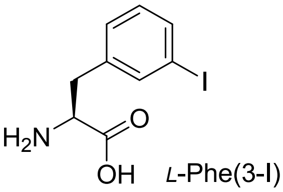 L-Phe(3-I)

60 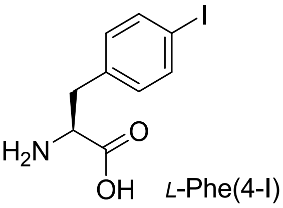 L-Phe(4-I)

61 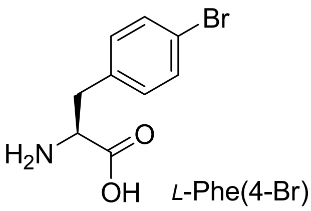 *L*-Phe(4-Br)

62 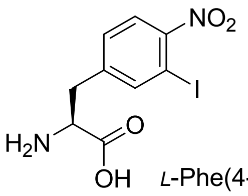 *L*-Phe(4-NO_2)

63 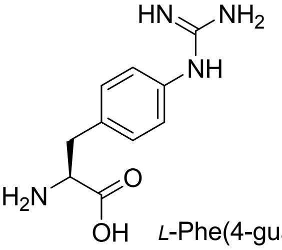 *L*-Phe(4-guan)

64 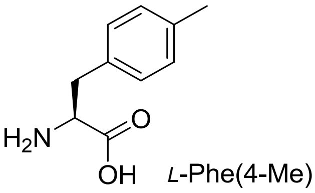 *L*-Phe(4-Me)

65 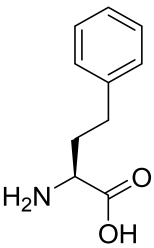 *L*-hPhe

66 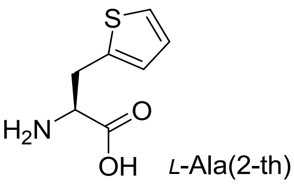 *L*-Ala(2-th)

67 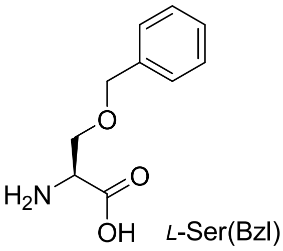 *L*-Ser(Bzl)

68 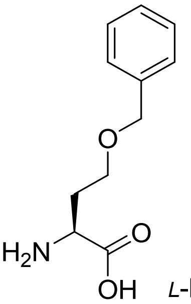 *L*-hSer(Bzl)

69 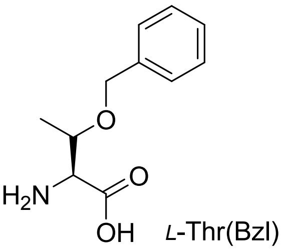 *L*-Thr(Bzl)

70 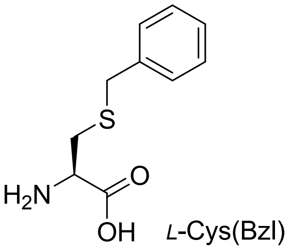 *L*-Cys(Bzl)

71 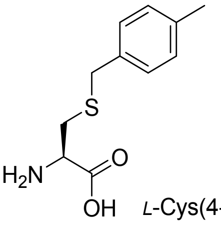 *L*-Cys(4-MeBzl)

72 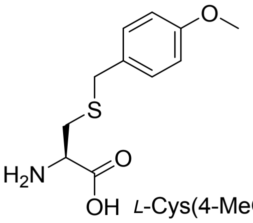 *L*-Cys(4-MeOBzl)

73 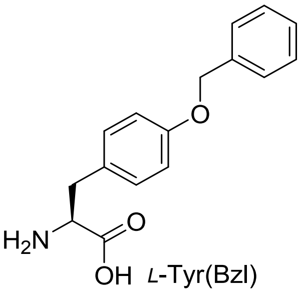 *L*-Tyr(Bzl)

74 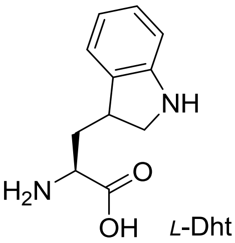 *L*-Dht

75 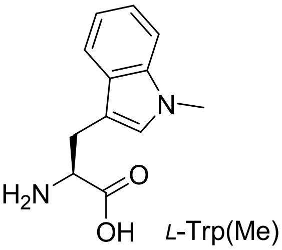 *L*-Trp(Me)

76 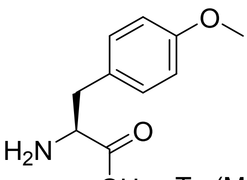 *L*-Tyr(Me)

77 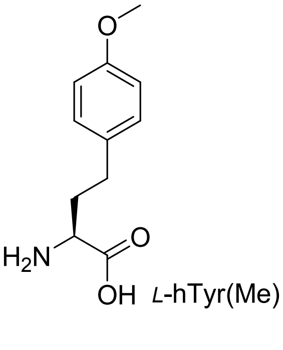 *L*-hTyr(Me)

78 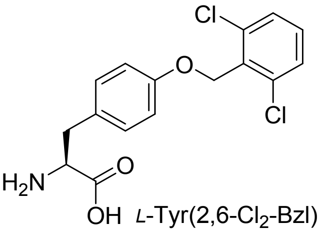 *L*-Tyr(2,6-Cl_2-Bzl)

79 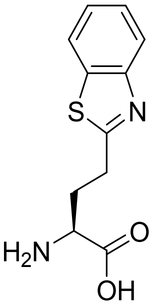 *L*-Abu(Bth)

80 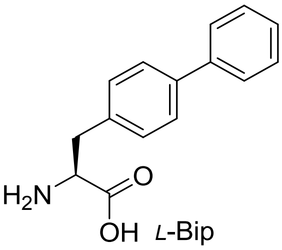 *L*-Bip

81 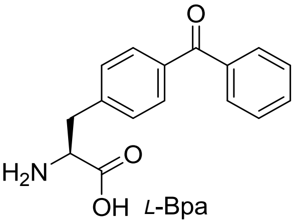 *L*-Bpa

82 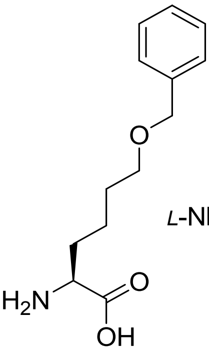 *L*-Nle(*O*-Bzl)

83 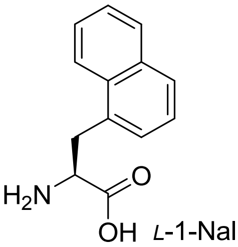 *L*-1-Nal

84 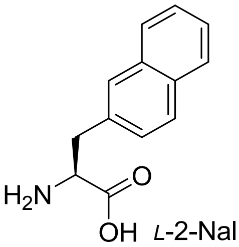 *L*-2-Nal

85 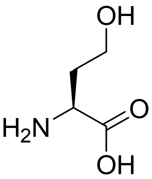 *L*-hSer

86 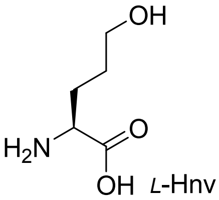 *L*-Hnv

87 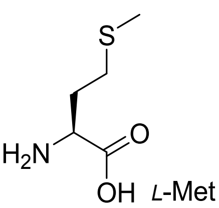 *L*-Met

88 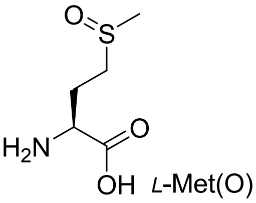 *L*-Met(O)

89 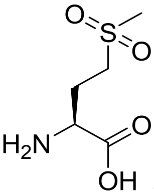 *L*-Met(O_2)

90 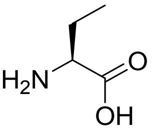 *L*-Abu

91 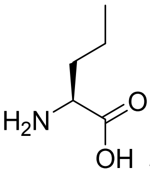 *L*-Nva

92 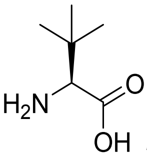 *L*-Tle

93 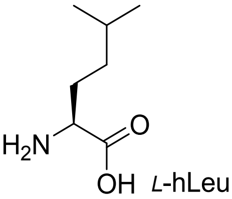 *L*-hLeu

94 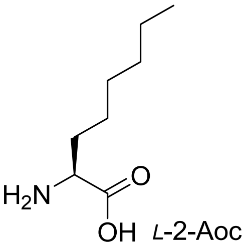 *L*-2-Aoc

95 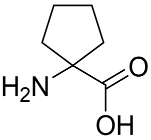 AC5C

96 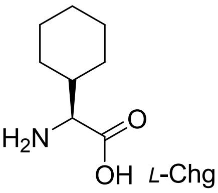 *L*-Chg

97 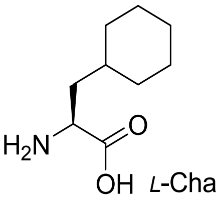 *L*-Cha

98 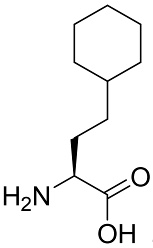 *L*-hCha

99 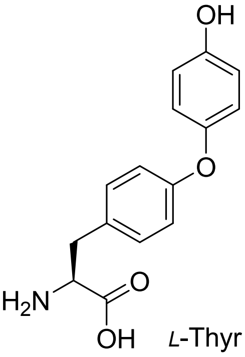 *L*-Thyr

100 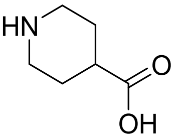 *L*-Inp

101 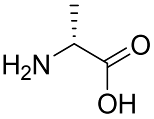 *D*-Ala

102 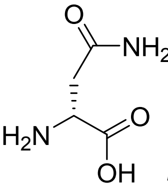 *D*-Asn

103 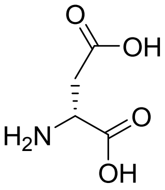 *D*-Asp

104 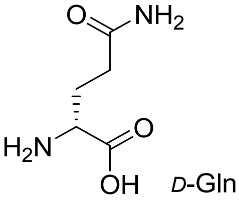 *D*-Gln

105 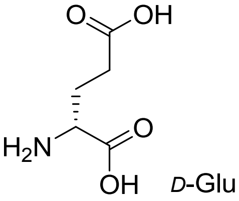 *D*-Glu

106 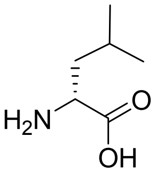 *D*-Leu

107 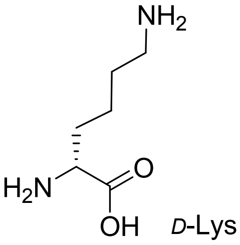 *D*-Lys

108 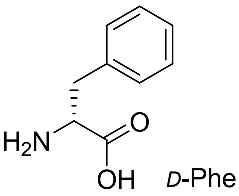 *D*-Phe

109 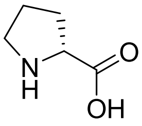 *D*-Pro

110 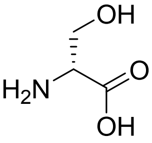 *D*-Ser

111 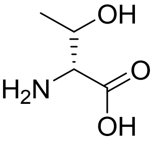 *D*-Thr

112 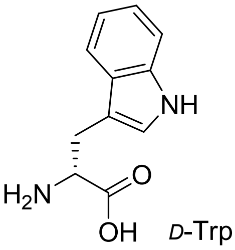 *D*-Trp

113 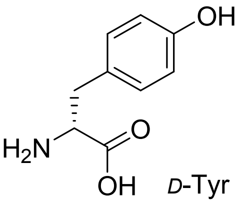 *D*-Tyr

114 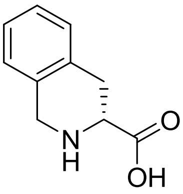 *D*-Tic

115 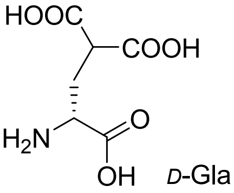 *D*-Gla

116 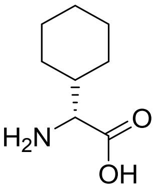 *D*-Chg

117 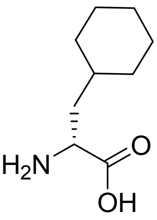 *D*-Cha

118 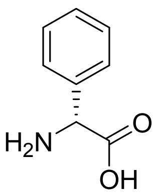 D-Phg

119 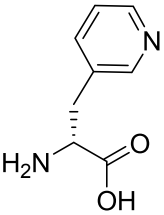 D-3-Pal

120 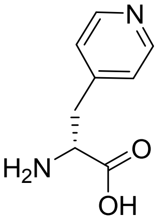 D-4-Pal

121 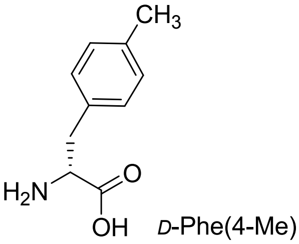 D-Phe(4-Me)

122 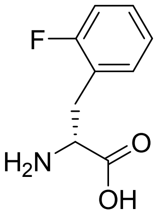 D-Phe(2-F)

123 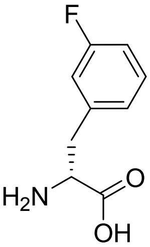 D-Phe(3-F)

124 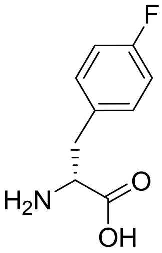 D-Phe(4-F)

125 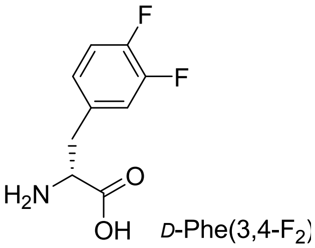 D-Phe(3,4-F_2)

126 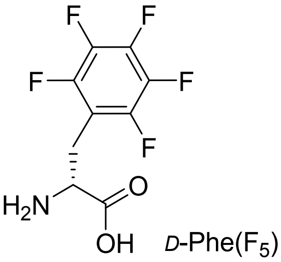 D-Phe(F_5)

127 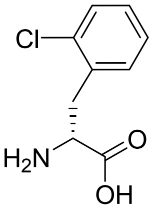 D-Phe(2-Cl)

128 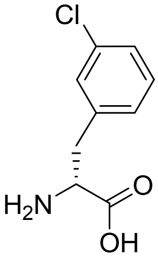 D-Phe(3-Cl)

129 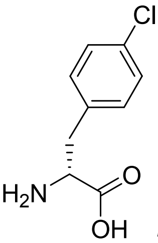 D-Phe(4-Cl)

130 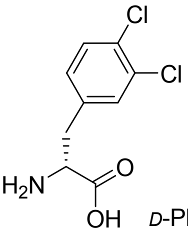 D-Phe(3,4-Cl_2)

131 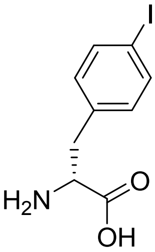 D-Phe(4-I)

132 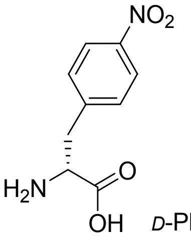 D-Phe(4-NO_2)

133 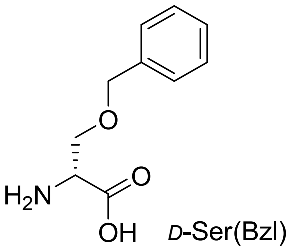 D-Ser(Bzl)

134 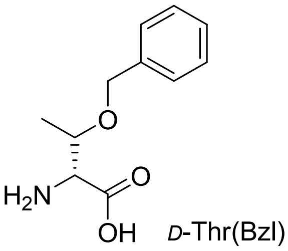 D-Thr(Bzl)

135 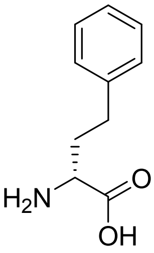 D-hPhe

136

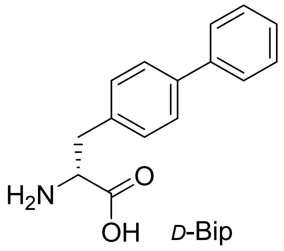

137

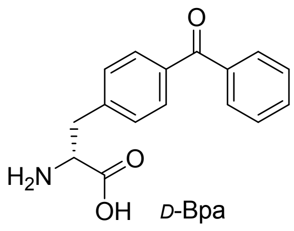

138

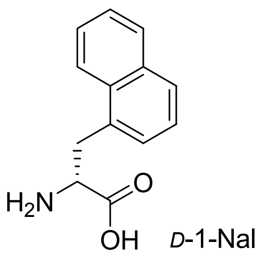

139

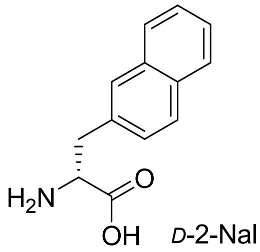

Table S10 The structures and codes of 139 amino acids used in the synthesis of Ac-ARL-P1-ACC library.

No.	Ac-ARL-P1-ACC	No.	Ac-ARL-P1-ACC	No.	Ac-ARL-P1-ACC
1	*L*-Ala	49	*L*-Phe(4-NH_2)	97	*L*-Cha
2	*L*-Arg	50	*L*-Phe(2-F)	98	*L*-hCha
3	*L*-Asn	51	*L*-Phe(3-F)	99	*L*-Thyr
4	*L*-Asp	52	*L*-Phe(4-F)	100	*L*-Inp
5	*L*-Gln	53	*L*-Phe(3,4-F_2)	101	*D*-Ala
6	*L*-Glu	54	*L*-Phe(F_5)	102	*D*-Asn
7	Gly	55	*L*-Phe(2-Cl)	103	*D*-Asp
8	*L*-His	56	*L*-Phe(3-Cl)	104	*D*-Gln
9	*L*-Ile	57	*L*-Phe(4-Cl)	105	*D*-Glu
10	*L*-Leu	58	*L*-Phe(3,4-Cl_2)	106	*D*-Leu
11	*L*-Lys	59	*L*-Phe(3-I)	107	*D*-Lys
12	*L*-Nle	60	*L*-Phe(4-I)	108	*D*-Phe
13	*L*-Phe	61	*L*-Phe(4-Br)	109	*D*-Pro
14	*L*-Pro	62	*L*-Phe(4-NO_2)	110	*D*-Ser
15	*L*-Ser	63	*L*-Phe(guan)	111	*D*-Thr
16	*L*-Thr	64	*L*-Phe(4-Me)	112	*D*-Trp
17	*L*-Trp	65	*L*-hPhe	113	*D*-Tyr
18	*L*-Tyr	66	*L*-Ala(2th)	114	*D*-Tic
19	*L*-Val	67	*L*-Ser(Bzl)	115	*D*-Gla
20	*L*-MeAla	68	*L*-Hse(Bzl)	116	*D*-Chg
21	beta-Ala	69	*L*-Thr(Bzl)	117	*D*-Cha
22	dhPro	70	*L*-Cys(Bzl)	118	*D*-Phg
23	*L*-Oic	71	*L*-Cys(MeBzl)	119	*D*-3-Pal
24	*L*-Hyp	72	*L*-Cys(4-MeOBzl)	120	*D*-4-Pal
25	*L*-Hyp(Bzl)	73	*L*-Tyr(Bzl)	121	*D*-Phe(4-Me)
26	*L*-Gla	74	*L*-Dht	122	*D*-Phe(2-F)
27	*L*-Asp(Me)	75	*L*-Trp(Me)	123	*D*-Phe(3-F)
28	*L*-Asp(All)	76	*L*-Tyr(Me)	124	*D*-Phe(4-F)
29	*L*-Asp(Bzl)	77	*L*-hTyr(Me)	125	*D*-Phe(3,4-F_2)
30	*L*-Glu(Me)	78	*L*-Tyr(2,6-Cl_2-Bzl)	126	*D*-Phe(F_5)

31	*L*-Glu(All)	79	*L*-Abu(Bth)	127	*D*-Phe(2-Cl)
32	*L*-Glu(Chx)	80	*L*-Bip	128	*D*-Phe(3-Cl)
33	*L*-Glu(Bzl)	81	*L*-Bpa	129	*D*-Phe(4-Cl)
34	*L*-Aad	82	*L*-Nle(O-Bzl)	130	*D*-Phe(3,4-Cl_2)
35	*L*-Api	83	*L*-1-Nal	131	*D*-Phe(4-I)
36	*L*-Dap	84	*L*-2-Nal	132	*D*-Phe(4-NO_2)
37	*L*-Orn	85	*L*-Hse	133	*D*-Ser(Bzl)
38	*L*-Cit	86	*L*-Hnv	134	*D*-Thr(Bzl)
39	*L*-hCit	87	*L*-Met	135	*D*-hPhe
40	*L*-Lys(Ac)	88	*L*-Met(O)	136	*D*-Bip
41	*L*-Lys(tfa)	89	*L*-Met$(O)_2$	137	*D*-Bpa
42	*L*-Lys(2Cl-Z)	90	*L*-Abu	138	*D*-1-Nal
43	*L*-His(Bzl)	91	*L*-Nva	139	*D*-2-Nal
44	*L*-Arg(Me)	92	*L*-Tle		
45	*L*-Arg$(Me)_2$	93	*L*-hLeu		
46	*L*-hArg	94	*L*-2-Aoc		
47	*L*-3-Pal	95	AC5C		
48	*L*-4-Pal	96	*L*-Chg		

Table 11 The list of amino acids used in the P1 Ac-Ala-Arg-Leu-X-ACC library.

1	L-Ala	41	L-Oic	81	L-Phe(4-Br)
2	L-Arg	42	L-Idc	82	L-Phe(4-Me)
3	L-Asn	43	L-Pip	83	L-3-Pal

4	L-Asp	44	L-Tic	84	L-4-Pal
5	L-Gln	45	dhAbu	85	L-Ala(2th)
6	L-Glu	46	dhLeu	86	L-Ala(Bth)
7	Gly	47	L-Dap	87	L-Bta
8	L-His	48	L-Dab	88	L-Abu
9	L-Ile	49	L-Dab(Z)	89	L-Abu(Bth)
10	L-Leu	50	L-Cit	90	L-Ser(Ac)
11	L-Lys	51	L-hCit	91	L-Ser(Bzl)
12	L-Nle	52	L-Orn	92	L-hSer
13	L-Phe	53	L-Lys(TFA)	93	L-hSer(Bzl)
14	L-Pro	54	L-Lys(Ac)	94	L-Thr(Bzl)
15	L-Ser	55	L-Lys(2-ClZ)	95	L-Cys(Bzl)
16	L-Thr	56	L-Agp	96	L-Cys(MeBzl)
17	L-Trp	57	L-Agb	97	L-Cys(4-MeOBzl)
18	L-Tyr	58	L-Arg(NO2)	98	L-Nle(O-Bzl)
19	L-Val	59	L-Arg(Z)2	99	L-Phg
20	D-Ala	60	L-hArg	100	L-hPhe
21	D-Arg	61	L-His(3-Bom)	101	L-Chg
22	D-Asn	62	L-Phe(NH2)	102	L-Cha
23	D-Asp	63	L-Phe(guan)	103	L-hCha
24	D-Gln	64	L-Trp(Me)	104	L-Igl
25	D-Glu	65	L-Dht	105	L-1-Nal
26	D-His	66	L-Asp(Me)	106	L-2-Nal
27	D-Leu	67	L-Asp(Chx)	107	L-Bpa
28	D-Lys	68	L-Asp(Bzl)	108	L-2-Aoc
29	D-Phe	69	L-Glu(Me)	109	L-hLeu
30	D-Pro	70	L-Glu(Chx)	110	L-NptGly
31	D-Ser	71	L-Glu(Bzl)	111	L-Hnv
32	D-Phg	72	L-Phe(2-F)	112	L-Tle
33	D-Thr	73	L-Phe(3-F)	113	L-Tyr(Me)
34	D-Trp	74	L-Phe(4-F)	114	L-Tyr(2,6Cl2-Z)
35	D-Tyr	75	L-Phe(3,4-F2)	115	L-Tyr(Bzl)
36	D-Val	76	L-Phe(F5)	116	L-Tyr(2-Br-Z)
37	β-Ala	77	L-Phe(2-Cl)	117	L-hTyr
38	L-Hyp	78	L-Phe(3-Cl)	118	L-hTyr(Me)
39	L-Hyp(Bzl)	79	L-Phe(4-Cl)	119	L-Nva
40	L-Thz	80	L-Phe(3,4-Cl2)	120	L-Met(O)

Table S12 The list of amino acids used for the synthesis of P4-P2 Hybrid Combinatorial Substrate Library with the fixed Arg in P1 and ACC in P1` position.

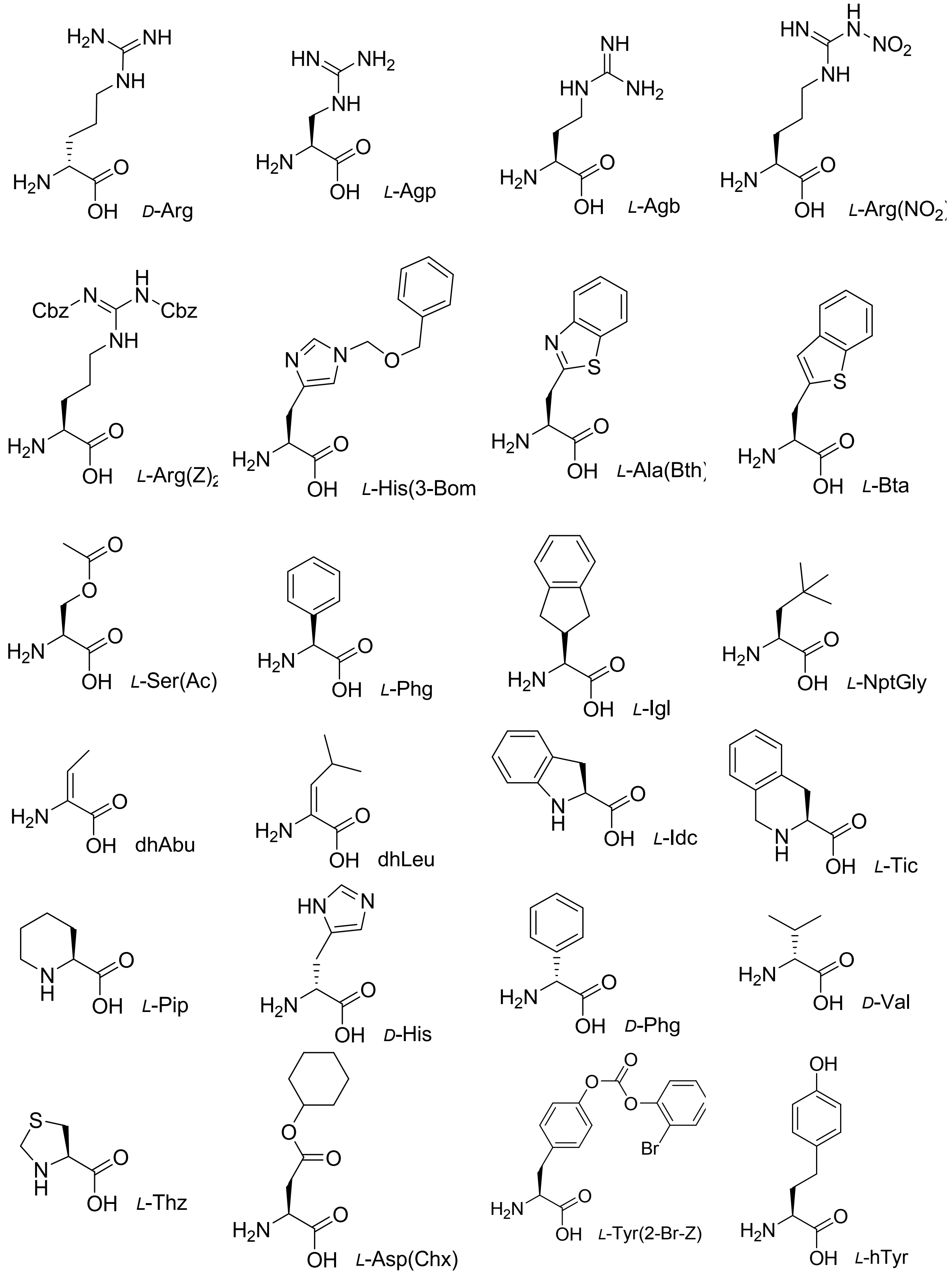

Table S13 The structures of amino acids that were also used In the HyCoSuL, but were not presented In Table 7.
